# A Shift in the Thermoregulatory Curve as a Result of Selection for High Activity-Related Aerobic Metabolism

**DOI:** 10.3389/fphys.2017.01070

**Published:** 2017-12-18

**Authors:** Clare Stawski, Paweł Koteja, Edyta T. Sadowska

**Affiliations:** ^1^Faculty of Biology and Earth Sciences, Institute of Environmental Sciences, Jagiellonian University, Kraków, Poland; ^2^Department of Biology, Norwegian University of Science and Technology, Trondheim, Norway

**Keywords:** bank vole, body temperature, endothermy, evolution, mammals, metabolic rate, thermal conductance, thermoneutral zone

## Abstract

According to the “aerobic capacity model,” endothermy in birds and mammals evolved as a result of natural selection favoring increased persistent locomotor activity, fuelled by aerobic metabolism. However, this also increased energy expenditure even during rest, with the lowest metabolic rates occurring in the thermoneutral zone (TNZ) and increasing at ambient temperatures (T_a_) below and above this range, depicted by the thermoregulatory curve. In our experimental evolution system, four lines of bank voles (*Myodes glareolus*) have been selected for high swim-induced aerobic metabolism and four unselected lines have been maintained as a control. In addition to a 50% higher rate of oxygen consumption during swimming, the selected lines have also evolved a 7.3% higher mass-adjusted basal metabolic rate. Therefore, we asked whether voles from selected lines would also display a shift in the thermoregulatory curve and an increased body temperature (T_b_) during exposure to high T_a_. To test these hypotheses we measured the RMR and T_b_ of selected and control voles at T_a_ from 10 to 34°C. As expected, RMR within and around the TNZ was higher in selected lines. Further, the T_b_ of selected lines within the TNZ was greater than the T_b_ of control lines, particularly at the maximum measured T_a_ of 34°C, suggesting that selected voles are more prone to hyperthermia. Interestingly, our results revealed that while the slope of the thermoregulatory curve below the lower critical temperature (LCT) is significantly lower in the selected lines, the LCT (26.1°C) does not differ. Importantly, selected voles also evolved a higher maximum thermogenesis, but thermal conductance did not increase. As a consequence, the minimum tolerated temperature, calculated from an extrapolation of the thermoregulatory curve, is 8.4°C lower in selected (−28.6°C) than in control lines (−20.2°C). Thus, selection for high aerobic exercise performance, even though operating under thermally neutral conditions, has resulted in the evolution of increased cold tolerance, which, under natural conditions, could allow voles to inhabit colder environments. Further, the results of the current experiment support the assumptions of the aerobic capacity model of the evolution of endothermy.

## Introduction

One of the central issues in evolutionary physiology is the question of what mechanisms led to the evolution of complex adaptations. The evolution of endothermy, the internal production of heat, has been of particular interest as this mode of living not only provides benefits, but also harbors many costs (Bartholomew, 1982; Withers et al., 2016). Endothermy allowed birds and mammals to uncouple their lives from external sources of heat to become nocturnal and also to be active in cold habitats. However, to fuel such an existence endotherms must consume large amounts of food to provide enough energy to maintain a high and stable body temperature (T_b_). Therefore, the question of what selection forces have led to such an energetically wasteful strategy has been subject to vivid discussion for already several decades (e.g., Bennett and Ruben, 1979; Hayes and Garland, 1995; Farmer, 2000, 2003; Koteja, 2000, 2004; Angilletta and Sears, 2003; Grigg et al., 2004; Kemp, 2006; Geiser, 2008; Clarke and Pörtner, 2010; Lovegrove, 2012, 2016). According to one of the hypotheses, the aerobic capacity model, high basal metabolic rates (BMR), i.e., a key feature of mammalian and avian endothermy, evolved as a correlated response to selection for high perpetual locomotion fueled by aerobic metabolism (Bennett and Ruben, 1979). The assumption of a positive correlation between aerobic capacity and BMR has been subject to numerous comparative, individual-level phenotypic and quantitative genetic analyses, which have generally provided convincing support (e.g., Hayes and Garland, 1995; Sadowska et al., 2005; Auer et al., 2017). However, to our knowledge, the question of how selection for high aerobic exercise performance affects other thermoregulatory traits has not been intensively studied. Therefore, the present study is based on a unique experimental evolution model system, with lines of the bank vole (*Myodes glareolus*) selected for several generations toward an increased exercise-induced aerobic metabolism (Sadowska et al., 2008; Konczal et al., 2015). In our previous reports, we showed that the selection indeed resulted in an increased BMR (Sadowska et al., 2015) and increased thermogenic capacity (the maximum cold-induced rate of oxygen consumption; Dheyongera et al., 2016). Here we ask, how the selection affected the “thermoregulatory curve” and other thermoregulatory traits.

**Figure 1:**
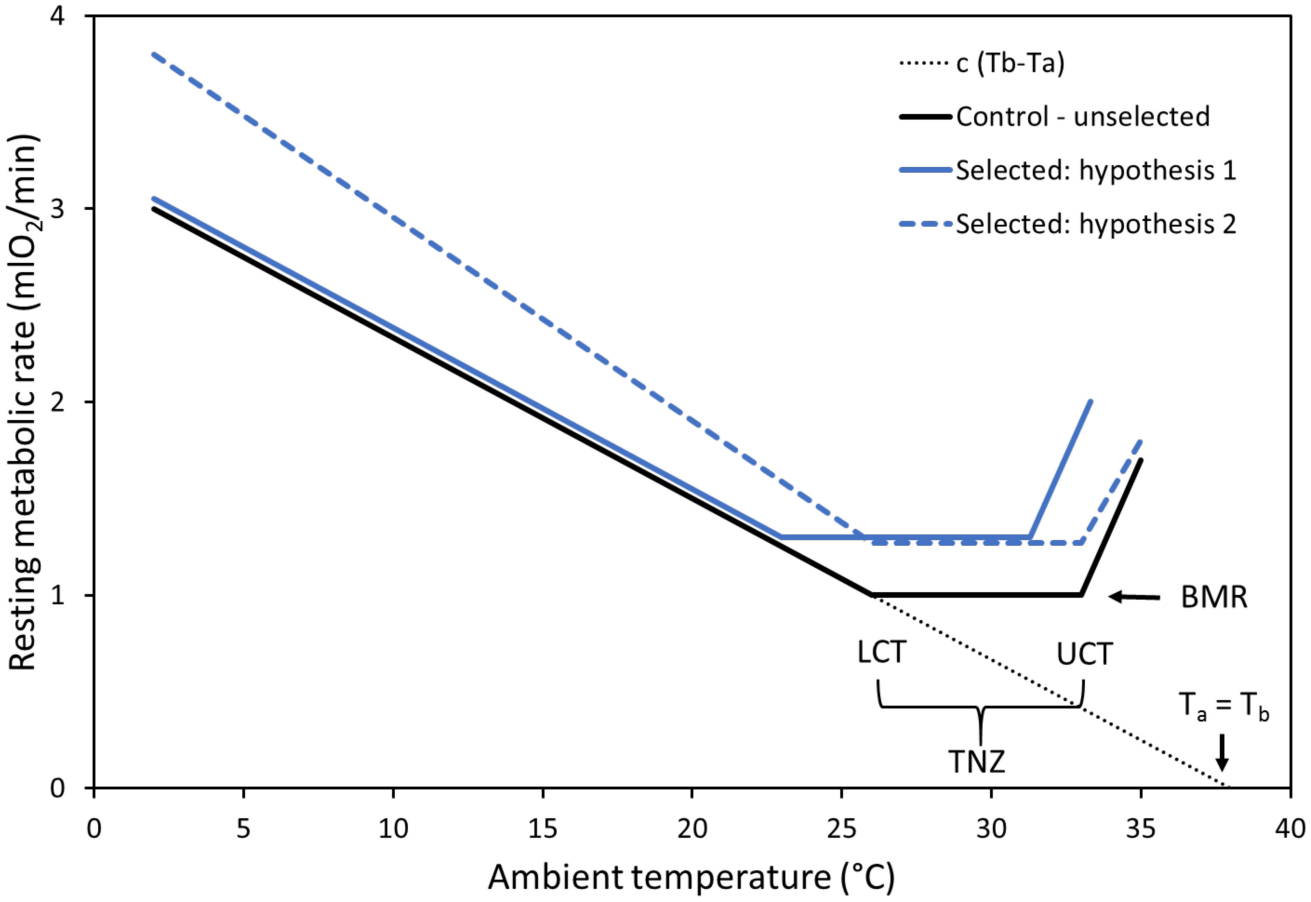
Graphical representation of the thermoregulatory curve and hypotheses concerning changes in response to selection for high activity-related metabolism. Control (C) lines are represented by the solid black line, with the thermoneutral zone (TNZ) depicted and encompassed by the lower critical temperature (LCT) and upper critical temperature (UCT). As we already know that BMR is increased in selected (A) lines, RMR in the TNZ should also be increased (blue lines). The solid blue line represents hypothesis 1, according to which in the A lines: (1a) both the LCT and the UCT are markedly shifted downwards, (1b) above the UCT the increase of RMR is higher, and (1c) below the LCT the thermoregulatory curve overlaps with that of the C lines. The dashed blue line represents hypothesis 2, according to which in the A lines: (2a) the position of the TNZ remains unaffected, (2b) above the UCT the increase of RMR is less steep, and (2c) below the LCT the thermoregulatory curve is steeper than in the control lines. See Introduction for justification of the hypothetical patterns. The dotted line represents heat balance = 0, condition extended to body temperature (T_b_) equal to ambient temperature (T_a_).

The thermoregulatory curve (Figure 1), also known as the Scholander-Irving model, depicts the pattern of changes of resting metabolic rate (RMR) of an endothermic homeotherm over a range of ambient temperatures (T_a_) (Scholander et al., 1950; McNab, 2002; Riek and Geiser, 2013; Levesque et al., 2016). In a range of T_a_ termed the thermoneutral zone (TNZ), heat balance can be maintained without producing extra heat above the level of BMR. At T_a_ below the lower boundary of TNZ (i.e., the lower critical temperature; LCT), RMR increases to compensate for greater heat loss. According to a simplified linear model, the heat loss, and hence RMR, is proportional to a total thermal conductance coefficient (*c*), which incorporates both the properties of thermal insulation of the animal's body and characteristics of evaporative heat loss: RMR = *c*(T_b_−T_a_) (McNab, 2002). If heat loss exceeds the thermogenic capacity of the animal, hypothermia occurs. On the other hand, at T_a_ above the upper boundary of the TNZ (i.e., the upper critical temperature; UCT), costly mechanisms of dissipating excess heat must be engaged to avoid overheating, such as increased evaporative cooling and increased blood circulation to distal body parts (these are depicted by the entire thermoregulatory curve model and cannot be explained by the Scholander-Irving model itself). Such processes, as well as passive thermodynamic effects due to an increased T_b_, result in an elevated RMR, and consequently an enhanced thermoregulatory burden. Therefore, the increase of RMR above the TNZ is typically more rapid than that below the TNZ, and animals may become severely hyperthermic at T_a_'s just above the TNZ.

In the current study we aimed to quantify how the above-described thermoregulatory characteristics changed in lines of bank voles subject to selection for high rates of oxygen consumption achieved during swimming ($\dot{\text{V}}$O_2_swim; Sadowska et al., 2008, 2015, 2016). In generations 11–14 of the selection experiment, voles from the selected “Aerobic” (A) lines achieved about a 50% higher $\dot{\text{V}}$O_2_swim than those from unselected, control (C) lines. Not surprisingly, both the spontaneous locomotor activity in cages and the maximum forced-running oxygen consumption (the aerobic capacity *per se*) were also increased in the A lines (Jaromin et al., 2016a), as well as some other morphological and biochemical traits related to exercise metabolism (Stawski et al., 2015b; Jaromin et al., 2016b). Importantly, the swimming trials are performed at 38°C, therefore the direct selection is imposed on locomotor performance only, and not on thermoregulatory capability. According to the aerobic capacity model, however, we predicted that the evolution of aerobic exercise performance should also drive the evolution of thermoregulatory properties. Indeed, voles from the A lines evolved also a 7.3% higher mass-adjusted BMR, increased rate of food consumption (and hence presumably an increased average daily heat production), and increased thermogenic capacity (Sadowska et al., 2008, 2015; Dheyongera et al., 2016). Thus, a few traits crucial in shaping the thermoregulatory curve have changed in response to selection for aerobic exercise performance (although others, namely the capacity for nonshivering thermogenesis, NST, remained unaffected; Stawski et al., 2015a). Therefore, we hypothesize that other characteristics, especially the boundaries of the TNZ, thermal conductance, and T_b_ at high T_a_ have also changed.

As we already know that BMR (i.e., RMR measured in the TNZ in fasted animals; McNab, 2002), measured at a T_a_ of 28°C chosen to be plausibly within the TNZ of voles based on published data (Górecki, 1968), increased in the A lines (Sadowska et al., 2015), a straightforward expectation is that RMR measured at T_a_ around 28°C should be also higher in the A than in the C lines. However, further predictions can be only conditional, depending on how thermal conductance and T_b_ have changed (Figure 1). Under the laboratory conditions of our selection experiment, the voles are housed at 20°C, i.e., at a temperature below the TNZ. Thus, there is no strong argument to expect that increased BMR should result in a change of thermal insulation in the A lines. If this is the case we should expect that in the A lines: (1a) both the LCT and the UCT will be markedly shifted downwards (although not necessarily by the same amount, as the values are determined by distinct physical and physiological process), (1b) as a consequence of a lower UCT, at a particular T_b_ above the UCT the increase of RMR will be higher and a more profound hyperthermia will occur, and (1c) below the LCT the thermoregulatory curve will overlap with that of the C lines. However, if the thermal insulation of the selected voles has decreased proportionally to the increase of BMR, and they evolved a more efficient mechanism for dissipating excess heat by evaporative cooling or transferring excess heat to distal body parts, we can expect that in the A lines: (2a) the position of the TNZ will remain unaffected, (2b) above the UCT the increase of RMR will be less steep, and (2c) below the LCT the thermoregulatory curve will be steeper than in the C lines as a result of higher heat loss. If, on the other hand, voles from the A lines have an increased T_b_ yet their thermal insulation properties have not changed, then we might expect that: (3) the entire thermoregulatory curve will be shifted upwards, but the lines will remain parallel. Still more scenarios can be envisioned if we consider the possibility that both thermal insulation and T_b_ have evolved. Thus, even though the physical process we consider is relatively simple and technically “hard” predictions can be formulated, the experiment has, inevitably, an exploratory nature.

## Materials and methods

This study was undertaken on bank voles (*M. glareolus*) from the 13th and 14th generations of the artificial selection experiment. Information about the base population, the rationale of the ongoing experiment, selection protocol, and direct response to the selection has been presented in our earlier work (Sadowska et al., 2008, 2015; Konczal et al., 2015). To summarize, the base colony was founded using ~320 voles captured in the Niepołomice Forest in southern Poland in 2000 and 2001. The animals were bred randomly for 6–7 generations, and the colony was used for quantitative genetic analyses of metabolic rates (Sadowska et al., 2005). In 2004, the multidirectional selection experiment was established (Sadowska et al., 2008). In the selected “Aerobic” (A) lines used in this current work, the selection criterion was the maximum mass-independent (residual from regression) 1-min rate of oxygen consumption achieved during an 18-min swimming trial, performed at the age of 75–85 days. The swim test was conducted at 38°C, at a temperature close to T_b_ of the voles, so that no thermoregulatory burden was imposed (neither excessive heat loss nor overheating load; see Supplementary Material 1). The test was terminated earlier than the maximum 18-min if an animal was struggling to swim, irrespective of the selection direction of the individual. Four replicate A-selected lines and four unselected Control (C) lines are maintained (to allow valid tests of the effects of selection; Henderson, 1997), with 15–20 reproducing families in each of the eight lines (which prevents excess inbreeding). The selection was applied mostly within families, but if more than 16 families were available, families in which all individuals scored below the adjusted line mean were excluded. The animals were kept in standard plastic mouse cages with sawdust bedding at a constant temperature (20 ± 2°C) and photoperiod (16:8 L:D) and supplied with food (a standard rodent chow: 24% protein, 3% fat, 4% fiber; Labofeed H, Kcynia, Poland) and water *ad libitum*. All of the procedures associated with the breeding scheme and the selection protocol were approved by the Local Bioethical Committee in Kraków, Poland (No. 99/2006, 21/2010, and 22/2010).

One week before measurements of RMR all individuals were implanted with miniature data loggers to measure T_b_ (resolution 0.125°C, iButton thermochron DS1921H, Maxim Integrated Products, Inc., Sunnyvale, California, USA). These data loggers were programmed to record T_b_ every 5-min yielding ~2,144 data points per animal (3 iButtons malfunctioned and no data were retrieved). Data loggers were coated in wax (mean total mass: 2.43 g) and calibrated over a temperature range of 15–43°C against an Albhorn precision thermometer (Albhorn Therm 2244-1, probe: NTC type C 856-1). The procedure was performed as described in Jefimow and Wojciechowski (2014). The surgery was performed under Nembutal (95 mg kg^−1^; Morbital, Biowet, ZAP, Poland) anesthesia in voles from generation 13 or under ketamine (40 mg kg^−1^; Ketamine 10%, Biowet, Puławy, Poland) followed by xylazine (8 mg kg^−1^; Sedazin 2%, Biowet, Puławy, Poland) in voles from generation 14. A 1 to 1.5-cm incision was made to the skin and muscle layers and a sterilized (95% alcohol) logger was inserted into the abdominal cavity. The muscle and skin were sutured using absorbable suture (Safil 5/0, AesculapAG, Tuttlingen, Germany) and voles were provided water containing the antibiotic enrofloxacin (50 mg L^−1^) *ad libitum*. Post-surgical care was continued for the next 3 days.

RMRs of voles were measured as rates of O_2_ consumption (mLO_2_ min^−1^) at T_a_ ranging from 10 to 34°C (10°C: selected *n* = 61, control *n* = 61; 20°C: selected *n* = 71, control *n* = 71; 25–34°C: selected *n* = 32, control *n* = 32). Throughout all experiments T_a_ was measured once every 10-min with data loggers (the same type as used for T_b_) placed in the experimental chambers.

Four hours before measurements the voles were weighed and placed in plastic respirometric chambers (850 mL), without access to food or water, at the required T_a_ to allow animals to acclimatize to the chambers. The chambers were fitted with wire tops suspended 3 cm below the ceiling of the chamber. With the air inlet near the bottom and the outlet at the top of the chamber. This was to ensure that the voles could not exhale air directly into the outgoing air and the incoming air was mixed with the air in the chamber.

The measurements were performed at two time intervals (the actual timing varied ±0:30 h from the following values): the “Morning” group of voles were placed in the chambers at 06:00, the chambers were connected to the system only at 10:00 and the recordings continued until 13:00. The “Afternoon” group was placed in the respirometric chambers at 09:00, the chambers were connected to the system only at 13:00 and the recordings continued until 16:00. The “Timing” group was included as a cofactor in all statistical analyses.

Rates of oxygen consumption ($\dot{\text{V}}$O_2_) were measured using an open-flow positive-pressure respirometric system. Fresh air was dried (silica gel) and pumped into the chambers containing the animals. The rate of air flowing into the chambers was stabilized at either 350 mL min^−1^ (for T_a_ of 20–34°C) or 450 mL min^−1^ (for T_a_ of 10°C) (STPD) with thermal mass-flow controllers (Alborg, Orangeburg, NY, USA). The actual flow was corrected after calibrating the mass-flow controllers against a precise LO 63/33 rotameter (Rota, Germany). Samples of air flowing out of the animal chamber were pre-dried with ND2 non-chemical drier (Sable Systems Inc.), dried with a small volume of chemical absorbent (magnesium perchlorate) and passed through the O_2_ analyzers. Mean values of analog outputs from the O_2_ analyzer were recorded once per second with Lab Jack UE-9 AD interface and a custom-made protocol using DAQ Factory acquisition system (Azotech, Ashlans, OR, USA). $\dot{\text{V}}$O_2_ was calculated according to equation 1b in Koteja (1996). We assumed RQ equals 0.85, which was confirmed by measurements performed together with a CO_2_ analyzer in a subset of the animals.

Two experimental setups were used, one for stable temperatures of 10 and 20°C and another for increasing T_a_ from 25 to 34°C.

The rates of oxygen consumption for T_a_ of 10 and 20°C were measured with a five-channel respirometric system with a FOX O_2_ analyzer (Sable Systems Inc. Las Vegas, NV, USA). Samples of air flowing out of a reference (empty) and four measurement chambers (with animals) were analyzed sequentially, in a 13-min cycle. In each cycle, the reference channel and the first measurement channel were active for 165-s, and the remaining three measurement channels were active for 150-s, which ensured a complete washout of the system after switching channels (the time was longer for the reference and the first measurement channels because the change of air composition after switching to those channels is larger than in the case of the other channels). The last 20-s before switching channels was used to calculate the rate of O_2_ uptake. Importantly, as the air flow/chamber volume ratio was low (0.44), the last 20-s effectively represented a signal integrated from a longer period.

For the second protocol only two animals were measured simultaneously during each trial during which T_a_ was increased from 25 to 34°C in 3°C increments. Throughout the measurements animals were kept at 25 and 28°C for 1 h and at 31 and 34°C for 30-min. The shorter time periods at high T_a_ were to prevent hyperthermia, particularly in individuals from the A lines. The rates of oxygen consumption for T_a_ 25 to 34°C were measured continuously with either a FOX O_2_ analyzer or FC-10a analyzer (Sable Systems Inc. Las Vegas, NV, USA). The rate of O_2_ uptake was obtained for the lowest 1-min reading for each experimental T_a_.

Maximum thermogenic capacity ($\dot{\text{V}}$O_2_cold) was measured as the rate of oxygen consumption (ml min^−1^) in completely soaked individuals placed in a wet chamber for up to 18-min at +23°C (procedure similar to Sadowska et al., 2005 and Dheyongera et al., 2016). The voles were weighed, soaked in warm (+38°C) water containing a drop of dog shampoo to ensure complete saturation and then placed in wet respirometric chambers (500 mL) maintained at +23°C in a temperature-controlled cabinet (PTC-1 Peltier; Sable Systems, Las Vegas, NV, USA). The respirometric chambers were connected to one of two separate open-flow, positive pressure respirometric systems. The airflow rate through the chambers (about 2,000 mL min^−1^ at standard temperature and pressure), was controlled to ±1% with mass flow controllers (either Model ERG3000, Beta-Erg, Warsaw, Poland: or Model GFC-171S, Aalborg Instruments, Orangeburg, NY, USA). Excurrent air was pre-dried with ND2 non-chemical drier (Sable Systems, Las Vegas, NV, USA) or DG-1 Dewpoint Generator with Pelt-4 Condenser PC-2 (Sable Systems, Las Vegas, NV, USA) and dried with a small volume of chemical absorbent (magnesium perchlorate) and passed through the O_2_ analyzers. (FC-10A or FC2 Oxzilla: Sable Systems, Las Vegas, NV, USA). In both systems, the concentration of gases was recorded every second with UI2 (Sable Systems, Las Vegas, NV, USA) interface and protocol using Expedata acquisition system. Thermogenic capacity was defined as the highest 1-min instantaneous rates of oxygen consumption (Bartholomew et al., 1981; effective volume of the chambers was 650 and 700 mL, respectively). At the end of each trial we measured rectal temperature (T_b_cold) using an Albhorn thermometer (Albhorn Therm 2244-1, probe: NTC type C 856-1).

Two values of T_b_ were calculated from the data: (1) T_b_mean is the mean T_b_ from 30-min of data that were recorded 4.5–5-h after putting the animal in the chamber and (2) T_b_rmr is the T_b_ recorded at the time of the lowest RMR measurement. From measurements of T_b_rmr and RMR we also calculated the thermal conductance [CT, mLO_2_/(min × °C)] of the voles at each of the measurement T_a_s:

(1) $$\text{CT}=\text{RMR}/({\text{T}}_{\text{b}}\text{rmr}-{\text{T}}_{\text{a}})$$

For statistical analyses we used SAS (v. 9.4, SAS Institute, Inc., Cary, NC, USA). To compare T_b_mean, T_b_rmr, RMR and CT of voles from A and C lines at each of the measurement T_a_s and also for T_b_cold and $\dot{\text{V}}$O_2_cold from the maximum thermogenic capacity trials we applied a cross-nested Mixed ANCOVA model implemented with the Mixed Procedure (with REML method) with Selection (A vs. C) as the main, top-level fixed factor, and replicated Lines as a random effect nested within Selection. Further, we also included fixed cofactors and covariates: Sex, Generation (13 or 14), Timing (Morning or Afternoon), Age, and Body Mass. The model included also a fixed interaction of Selection × Sex and the random interaction of Sex × Line. Values that were obtained from active individuals were omitted from the analyses. Additionally, studentized residuals were analyzed and observations with residuals below −3 or above 3 were considered outliers and removed from the final analyses.

Next, we used a repeated-measures extension of the above model to perform analyses for combined results from the trial performed at temperatures around the TNZ (25, 28, 31, and 34°C). In addition to the factors described above, the model included a fixed repeated-measures factor for T_a_ (treated as a grouping factor), interactions of T_a_ with Selection, Sex and Line, and the random effect of Individual (“subject”). As the analyses performed separately for each T_a_ revealed large differences of residual variance, in the repeated measures model an “unstructured” type of residual (co)variance matrix was assumed. To compare the four T_a_ groups, Tukey-Kramer post-hoc tests were performed. A similar but simpler model (with no interactions between T_a_ and other factors and compound symmetry variance structure) was used to analyze initial body mass measured before the three trials (at 10, 20, and 25–34°C). In all of the above analyses, variance was constrained to non-negative values (default approach in SAS), and Satterthwaite approximation for non-orthogonal models was applied to calculate the denominator degrees of freedom.

Finally, to analyze the main characteristics of the thermoregulatory curve we applied a stage-regression model, implemented in SAS mixed nonlinear procedure (NLMIXED). The data available could not allow a reliable estimate of the UCT and therefore the analyses were performed only for the temperature range of 10–31°C, and was focused on the question of whether selection affected LCT, the level of RMR above LCT (RMR_TNZ_; conceptually equivalent to BMR), and the slope of the relationship between RMR and T_a_ below LCT (*C*_*t*_; i.e., another measure of thermal conductance). The model includes also random effects of individuals (ID) and residual error e, each assumed to have a normal distribution. The logic of the model was as follows:

(2) $$\text{RMR}=\{\begin{array}{l} \text{for }{\text{T}}_{\text{a}}>=\text{LCT}:\;{\text{RMR}}_{\text{TNZ}}+\text{ID}+\text{e}\\ \begin{array}{l} \text{for }{\text{T}}_{\text{a}}<\text{LCT}:\;{\text{RMR}}_{\text{TNZ}}+\text{CT}\times \left(\text{LCT}-{\text{T}}_{\text{a}}\right)\\ +\text{ID}+\text{e} \end{array} \end{array}$$

However, all of the three parameters of the model are known to depend on body mass (e.g., McNab, 2002). Therefore, they were introduced to the model as linear functions of body mass (M_b_), each with an intercept and a mass-slope coefficient:

(3a) $$\text{LCT}=t_{0}+t_{m}\times M_{b}$$

(3b) $${\text{RMR}}_{\text{TNZ}}=b_{0}+b_{m}\times M_{b}$$

(3c) $$\begin{array}{cc} {\text{RMR}}_{\text{TNZ}}=b_{0}+b_{m}\times M_{b} & \left(3\text{b}\right) \end{array}$$

Finally, each of the six parameters were introduced to the model as either a value common for both of the selection directions or specific for the A and C lines. Thus, the initial “full” model had a total of 12 fixed parameters (in addition to two random effects), i.e., allowed not only difference in intercepts between the selection directions, but also heterogeneous mass-slopes. The model was then stepwise reduced, first by removing the coefficients responsible for differences in mass-slope coefficients (which resulted in a model with homogeneous mass-slopes), and then by removing other components. We first compared the models using AIC criterion, and then to formally test significance of difference in a particular parameter between the A and C lines a likelihood ratio test (LRT) was applied.

Here we present adjusted least square means (with standard error) for the main factor only (Selection). In the supplementary files we also provide the complete tables with descriptive statistics and results of the mixed ANCOVA models and NLMIXED (Supplementary Material 2) and also raw data (Supplementary Material 3).

## Results

Body mass (M_b_) measured before the three respirometric trials (at 10, 20, and 25–34°C) increased with age [*F*_(1, 148)_ = 4.91, *p* = 0.028] and was on average 0.32 ± 0.15 g lower in the afternoon than in the morning trials [*F*_(1, 215)_ = 4.19, *p* = 0.042], but did not differ between generations [*F*_(1, 135)_ = 0.94, *p* = 0.33] or the three trials [*F*_(2, 198)_ = 0.96, *p* = 0.39]. The M_b_ adjusted for these cofactors was higher in the selected (A) than in control (C) lines, and higher in males than in females [LSM ± SE for the age of 140 days; A line females: 24.6 ± 0.9 g, males: 28.0 ± 0.9 g, C line females: 21.6 ± 0.9 g, males 25.3 ± 0.9 g; effect of selection: *F*_(1, 6.1)_ = 6.55, *p* = 0.042; effect of sex: *F*_(1, 5.6)_ = 39.8, *p* = 0.001].

**Figure 2:**
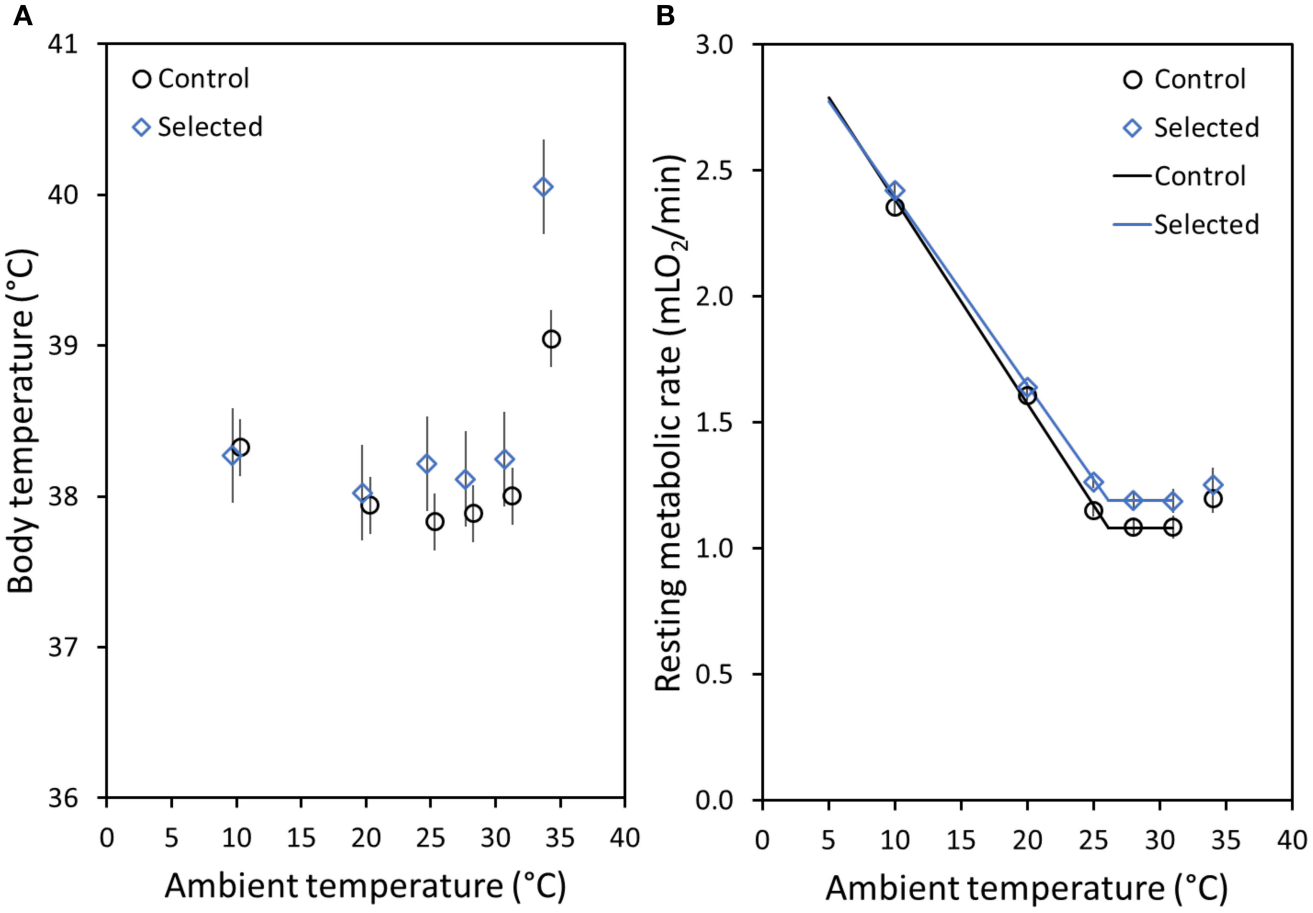
**(A)** Mean body temperatures (T_b_rmr) of bank voles at the time of the lowest measured resting metabolic rate (RMR) at each measurement temperature (T_a_) for control (C: black dots) and selected (**A**: blue diamonds) lines. **(B)** Mean RMR of bank voles at each measurement T_a_ for the C (black dots) and the A (blue diamonds) lines. For both figures means are the adjusted least square means (LSM calculated for a fixed body mass, 25 g, and age, 140 days) and the whiskers above and below each mean value represent the standard error (note: for most RMR results the SE range is not visible, because it is smaller than the size of the symbols). The lines on **(B)** represent the best-fit model, with a common lower critical temperature (LCT = 26.1 ± 0.27°C), but different levels of RMR above LCT (RMR_TNZ_, for a 25 g vole; A lines: 1.19 mLO_2_/min, C lines: 1.08 mLO_2_/min), and different slopes [C_t_, for a 25 g vole; A lines: 0.055 mLO_2_/(min × °C), C lines: 0.061 mLO_2_/(min × °C)].

The results for T_b_mean and T_b_rmr were similar and here we only present the results for T_b_rmr (see Table 1 for values of both variables). T_b_rmr was elevated in the A lines at higher T_a_ but not at lower T_a_, in comparison to the C lines. Specifically, at 10°C T_b_rmr was virtually identical in the lines, and at 20°C it was only 0.08°C higher in the A lines (*p* > 0.71; Table 1, Figure 2A). At T_a_s around the TNZ, T_b_rmr tended to be higher in the A than in the C lines (at 25°C: 0.38°C difference, at 28°C: 0.23°C, at 31°C: 0.25°C), but the difference was nearly significant only at 25°C (*p* = 0.07; otherwise *p* > 0.44; Table 1, Figure 2A). The difference in T_b_rmr between the A and C lines was greatest at the T_a_ of 34°C (1.01°C), but because individual variance dramatically increased the difference was still not significant (*p* = 0.15; Table 1, Figure 2A). However, the repeated measures analysis performed for combined T_a_s around TNZ (25, 28, 31, and 34°C) showed that T_b_rmr, averaged across the four temperatures, was clearly significantly higher in the A than in the C lines [A lines = 38.72 ± 0.13°C; C lines = 38.12 ± 0.12; effect of Selection: *F*_(1, 8.7)_ = 10.8, *p* = 0.009]. The analysis showed also that T_b_rmr averaged across the A and C lines was similar at 25°C (38.03°C) and 28°C (38.01°C), slightly increased at 31°C (38.13°C), and increased significantly at 34°C [39.51°C; effect of T_a_: *F*_(3, 55.9)_ = 24.0, *p* < 0.0001]. Further, the analysis also revealed a significant interaction between the effects of T_a_ and Selection [*F*_(3, 55.8)_ = 5.12, *p* = 0.003]; while the differences in T_b_rmr between the lines at T_a_ ranging from 25 to 31°C were similar (~0.3°C), at 34°C T_b_rmr in the A lines was more than 1.5°C higher than that in the C lines.

**Table 1:** Summary statistics showing values (adjusted least square means ± standard error, LSM ± SE) for control (C) and selected (A) lines for each measured variable for each experimental procedure.

**Trial**	**Variable**	**LSM ± SE**		**Significance of effects**						
		**Control (C)**	**Selected (A)**	**Selection**	**Sex**	Selection ^*^ sex	**Generation**	**Timing**	**Body mass**	**Age**
10°C	T_b_mean	38.51 ± 0.13	38.35 ± 0.13	0.12	0.57	0.16	0.79	0.87	0.75	0.34
	T_b_rmr	38.32 ± 0.14	38.27 ± 0.14	0.74	0.93	0.82	0.87	0.38	0.85	0.76
	RMR	2.36 ± 0.03	2.42 ± 0.03	0.19	0.07	0.74	0.95	0.13	**<0.0001**	0.74
	CT	0.08 ± 0.001	0.09 ± 0.001	0.44	0.31	0.63	0.76	0.35	**<0.0001**	0.44
20°C	T_b_mean	38.11 ± 0.08	38.11 ± 0.08	0.54	0.11	0.09	**0.02**	0.11	**0.02**	**0.01**
	T_b_rmr	37.94 ± 0.11	38.02 ± 0.11	0.72	0.35	0.16	**0.05**	0.80	**0.03**	**0.04**
	RMR	1.61 ± 0.02	1.64 ± 0.02	0.53	0.64	0.77	**0.001**	0.11	**<0.0001**	**0.02**
	CT	0.09 ± 0.001	0.09 ± 0.001	0.28	0.94	0.84	**0.002**	0.17	**<0.0001**	0.10
25°C	T_b_mean	38.07 ± 0.11	38.17 ± 0.11	0.61	0.18	0.96	0.43	0.99	0.28	0.18
	T_b_rmr	37.83 ± 0.12	38.22 ± 0.12	0.07	0.94	0.81	0.54	0.24	0.26	0.72
	RMR	1.15 ± 0.02	1.26 ± 0.02	0.19	0.44	0.09	0.96	0.57	**<0.0001**	0.42
	CT	0.09 ± 0.002	0.10 ± 0.002	0.56	0.69	0.08	0.77	0.92	**<0.0001**	0.37
28°C	T_b_mean	37.95 ± 0.12	38.26 ± 0.12	0.23	0.86	0.79	0.36	0.53	0.97	0.29
	T_b_rmr	37.887 ± 0.123	38.116 ± 0.124	0.447	0.69	0.732	0.461	0.306	0.975	0.32
	RMR	1.08 ± 0.04	1.19 ± 0.04	0.18	0.84	0.59	0.36	0.93	**<0.0001**	0.29
	CT	0.11 ± 0.002	0.12 ± 0.002	0.19	0.91	0.73	0.22	0.11	**<0.0001**	0.20
31°C	T_b_mean	38.18 ± 0.18	38.66 ± 0.18	0.27	0.45	0.75	0.88	0.59	0.29	0.99
	T_b_rmr	38.00 ± 0.13	38.25 ± 0.14	0.57	0.52	0.64	0.64	0.13	0.93	0.88
	RMR	1.09 ± 0.05	1.19 ± 0.05	0.15	0.10	0.69	0.36	0.28	**<0.0001**	0.57
	CT	0.15 ± 0.004	0.16 ± 0.005	0.15	0.23	0.58	0.43	**0.05**	**<0.0001**	0.67
34°C	T_b_mean	39.46 ± 0.40	40.08 ± 0.39	0.29	0.76	0.69	0.76	0.38	0.15	0.24
	T_b_rmr	39.05 ± 0.40	40.05 ± 0.39	0.15	0.66	0.78	0.42	0.97	**0.04**	0.57
	RMR	1.19 ± 0.06	1.25 ± 0.07	0.53	0.89	0.74	0.31	0.89	**<0.0001**	0.39
	CT	0.25 ± 0.007	0.24 ± 0.008	0.45	0.83	0.80	0.12	0.06	**<0.0001**	0.07
$\dot{\text{V}}$O_2_cold	T_b_cold	28.48 ± 0.20	28.60 ± 0.22	0.36	0.06	0.34	**0.03**	NA	**0.01**	0.59
	$\dot{\text{V}}$O_2_cold	4.20 ± 0.07	4.69 ± 0.07	**0.0003**	0.06	0.90	0.06	NA	**<0.0001**	0.25

*LSM are calculated for a fixed body mass (25 g) and age (140 days). Shown also are the significance of each of the effects, significant effects are shown in bold*.

*T_b_mean (°C) = mean body temperature of 30-min of data (data from 4.5 to 5-h after putting animal in the chamber); T_b_rmr (°C) = body temperature at time of lowest RMR; RMR (mLO_2_min^−1^) = lowest resting metabolic rate; CT [mLO_2_/(min × °C)] = thermal conductance; T_b_cold (°C) = T_b_ after maximum thermogenesis experiment; $\dot{\text{V}}$O_2_cold (mLO_2_min^−1^) = maximal MR during thermogenesis experiment; NA = not applicable to these measurements*.

At all measurement T_a_s, RMR increased with increasing body mass (*p* < 0.0001; Table 1). At T_a_s below the LCT (10 and 20°C), RMR did not differ between the A and C lines (*p* > 0.19; Table 1). Specifically, RMR for A lines was only 2.6% higher at 10°C and 2.1% higher at 20°C (Table 1, Figure 2B). In contrast, at measurement T_a_s between 25 and 31°C RMR was ~9% higher in the A than in the C lines, however, these differences were not significant (*p* > 0.15; Table 1, Figure 2B). At 34°C RMR in the A lines was 4.2% higher than in the C lines (Table 1, Figure 2B), but this difference was also not significant (*p* = 0.53). Yet, similarly to T_b_rmr, the repeated measures analysis performed for combined T_a_s around TNZ (25, 28, 31, and 34°C) revealed that RMR, averaged across the four temperatures, was significantly higher in the A than in the C lines [A lines = 1.24 ± 0.03 mLO_2_min^−1^; C lines = 1.12 ± 0.03 mLO_2_min^−1^; effect of Selection: *F*_(1, 19.4)_ = 7.65, *p* = 0.01]. Additionally, the RMR of the lines combined was significantly affected by T_a_ [*F*_(3, 8.5)_ = 12.08, *p* = 0.002]: it was higher at 25°C (1.21 ± 0.02 mLO_2_min^−1^) and 34°C (1.24 ± 0.03 mLO_2_min^−1^) in comparison to 28°C (1.14 ± 0.02 mLO_2_min^−1^) and 31°C (1.13 ±0.03 mLO_2_min^−1^). However, unlike for T_b_rmr, the interaction between Selection and T_a_ was not significant for RMR [*F*_(3, 8.5)_ = 0.32, *p* = 0.81], i.e., the differences between the A and C lines were consistent across the T_a_s (and *vice versa*).

Similarly to RMR, thermal conductance (CT) increased significantly with increasing body mass at all measurement T_a_s (*p* < 0.0001; Table 1). Further, CT followed a similar trend to T_b_rmr and RMR, such that at the lower T_a_s of 10 and 20°C the CT values were nearly identical in the A and C lines (*p* > 0.28; Table 1). At measurement T_a_s between 25 and 31°C the CT of the A lines was ≈6% higher than that measured in the C lines. However, this was reversed at 34°C, such that the CT of control voles was 3% higher than that of the selected voles. While these results were not significant, the repeated measures analysis performed for combined T_a_s (25, 28, 31, and 34°C) revealed that CT, averaged across the four temperatures, was nearly significantly higher in the A than in the C lines [A lines = 0.15 ± 0.003 mLO_2_/(min × °C); C lines = 0.14 ± 0.002 mLO_2_/(min × °C); *p* = 0.07]. As expected, the CT of the lines combined increased significantly with increasing T_a_ (25°C = 0.09±0.001 mLO_2_/(min × °C); 28°C = 0.11±0.002 mLO_2_/(min × °C); 31°C = 0.16±0.003 mLO_2_/(min × °C); 34°C = 0.24±0.005 mLO_2_/(min × °C); *p* = 0.04). Further, similarly to RMR, the interaction between the effect of Selection and T_a_ was not significant for CT (*p* = 0.19).

The analysis of the stage-regression model applied to characterize the thermoregulatory curve showed that the best fit model according to the AIC Fit statistic (Table 2, model 8) had a common LCT (26.1 ± 0.3°C), which did not depend significantly on either body mass or selection direction. The rate of metabolism above LCT (RMR_TNZ_), adjusted for the effect of body mass, was 0.11 ± 0.03 mlO_2_/min higher in the A than in the C lines (χ^2^ = 13.2, *p* < 0.001). The slope of the increase of RMR below LCT (*C*_*t*_), which can be treated as another characteristic of thermal conductance, increased with body mass (χ^2^ = 4.8, *p* = 0.028), and was significantly lower in the A than in the C lines [for a vole with a mean mass of 25 g: A lines: 0.078 mLO_2_/(min × °C), C lines: 0.081 mLO_2_/(min × °C), difference: 0.006 ± 0.003 mLO_2_/(min × °C); χ^2^ = 5.0, *p* = 0.025]. Thus, the thermoregulatory curve lines for the A and C voles meet at 7.5°C (Figure 2B). However, as RMR was not measured at even lower temperatures, the results do not allow us to resolve whether the lines intersect or converge. Below the LCT, RMR increased so that for the A lines at 20°C it was about 1.4-fold higher, and at 10°C was 2-fold higher, than RMR in the TNZ. As RMR in the TNZ was lower for the C lines, these differences were greater, such that RMR at 20°C was 1.5-fold higher, and at 10°C was 2.2-fold higher, than RMR in the TNZ.

**Table 2:** Summary of Fit statistics for nonlinear, stage-regression models (implemented in SAS NLMIXED procedure), applied to determine how selection affected the main characteristics of the thermoregulatory curve in bank voles.

	**Model specification (parameters included)**				**Fit statistics and criteria**			
	**Model number**	**N fixed parameters**	**Mass-slope coefficients**	**Different intercepts**	**−2 Log Likelihood**	**AIC**	**AICC**	**BIC**
Heterogeneous mass-slopes	1	12	*b_*m*_, c_*m*_, t_*m*_* different (selection-specific)	*b_0_, ct_0_, lc_0_*	−338.8	−310.8	−309.8	−269.6
Homogeneous mass-slopes (*b_*m*_, c_*m*_, t_*m*_* common in all further models)								
All mass-slope coefficients included	2	9	*b_*m*_, c_*m*_, t_*m*_*	*b_0_, c_0_, t_0_*	−331.2	−309.2	−308.6	−276.9
	3	8	*b_*m*_, c_*m*_, t_*m*_*	*c_0_, t_0_*	−321.0	−301.0	−300.5	−271.6
	4	8	*b_*m*_, c_*m*_, t_*m*_*	*b_0_, t_0_*	−328.8	−308.8	−308.3	−279.4
	5	8	*b_*m*_, c_*m*_, t_*m*_*	*b_0_, c_0_*,	−331.1	−311.1	−310.6	−281.7
Homogeneous slopes, but CT or LCT mass-slope coefficients excluded (mass-independence of the trait assumed)	6	8	*b_*m*_, t_*m*_*	*b_0_, c_0_, t_0_*	−328.7	−308.7	−308.2	−279.3
	7	8	*b_*m*_, c_*m*_*	*b_0_, c_0_, t_0_*	−331.3	−311.3	−310.8	−281.9
	**8**	**7**	***b**_*m*_**, c**_*m*_*	***b**_0_**, c**_0_,*	–**331.1**	–**313.1**	–**312.7**	–**286.7**
	9	6	*b_*m*_, c_*m*_*	*b_0_*	−326.1	−310.1	−309.8	−286.6
	10	6	*b_*m*_, c_*m*_*	*c_0_*	−317.9	−301.9	−301.6	−278.4
	11	6	*b_*m*_*,	*b_0_, c_0_*	−326.3	−310.3	−309.9	−286.7

*The three main parameters were entered in the model as linear functions of body mass (M_b_), with an intercept and mass-slope coefficients (“0” and “m” subscripts, respectively): the lower critical temperature (LCT = t_0_ + t_m_ × M_b_), resting metabolic rate above LCT (RMR_TNZ_ = b_0_ + b_m_ × M_b_), and thermal conductance (C_t_ = c_0_ + c_m_ × M_b_). The intercepts and mass-slope parameters were specified either as common or different for the A and C lines. Thus, the full model had 12 fixed parameters (plus two random effects, not shown), which were then step-wise reduced. The common intercept coefficients were always retained in the model, and therefore are not shown in the table. A lower value of the Fit criteria indicates a better fit of the model, and the best-fit model is highlighted in bold*.

The maximum thermogenesis ($\dot{\text{V}}$O_2_cold) of voles from the A lines was 10% higher than that measured in voles from the C lines (*p* < 0.0001; Table 1). However, the $\dot{\text{V}}$O_2_cold measured during the maximum thermogenesis trials did not differ between the A and C lines (*p* = 0.70; Table 1). An extrapolation of the thermoregulatory curve toward low temperatures and $\dot{\text{V}}$O_2_cold values allows an estimation of the lower lethal temperature (LLT), at which the thermoregulatory demand meets the ceiling of maximum thermogenesis. Although the slope of the curve was lower for the A lines, the difference may not hold at lower temperatures. Therefore, for the extrapolation below 7.5°C (where the lines meet) we used a common slope of 0.058 mLO_2_/(min × °C). The LLT calculated in this way for a 25 g vole was 8.4°C lower in the A (−28.6°C) than in the C lines (−20.2°C).

## Discussion

Our study provides data that fill in the gaps in our knowledge on how selecting for high-activity related aerobic metabolism can also result in correlated changes in thermal physiological traits. Specifically, as predicted by our previous studies on BMR (Sadowska et al., 2015), RMR within the TNZ was higher in selected (A) lines in comparison to control (C) lines. Resting metabolism is correlated with many life history traits, such as growth, survival, or reproductive output, which suggests that the fitness of an individual can be associated with RMR (Boratynski and Koteja, 2009, 2010; Burton et al., 2011). Thus, the increase in RMR in the TNZ in the voles from the A lines suggests that the evolution of increased aerobic exercise capacity leads to many other correlated changes. In addition, the T_b_ of selected voles in the TNZ was ≈0.3°C higher in comparison to control voles, and the difference increased to ≈1.5°C at a T_a_ of 34°C, revealing that the increased heat production resulting from higher RMR was not completely balanced by increased heat dissipation. While there are advantages for animals to have a high BMR or RMR, such as increased maximal MR for longer activity periods, there are also advantages to having a low BMR or RMR (Larivée et al., 2010). For example, individuals with a low BMR or RMR do not have to eat as much (Dheyongera et al., 2016); therefore their foraging requirements are reduced along with exposure to predators (Larivée et al., 2010). Importantly, while endotherms can maintain high metabolism and a stable T_b_ to remain active over a range of T_a_, this comes at a cost.

Selection for high-activity related metabolism could influence RMR at T_a_ below the LCT in several ways (Figure 1). The results of the stage-regression analysis supported a model in which the LCT does not differ between the A and C lines, and the slope of the thermoregulatory curve is lower in A lines, so that the lines meet at T_a_ = 7.5°C (Table 2, Figure 2B). Because we do not have results for T_a_s below 10°C or between 10 and 20°C, we could not attempt to fit a more complex model, which would allow to determine whether the thermoregulatory curves for the A and C lines converge at low T_a_, or if indeed they intersect, so that below ≈ 7°C RMR would become lower in the A than in the C lines. However, as the thermal conductance (CT), calculated from individual values of RMR and T_b_ measured at T_a_ = 10°C, does not differ significantly between the lines, and the CT value is actually even slightly higher in the A than in the C lines (Table 1), we can predict that the lines actually converge, rather than intersect. In addition to the similar RMR in the A and C lines measured at the moderately low temperatures, there were also no significant differences in T_b_. This result suggests that RMR and T_b_ at cold T_a_ are conserved regardless of selection for higher MR during activity, possibly to reduce the amount of energy needed to maintain normothermia below the LCT.

Importantly, while many endotherms employ physiological mechanisms to deal with cold temperatures, such as non-shivering thermogenesis (NST) and torpor, such tools are not employed by all species (Jackson et al., 2001; Ruf and Geiser, 2015; Stawski et al., 2015a). Interestingly, NST capacity measured in bank voles from the same generations as those in the current study did not differ between the A and C lines (Stawski et al., 2015a). Therefore, in bank voles NST capacity does not appear to be genetically correlated to activity-related metabolism and is likely a plastic trait, which would be advantageous in predictable environments such as those that bank voles occupy (Stawski et al., 2015a). By conserving RMR and NST capacity at low T_a_ regardless of selection pressures acting on activity-related aerobic metabolism, bank voles with high BMR can also survive these cold temperatures by not increasing the amount of energy used to remain normothermic (however, their overall energy needs may be increased if they maintain a higher locomotor activity). Importantly, as RMR in the TNZ is higher for the A lines in comparison to the C lines, the selected voles do not need to increase their RMR as much at T_a_ below the LCT. This smaller increase in energy expenditure as the temperature drops was likely important during the evolution of endothermy and also currently in terms of adapting rapidly to colder weather. Additionally, our data also revealed that voles from the A lines have a 10% higher thermogenic capacity (Table 1). Consequently, as RMR and CT below the LCT practically do not differ between the selection directions, the selected voles presumably have a 7°C lower LLT than voles from the C lines, i.e., have a higher capacity to withstand very cold T_a_.

Hotter temperatures can be more difficult for endotherms to cope with than cold. Endotherms in particular can overheat rapidly and this can lead to organ failure and death. Therefore, for T_a_ above the UCT endotherms need to dissipate heat and often do so by increasing evaporative water loss and MR (Pis, 2010; Rezende and Bacigalupe, 2015). Due to the higher RMR and T_b_ of the selected voles in the TNZ, we predicted that the slope of the line would be steeper above the UCT in comparison to control lines. While we were unable to estimate the UCT based on our data, the RMR and T_b_ of some voles already showed an increase at the measurement T_a_ of 31°C, and an even greater increase at 34°C. Therefore, while there appears to be considerable individual variation, we hypothesize that the UCT of voles is around 31°C. Importantly, the T_b_ of voles from the A lines at 34°C was much higher in comparison to the C lines. This result suggests that selection for high activity-related metabolism can reduce the capacity of effective thermoregulation at high T_a_, and therefore decrease the upper lethal temperature.

The results of our current and previous studies show that selection for increased aerobic capacity during activity leads to many other correlated changes, such as a shift in the thermoregulatory curve as shown here. Specifically, the selection for high aerobic exercise performance, even though operating under thermally neutral conditions, has resulted in the evolution of increased cold tolerance, which, under natural conditions, could allow voles to inhabit colder environments. Conversely, this selection has also resulted in voles overheating at high T_a_, which may lead to difficulties in a warming climate. Bank voles offer an ideal model to analyse metabolic adjustments to differing climates as they have a wide distribution throughout Europe, extending in the North all the way into the Arctic Circle and down to the Mediterranean in the South (Raczyński, 1983). In the West they are found in Ireland and in the East they inhabit a large portion of Russia (Raczyński, 1983). Throughout this large range bank voles experience a variety of climates, suggesting they may display physiological flexibility to enable adaptation to differing weather patterns. Yet, surprisingly, the possibility of geographical variation of their metabolic traits has not been explored extensively and we are only aware of one study (Aalto et al., 1993). This research revealed that bank voles do not appear to display evident variation in BMR across a wide geographical range, from Northern Finland to the Balkan Peninsulas. The authors suggested that throughout this wide climatic range voles can select similar microclimates (Aalto et al., 1993). However, the methodology of this study was not perfect, because the measurements were conducted neither on voles' immediately after capture nor on ones maintained under common-garden conditions. Thus, the data do not represent effects of current local conditions, and do not represent genetically-based differences, either. Therefore, it would be beneficial to repeat such an experiment with a more robust experimental design, and compare the outcome with that of our selection experiment. To conclude, the results of the current thermoregulatory curve experiment and the results from the whole experimental evolutionary model research support the assumptions of the aerobic capacity model of the evolution of endothermy.

## Author contributions

All authors designed the study, analyzed the results, worked on the manuscript and gave approval for publication. CS and ES performed the experiments.

### Conflict of interest statement

The authors declare that the research was conducted in the absence of any commercial or financial relationships that could be construed as a potential conflict of interest.
