# Supplementary material for: A Shift in the Thermoregulatory Curve as a Result of Selection for High Activity-Related Aerobic Metabolism
Source: Front Physiol. 2017 Dec 18;8:1070. doi: 10.3389/fphys.2017.01070 (PMC5741638; doi:10.3389/fphys.2017.01070)
Supplement: Supplementary file 1 [file DataSheet1.PDF]

Supplementary Material 1

A shift in the thermoregulatory curve as a result of selection for high activity-related aerobic metabolism

Clare Stawski*, **Paweł Koteja and Edyta T. Sadowska**

***Correspondence:** clare.stawski@ntnu.no

Supplementary Results and Table

One of the reviewers of our manuscript expressed concerns that during the swimming trials at 38°C, performed as a part of the selection procedure (see Methods in the main part of the manuscript), the voles may be subject to overheating, which would have implications for predictions concerning the effect of selection on the ability of voles to dissipate excess heat at high air temperatures. However, from previous observations we knew that this is not the case, and, conversely, that performing the trials at a lower water temperature leads to cold stress and hypothermia. We use this opportunity to present the results of a simple experiment that illustrate this point.

We present data for body temperature of bank voles measured after swimming and running trials. The exercise trials were performed in the same way as the measurements of the maximum rates of oxygen consumption achieved during swimming or running within our experimental evolution research program on bank voles.

Swimming trials lasted 15 minutes and were performed in a chamber partly filled with water with a drop of a shampoo for dogs (to ensure complete soaking of fur). The water temperature was either +38°C as in the regular selection protocol applied to lines of bank voles selected for high aerobic metabolism, or decreased to +35°C.

Running trials were performed in a respirometric treadmill for rodents (BTU-100-10-M, Bio-Sys-Tech, Bialystok, Poland) at 20°C. The animals were forced to run with mild electric shocks (0.5 mA) generated by bars located behind the moving belt. The fur of the animal's abdomen and hind legs was moistened with water with a drop of dog shampoo, to increase electric conductivity (without this procedure the animals ignored the electric shocks). The treadmill started to move at 6 m min^{-1} two minutes after starting the trial and the speed was increased by 6 m min^{-1} in every minute. The test lasted till exhaustion, i.e. until the animal was unable to keep pace with the moving belt (typically 10 minutes).

Rectal body temperature was measured just before and immediately after the performance trials using Oakton Temp-300 (Thermocouple Datalogging Thermometer, OAKTON Instruments, USA).

The results (Supplementary Table 1) show clearly that swimming at +38°C, i.e. at a temperature close to body temperature, does not inflict problems with offloading extra heat (no increase of body temperature is observed). On the other hand, running in air at room temperature results in an increased body temperature. More importantly, decreasing the water temperature during the swimming trials by just 3°C (to 35°C) results in a marked hypothermia. Thus, if the swimming tests used in the selection

protocol were performed in a temperature even slightly lower than 38°C, the voles would be partly selected for cold-induced capability (thermogenesis), rather than – as intended – for purely locomotor-related metabolism.

Supplementary Table 1: Rectal body temperature (T_b; mean ± standard error) of bank voles, measured before and after exercise performance trials applying different measurement protocols and different ambient temperatures (T_a).

Trial	T_a (°C)	N	Body mass (g)	T_b before (°C)	T_b after (°C)
swimming	35	6	23.02±3.39	38.47±0.59	36.25±0.37
swimming	38	6	23.35±3.74	38.62±0.78	38.68±0.39
forced treadmill running	20	6	22.27±3.11	38.65±0.62	40.72±0.50
