# Supplementary material for: A Shift in the Thermoregulatory Curve as a Result of Selection for High Activity-Related Aerobic Metabolism
Source: Front Physiol. 2017 Dec 18;8:1070. doi: 10.3389/fphys.2017.01070 (PMC5741638; doi:10.3389/fphys.2017.01070)
Supplement: Supplementary file 2 [file DataSheet2.pdf]

Supplementary Material 2

A shift in the thermoregulatory curve as a result of selection for high activity-related aerobic metabolism

Clare Stawski[*]**, Paweł Koteja and Edyta T. Sadowska**

***Correspondence:** clare.stawski@ntnu.no

Supplementary Statistical Data

In this file we provide the output from SAS (v. 9.3) for the statistical analyses conducted in our manuscript.

The following variables were included in the analyses:

- Selection direction (T) = selected (A) and control (C)
- Sex (S) = male (1) and female (0)
- Lines (L) = selected lines (A1, A2, A3, A4) and control lines (C1, C2, C3, C4)
- Generation (Gen) = 13th (Y) and 14th (Z) generations
- Timing = Morning or Afternoon
- Body mass (MB0) = body mass of vole at the start of a trial
- Age = age of voles on the day of a trial

Table of Contents ....page number

Part 1. Analyses of thermoregulatory traits at particular temperatures (10-34°C) 4

1.1. $T_b$mean: the mean T_b from 30-minutes of data that were recorded 4.5 to 5-hours after putting animal in the chamber

1.1.1. $T_b$mean at 10°C 5

1.1.2. $T_b$mean at 20°C 8

1.1.3. $T_b$mean at 25°C 11

1.1.4. $T_b$mean at 28°C 14

1.1.5. $T_b$mean at 31°C 17

1.1.6. $T_b$mean at 34°C 20

1.2. $T_b$rmr: $T_b$rmr is the T_b recorded at the time of the lowest RMR measurement

1.2.1. $T_b$rmr at 10°C 23

1.2.2. $T_b$rmr at 20°C 26

1.2.3. $T_b$rmr at 25°C 29

1.2.4. $T_b$rmr at 28°C 32

1.2.5. $T_b$rmr at 31°C 35

1.2.6. $T_b$rmr at 34°C 38

1.3. RMR: lowest resting metabolic rate

1.3.1. RMR at 10°C 41

1.3.2. RMR at 20°C 44

1.3.3. RMR at 25°C 47

1.3.4. RMR at 28°C 50

1.3.5. RMR at 31°C 53

1.3.6. RMR at 34°C 56

1.4. CT: thermal conductance (estimated for a particular temperature)

1.4.1. CT at 10°C 59

1.4.2. CT at 20°C 62

1.4.3. CT at 25°C 65

1.4.4. CT at 28°C 68

1.4.5. CT at 31°C 71

1.4.6. CT at 34°C 74

Part 2. Analyses of traits associated with therogenic capacity 77

2.1. $T_b$cold: T_b after maximum thermogenesis experiment 78

2.2. $\dot{V}O_2$cold: maximal MR during thermogenesis experiment 81

Part 3. Repeated measures analyses for $T_b$rmr, RMR and CT at temperatures around the thermoneutral zone 84

3.1. $T_b$rmr 85

3.2. RMR 91

3.3. CT 97

Part 4. Stage-regression, mixed nonlinear model for fitting parameters of the thermoregulatory curve 103

4.1. Main characteristics of the thermoregulatory curve 104

Part 1. Analyses of thermoregulatory traits at particular temperatures (10-34°C)

Mixed Model analyses performed for each of the variables at each of the measurement temperatures. To compare RMR and T_b of voles from selected and control lines at each of the measurement T_as and also for maximum thermogenic capacity we use the Mixed Procedure (with REML method) to estimate cross-nested Mixed ANCOVA model, with Selection (selected *vs.* control) as the main, top-level fixed factor, replicated Lines as random effect nested within Selection. Further, we also included the following variables in all analyses as additional fixed covariates or cofactors: Sex, Generation, Age, Body Mass, Timing (i.e. Morning or Afternoon). The model included also a fixed interaction of Selection*Sex and the random interaction of Sex*Line. Values that were obtained from active individuals were omitted from the analyses. Additionally, studentized residuals were obtained and any residuals below -3 or above 3 were considered outliers and removed from the data set. Adjusted least square means (LSM) were calculated using a fixed body mass (25g) and age (140 days).

1.1. $T_b$mean: the mean T_b from 30-minutes of data that were recorded 4.5 to 5-hours after putting animal in the chamber

1.1.1. Output from SAS (v. 9.3) Mixed Model analysis for $T_b$mean at 10°C.

Model Information	
Data Set	WORK.THERMLONG
Dependent Variable	Tb_mean
Covariance Structure	Variance Components
Estimation Method	REML
Residual Variance Method	Profile
Fixed Effects SE Method	Model-Based
Degrees of Freedom Method	Satterthwaite

Class Level Information		
Class	Levels	Values
T	2	A C
S	2	0 1
L	8	A1 A2 A3 A4 C1 C2 C3 C4
Gen	2	Y Z
Timing	2	Afternoon Morning

Dimensions	
Covariance Parameters	3
Columns in X	15
Columns in Z	24
Subjects	1
Max Obs per Subject	106

Number of Observations	
Number of Observations Read	106
Number of Observations Used	106
Number of Observations Not Used	0

Covariance Parameter Estimates							
Cov Parm	Estimate	Standard Error	Z Value	Pr > Z	Alpha	Lower	Upper
L(T)	0.02027	0.04579	0.44	0.3290	0.05	0.002661	906347
S*L(T)	0.03667	0.04740	0.77	0.2196	0.05	0.007920	12.5818
Residual	0.2785	0.04253	6.55	<.0001	0.05	0.2109	0.3850

Fit Statistics	
**-2 Res Log Likelihood**	199.5
**AIC (Smaller is Better)**	205.5
**AICC (Smaller is Better)**	205.8
**BIC (Smaller is Better)**	205.8

Solution for Fixed Effects

Effect	T	Gen	Timing	S	Estimate	Standard Error	DF	t Value	Pr > |t|	Alpha	Lower	Upper
**Intercept**					37.9479	0.5487	84.6	69.16	<.0001	0.05	36.8569	39.0389
**T**	A				-0.3898	0.2344	12.2	-1.66	0.1217	0.05	-0.8993	0.1198
**T**	C				0	.	.	.	.	.	.	.
**S**				0	0.1242	0.2075	6.64	0.60	0.5692	0.05	-0.3718	0.6203
**S**				1	0	.	.	.	.	.	.	.
**T*S**	A			0	0.4523	0.2819	5.7	1.60	0.1623	0.05	-0.2464	1.1510
**T*S**	A			1	0	.	.	.	.	.	.	.
**T*S**	C			0	0	.	.	.	.	.	.	.
**T*S**	C			1	0	.	.	.	.	.	.	.
**Gen**		Y			-0.04485	0.1646	88.7	-0.27	0.7860	0.05	-0.3720	0.2823
**Gen**		Z			0	.	.	.	.	.	.	.
**Timing**			Afternoon		0.01767	0.1100	94.6	0.16	0.8728	0.05	-0.2008	0.2361
**Timing**			Morning		0	.	.	.	.	.	.	.
**MB0**					0.006310	0.02003	92.9	0.32	0.7534	0.05	-0.03346	0.04608
**Age**					0.002564	0.002660	88.6	0.96	0.3377	0.05	-0.00272	0.007850

Type 3 Tests of Fixed Effects

Effect	Num DF	Den DF	F Value	Pr > F
**T**	1	6.16	0.79	0.4071
**S**	1	8.24	5.08	0.0534
**T*S**	1	5.7	2.57	0.1623
**Gen**	1	88.7	0.07	0.7860
**Timing**	1	94.6	0.03	0.8728
**MB0**	1	92.9	0.10	0.7534
**Age**	1	88.6	0.93	0.3377

Least Squares Means												
**Effect**	**T**	**Gen**	**Timing**	**S**	**Estimate**	**Standard Error**	**DF**	**t Value**	**Pr > |t|**	**Alpha**	**Lower**	**Upper**
**T**	A				38.3495	0.1261	5.54	304.02	<.0001	0.05	38.0346	38.6645
**T**	C				38.5132	0.1268	5.62	303.68	<.0001	0.05	38.1977	38.8287
**S**				0	38.6065	0.1174	12.2	328.87	<.0001	0.05	38.3513	38.8618
**S**				1	38.2562	0.1157	11.7	330.57	<.0001	0.05	38.0034	38.5090
**T*S**	A			0	38.6378	0.1592	10.8	242.69	<.0001	0.05	38.2865	38.9891
**T*S**	A			1	38.0613	0.1703	13.4	223.46	<.0001	0.05	37.6944	38.4282
**T*S**	C			0	38.5753	0.1687	12.7	228.72	<.0001	0.05	38.2102	38.9404
**T*S**	C			1	38.4510	0.1589	10.6	242.00	<.0001	0.05	38.0998	38.8023
**Gen**		Y			38.4089	0.1242	19.9	309.37	<.0001	0.05	38.1498	38.6680
**Gen**		Z			38.4538	0.1150	15.1	334.46	<.0001	0.05	38.2088	38.6987
**Timing**			Afternoon		38.4402	0.1037	9.96	370.67	<.0001	0.05	38.2090	38.6714
**Timing**			Morning		38.4225	0.1019	9.41	377.14	<.0001	0.05	38.1936	38.6514

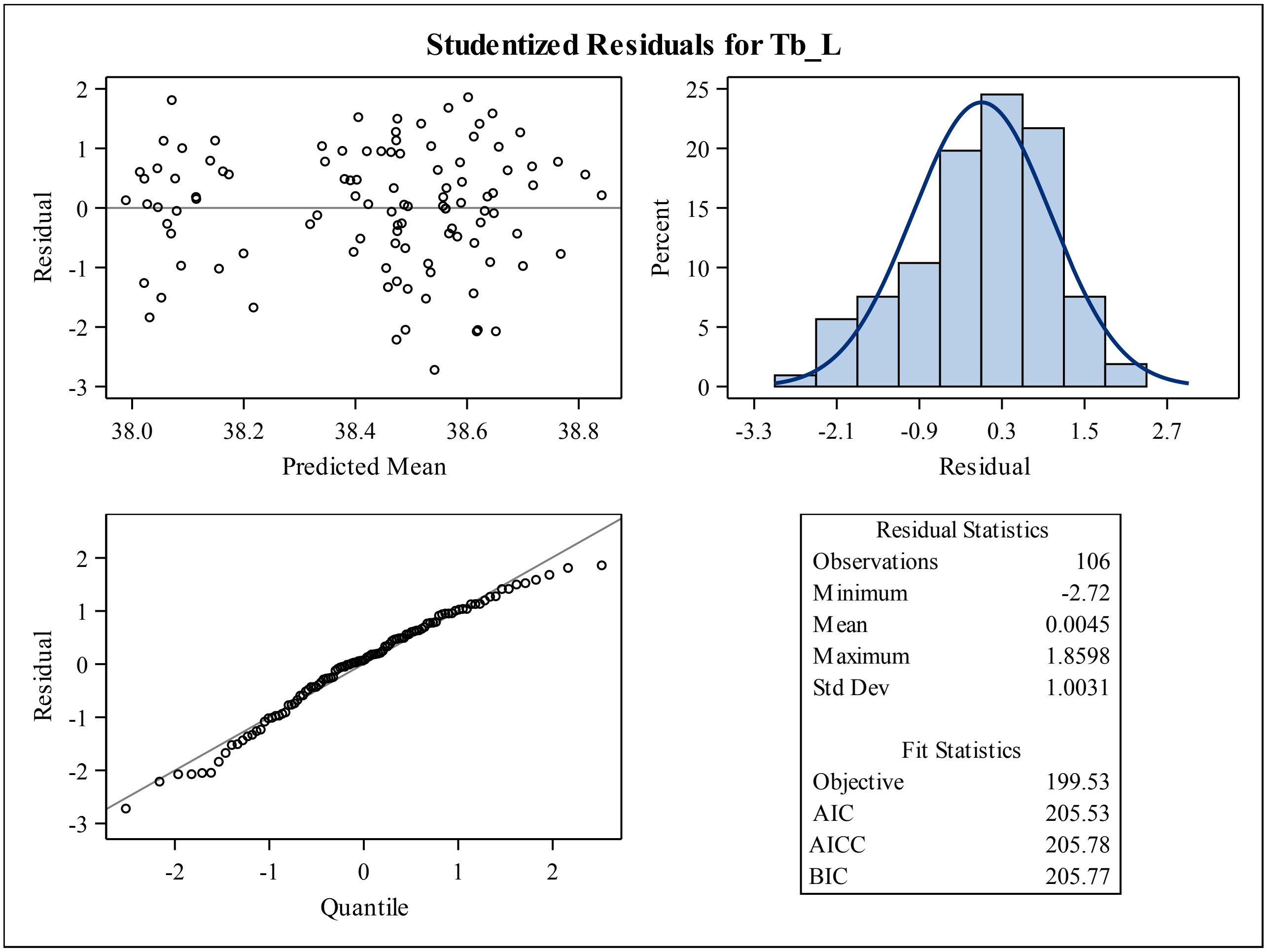

1.1.2. Output from SAS (v. 9.3) Mixed Model analysis for $T_b$mean at 20°C.

Model Information	
Data Set	WORK.THERMLONG
Dependent Variable	Tb_mean
Covariance Structure	Variance Components
Estimation Method	REML
Residual Variance Method	Profile
Fixed Effects SE Method	Model-Based
Degrees of Freedom Method	Satterthwaite

Class Level Information		
Class	Levels	Values
T	2	A C
S	2	0 1
L	8	A1 A2 A3 A4 C1 C2 C3 C4
Gen	2	Y Z
Timing	2	Afternoon Morning

Dimensions	
Covariance Parameters	3
Columns in X	15
Columns in Z	24
Subjects	1
Max Obs per Subject	129

Number of Observations	
Number of Observations Read	129
Number of Observations Used	129
Number of Observations Not Used	0

Covariance Parameter Estimates							
Cov Parm	Estimate	Standard Error	Z Value	Pr > Z	Alpha	Lower	Upper
L(T)	0	.	.	.	.	.	.
S*L(T)	0	.	.	.	.	.	.
Residual	0.3373	0.04336	7.78	<.0001	0.05	0.2661	0.4414

Fit Statistics	
-2 Res Log Likelihood	250.8
AIC (Smaller is Better)	252.8
AICC (Smaller is Better)	252.8
BIC (Smaller is Better)	252.9

Solution for Fixed Effects

Effect	T	Gen	Timing	S	Estimate	Standard Error	DF	t Value	Pr > |t|	Alpha	Lower	Upper
Intercept					38.5213	0.4678	121	82.35	<.0001	0.05	37.5952	39.4474
T	A				0.09699	0.1564	121	0.62	0.5365	0.05	-0.2127	0.4067
T	C				0	.	.	.	.	.	.	.
S				0	-0.2466	0.1538	121	-1.60	0.1113	0.05	-0.5510	0.05777
S				1	0	.	.	.	.	.	.	.
T*S	A			0	0.3444	0.2055	121	1.68	0.0963	0.05	-0.06235	0.7512
T*S	A			1	0	.	.	.	.	.	.	.
T*S	C			0	0	.	.	.	.	.	.	.
T*S	C			1	0	.	.	.	.	.	.	.
Gen		Y			-0.3388	0.1446	121	-2.34	0.0208	0.05	-0.6251	-0.05241
Gen		Z			0	.	.	.	.	.	.	.
Timing			Afternoon		0.1649	0.1033	121	1.60	0.1133	0.05	-0.03974	0.3694
Timing			Morning		0	.	.	.	.	.	.	.
MB0					-0.04145	0.01723	121	-2.41	0.0176	0.05	-0.07556	-0.00735
Age					0.005947	0.002337	121	2.54	0.0122	0.05	0.001320	0.01057

Type 3 Tests of Fixed Effects

Effect	Num DF	Den DF	F Value	Pr > F
T	1	121	5.44	0.0213
S	1	121	0.40	0.5261
T*S	1	121	2.81	0.0963
Gen	1	121	5.49	0.0208
Timing	1	121	2.54	0.1133
MB0	1	121	5.79	0.0176
Age	1	121	6.48	0.0122

Least Squares Means												
**Effect**	**T**	**Gen**	**Timing**	**S**	**Estimate**	**Standard Error**	**DF**	**t Value**	**Pr > |t|**	**Alpha**	**Lower**	**Upper**
**T**	A				38.3764	0.07613	121	504.10	<.0001	0.05	38.2257	38.5272
**T**	C				38.1072	0.07857	121	485.00	<.0001	0.05	37.9517	38.2628
**S**				0	38.2046	0.07848	121	486.81	<.0001	0.05	38.0493	38.3600
**S**				1	38.2790	0.07745	121	494.22	<.0001	0.05	38.1257	38.4324
**T*S**	A			0	38.4253	0.1043	121	368.32	<.0001	0.05	38.2188	38.6319
**T*S**	A			1	38.3275	0.1146	121	334.32	<.0001	0.05	38.1006	38.5545
**T*S**	C			0	37.9839	0.1143	121	332.18	<.0001	0.05	37.7575	38.2103
**T*S**	C			1	38.2306	0.1053	121	362.99	<.0001	0.05	38.0220	38.4391
**Gen**		Y			38.0725	0.08523	121	446.71	<.0001	0.05	37.9037	38.2412
**Gen**		Z			38.4112	0.09221	121	416.57	<.0001	0.05	38.2287	38.5938
**Timing**			Afternoon		38.3243	0.07332	121	522.70	<.0001	0.05	38.1791	38.4694
**Timing**			Morning		38.1594	0.07260	121	525.63	<.0001	0.05	38.0157	38.3031

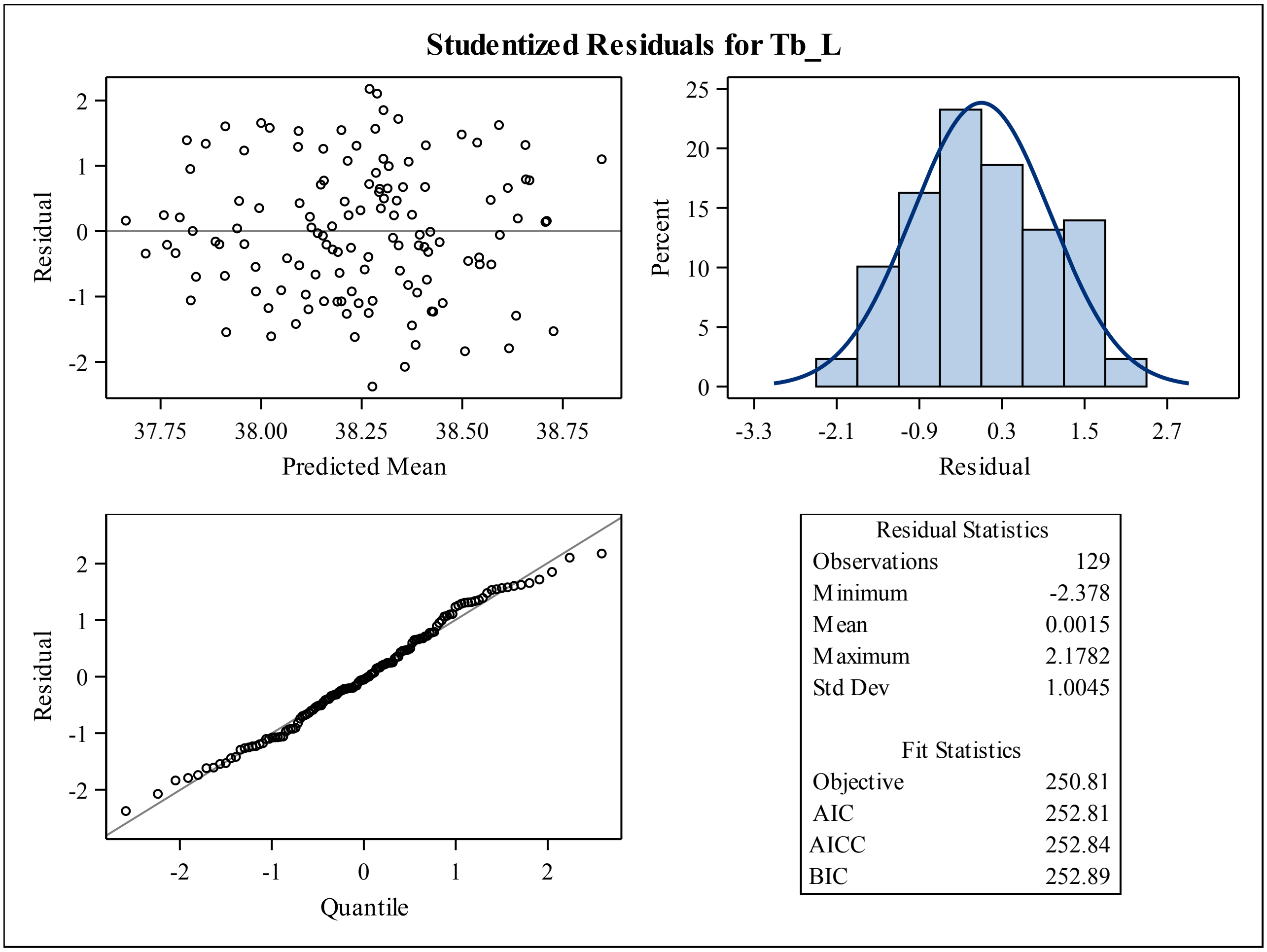

1.1.3. Output from SAS (v. 9.3) Mixed Model analysis for $T_b$mean at 25°C.

Model Information	
Data Set	WORK.THERMLONG
Dependent Variable	Tb_mean
Covariance Structure	Variance Components
Estimation Method	REML
Residual Variance Method	Profile
Fixed Effects SE Method	Model-Based
Degrees of Freedom Method	Satterthwaite

Class Level Information		
Class	Levels	Values
T	2	A C
S	2	0 1
L	8	A1 A2 A3 A4 C1 C2 C3 C4
Gen	2	Y Z
Timing	2	Afternoon Morning

Dimensions	
Covariance Parameters	3
Columns in X	15
Columns in Z	24
Subjects	1
Max Obs per Subject	62

Number of Observations	
Number of Observations Read	62
Number of Observations Used	62
Number of Observations Not Used	0

Covariance Parameter Estimates

Cov Parm	Estimate	Standard Error	Z Value	Pr > Z	Alpha	Lower	Upper
L(T)	0	.	.	.	.	.	.
S*L(T)	0	.	.	.	.	.	.
Residual	0.3162	0.06086	5.20	<.0001	0.05	0.2241	0.4799

Fit Statistics	
**-2 Res Log Likelihood**	123.8
**AIC (Smaller is Better)**	125.8
**AICC (Smaller is Better)**	125.9
**BIC (Smaller is Better)**	125.9

Solution for Fixed Effects												
**Effect**	**T**	**Gen**	**Timing**	**S**	**Estimate**	**Standard Error**	**DF**	**t Value**	**Pr > |t|**	**Alpha**	**Lower**	**Upper**
**Intercept**					37.6748	0.6958	54	54.14	<.0001	0.05	36.2797	39.0699
**T**	A				0.1092	0.2108	54	0.52	0.6066	0.05	-0.3135	0.5319
**T**	C				0	.	.	.	.	.	.	.
**S**				0	0.2962	0.2183	54	1.36	0.1804	0.05	-0.1414	0.7338
**S**				1	0	.	.	.	.	.	.	.
**T*S**	A			0	-0.01622	0.2876	54	-0.06	0.9552	0.05	-0.5929	0.5604
**T*S**	A			1	0	.	.	.	.	.	.	.
**T*S**	C			0	0	.	.	.	.	.	.	.
**T*S**	C			1	0	.	.	.	.	.	.	.
**Gen**		Y			0.1563	0.1976	54	0.79	0.4325	0.05	-0.2399	0.5525
**Gen**		Z			0	.	.	.	.	.	.	.
**Timing**			Afternoon		0.001148	0.1463	54	0.01	0.9938	0.05	-0.2921	0.2944
**Timing**			Morning		0	.	.	.	.	.	.	.
**MB0**					0.03224	0.02924	54	1.10	0.2750	0.05	-0.02638	0.09086
**Age**					-0.00455	0.003335	54	-1.36	0.1783	0.05	-0.01124	0.002138

Type 3 Tests of Fixed Effects				
**Effect**	**Num DF**	**Den DF**	**F Value**	**Pr > F**
**T**	1	54	0.38	0.5384
**S**	1	54	3.14	0.0822
**T*S**	1	54	0.00	0.9552
**Gen**	1	54	0.63	0.4325
**Timing**	1	54	0.00	0.9938
**MB0**	1	54	1.22	0.2750
**Age**	1	54	1.86	0.1783

Least Squares Means												
**Effect**	**T**	**Gen**	**Timing**	**S**	**Estimate**	**Standard Error**	**DF**	**t Value**	**Pr > |t|**	**Alpha**	**Lower**	**Upper**
**T**	A				38.1720	0.1086	54	351.38	<.0001	0.05	37.9542	38.3898
**T**	C				38.0709	0.1090	54	349.14	<.0001	0.05	37.8522	38.2895
**S**				0	38.2655	0.1119	54	341.99	<.0001	0.05	38.0411	38.4898
**S**				1	37.9774	0.1052	54	360.85	<.0001	0.05	37.7664	38.1884
**T*S**	A			0	38.3119	0.1478	54	259.29	<.0001	0.05	38.0157	38.6082
**T*S**	A			1	38.0320	0.1584	54	240.08	<.0001	0.05	37.7144	38.3496
**T*S**	C			0	38.2190	0.1683	54	227.13	<.0001	0.05	37.8816	38.5563
**T*S**	C			1	37.9228	0.1389	54	273.07	<.0001	0.05	37.6443	38.2012
**Gen**		Y			38.1996	0.1214	54	314.56	<.0001	0.05	37.9561	38.4430
**Gen**		Z			38.0433	0.1231	54	309.15	<.0001	0.05	37.7965	38.2900
**Timing**			Afternoon		38.1220	0.10000	54	381.22	<.0001	0.05	37.9215	38.3225
**Timing**			Morning		38.1208	0.1052	54	362.40	<.0001	0.05	37.9099	38.3317

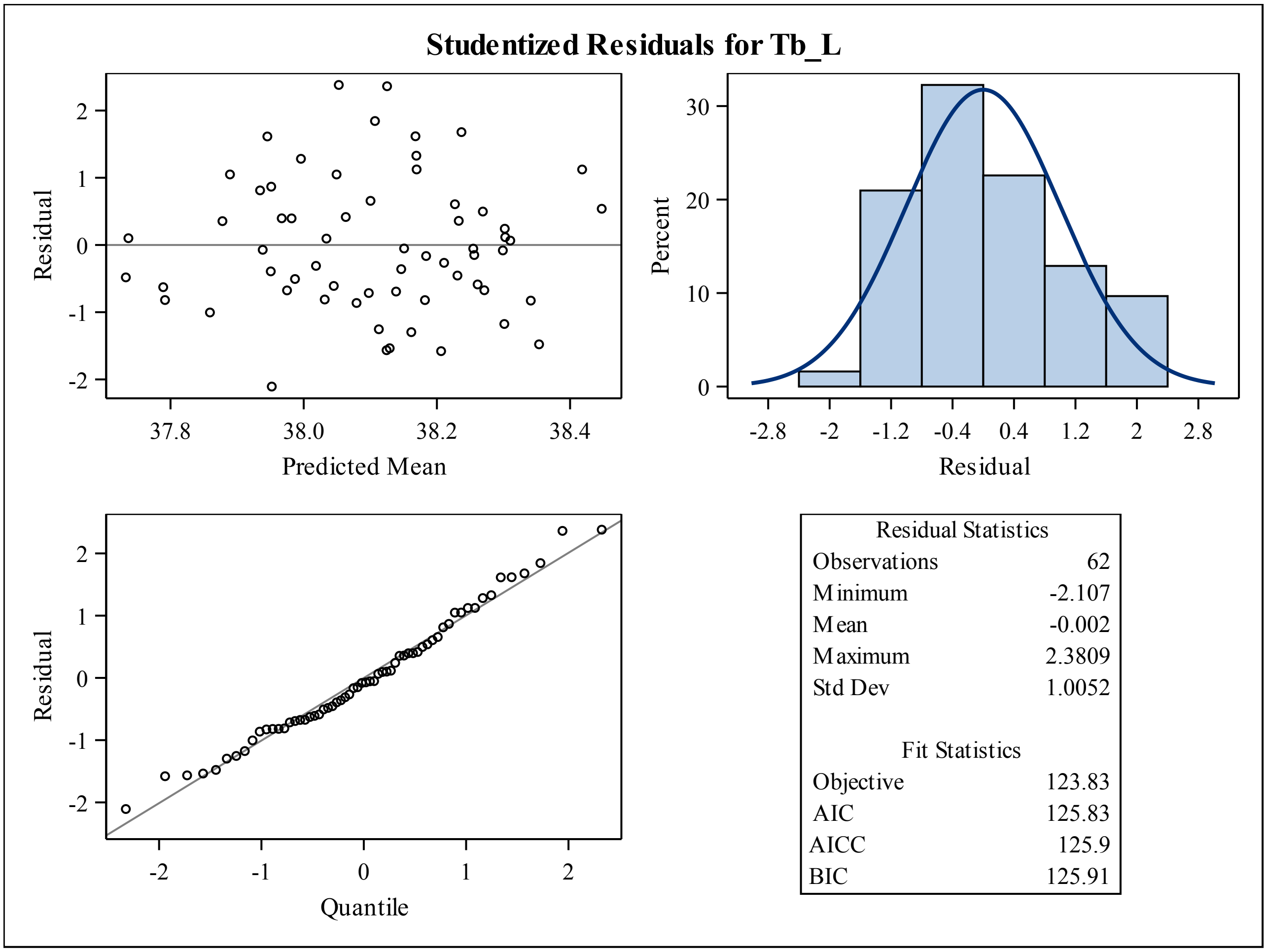

1.1.4. Output from SAS (v. 9.3) Mixed Model analysis for $T_b$mean at 28°C.

Model Information	
**Data Set**	WORK.THERMLONG
**Dependent Variable**	Tb_mean
**Covariance Structure**	Variance Components
**Estimation Method**	REML
**Residual Variance Method**	Profile
**Fixed Effects SE Method**	Model-Based
**Degrees of Freedom Method**	Satterthwaite

Class Level Information		
**Class**	**Levels**	**Values**
**T**	2	A C
**S**	2	0 1
**L**	8	A1 A2 A3 A4 C1 C2 C3 C4
**Gen**	2	Y Z
**Timing**	2	Afternoon Morning

Dimensions	
**Covariance Parameters**	3
**Columns in X**	15
**Columns in Z**	24
**Subjects**	1
**Max Obs per Subject**	60

Number of Observations	
**Number of Observations Read**	60
**Number of Observations Used**	60
**Number of Observations Not Used**	0

Covariance Parameter Estimates							
**Cov Parm**	**Estimate**	**Standard Error**	**Z Value**	**Pr > Z**	**Alpha**	**Lower**	**Upper**
**L(T)**	0.01319	0.03782	0.35	0.3636	0.05	0.001440	3.79E10
**S*L(T)**	0	.	.	.	.	.	.
**Residual**	0.2968	0.06283	4.72	<.0001	0.05	0.2039	0.4718

Fit Statistics	
**-2 Res Log Likelihood**	118.3
**AIC (Smaller is Better)**	122.3
**AICC (Smaller is Better)**	122.6
**BIC (Smaller is Better)**	122.5

Solution for Fixed Effects

Effect	T	Gen	Timing	S	Estimate	Standard Error	DF	t Value	Pr > |t|	Alpha	Lower	Upper
**Intercept**					38.4389	0.7267	50.2	52.90	<.0001	0.05	36.9795	39.8983
**T**	A				0.2776	0.2215	13.7	1.25	0.2309	0.05	-0.1982	0.7534
**T**	C				0	.	.	.	.	.	.	.
**S**				0	0.03768	0.2186	51	0.17	0.8638	0.05	-0.4013	0.4766
**S**				1	0	.	.	.	.	.	.	.
**T*S**	A			0	0.07616	0.2874	48.4	0.27	0.7921	0.05	-0.5015	0.6539
**T*S**	A			1	0	.	.	.	.	.	.	.
**T*S**	C			0	0	.	.	.	.	.	.	.
**T*S**	C			1	0	.	.	.	.	.	.	.
**Gen**		Y			0.1889	0.2063	47.7	0.92	0.3644	0.05	-0.2259	0.6037
**Gen**		Z			0	.	.	.	.	.	.	.
**Timing**			Afternoon		-0.09232	0.1463	49.3	-0.63	0.5310	0.05	-0.3863	0.2017
**Timing**			Morning		0	.	.	.	.	.	.	.
**MB0**					-0.00110	0.02933	48.9	-0.04	0.9701	0.05	-0.06005	0.05784
**Age**					-0.00380	0.003550	48.6	-1.07	0.2902	0.05	-0.01093	0.003339

Type 3 Tests of Fixed Effects

Effect	Num DF	Den DF	F Value	Pr > F
**T**	1	6.89	2.87	0.1348
**S**	1	51.6	0.22	0.6442
**T*S**	1	48.4	0.07	0.7921
**Gen**	1	47.7	0.84	0.3644
**Timing**	1	49.3	0.40	0.5310
**MB0**	1	48.9	0.00	0.9701
**Age**	1	48.6	1.14	0.2902

Least Squares Means												
**Effect**	**T**	**Gen**	**Timing**	**S**	**Estimate**	**Standard Error**	**DF**	**t Value**	**Pr > |t|**	**Alpha**	**Lower**	**Upper**
**T**	A				38.2625	0.1242	5.69	308.10	<.0001	0.05	37.9546	38.5705
**T**	C				37.9468	0.1240	5.39	306.14	<.0001	0.05	37.6350	38.2587
**S**				0	38.1426	0.1206	15.4	316.32	<.0001	0.05	37.8862	38.3989
**S**				1	38.0668	0.1104	12.9	344.96	<.0001	0.05	37.8282	38.3054
**T*S**	A			0	38.3195	0.1644	15	233.15	<.0001	0.05	37.9691	38.6698
**T*S**	A			1	38.2056	0.1648	15.9	231.76	<.0001	0.05	37.8559	38.5553
**T*S**	C			0	37.9657	0.1815	18.3	209.23	<.0001	0.05	37.5849	38.3465
**T*S**	C			1	37.9280	0.1473	10.8	257.44	<.0001	0.05	37.6031	38.2529
**Gen**		Y			38.1991	0.1337	23.5	285.65	<.0001	0.05	37.9228	38.4754
**Gen**		Z			38.0102	0.1296	23.2	293.22	<.0001	0.05	37.7422	38.2783
**Timing**			Afterno on		38.0585	0.1095	12.3	347.66	<.0001	0.05	37.8206	38.2964
**Timing**			Morning		38.1508	0.1102	13.3	346.28	<.0001	0.05	37.9134	38.3883

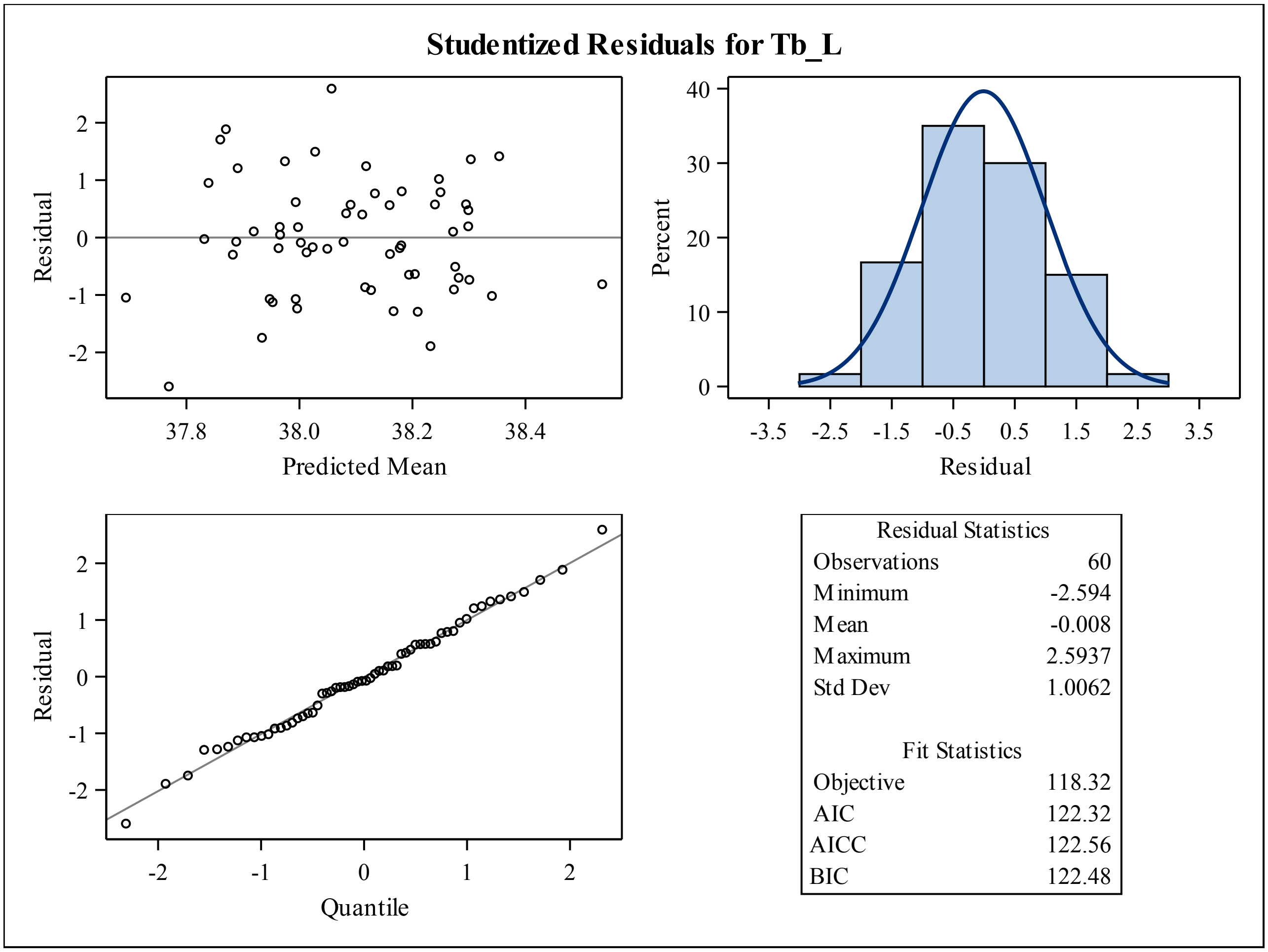

1.1.5. Output from SAS (v. 9.3) Mixed Model analysis for $T_b$mean at 31°C.

Model Information	
**Data Set**	WORK.THERMLONG
**Dependent Variable**	Tb_mean
**Covariance Structure**	Variance Components
**Estimation Method**	REML
**Residual Variance Method**	Profile
**Fixed Effects SE Method**	Model-Based
**Degrees of Freedom Method**	Satterthwaite

Class Level Information		
**Class**	**Levels**	**Values**
**T**	2	A C
**S**	2	0 1
**L**	8	A1 A2 A3 A4 C1 C2 C3 C4
**Gen**	2	Y Z
**Timing**	2	Afternoon Morning

Dimensions	
**Covariance Parameters**	3
**Columns in X**	15
**Columns in Z**	24
**Subjects**	1
**Max Obs per Subject**	62

Number of Observations	
**Number of Observations Read**	62
**Number of Observations Used**	62
**Number of Observations Not Used**	0

Covariance Parameter Estimates							
**Cov Parm**	**Estimate**	**Standard Error**	**Z Value**	**Pr > Z**	**Alpha**	**Lower**	**Upper**
**L(T)**	0	.	.	.	.	.	.
**S*L(T)**	0.06716	0.09665	0.69	0.2436	0.05	0.01316	86.6573
**Residual**	0.5862	0.1263	4.64	<.0001	0.05	0.4003	0.9407

Fit Statistics	
**-2 Res Log Likelihood**	161.0
**AIC (Smaller is Better)**	165.0
**AICC (Smaller is Better)**	165.3
**BIC (Smaller is Better)**	165.2

Solution for Fixed Effects

Effect	T	Gen	Timing	S	Estimate	Standard Error	DF	t Value	Pr > |t|	Alpha	Lower	Upper
**Intercept**					36.9694	1.0309	52.5	35.86	<.0001	0.05	34.9012	39.0376
**T**	A				0.4002	0.3441	12.5	1.16	0.2666	0.05	-0.3463	1.1467
**T**	C				0	.	.	.	.	.	.	.
**S**				0	0.2717	0.3497	12.1	0.78	0.4521	0.05	-0.4894	1.0328
**S**				1	0	.	.	.	.	.	.	.
**T*S**	A			0	0.1541	0.4750	11.3	0.32	0.7516	0.05	-0.8883	1.1964
**T*S**	A			1	0	.	.	.	.	.	.	.
**T*S**	C			0	0	.	.	.	.	.	.	.
**T*S**	C			1	0	.	.	.	.	.	.	.
**Gen**		Y			0.04562	0.2951	51.3	0.15	0.8778	0.05	-0.5468	0.6381
**Gen**		Z			0	.	.	.	.	.	.	.
**Timing**			Afternoon		-0.1096	0.2074	52.4	-0.53	0.5995	0.05	-0.5258	0.3066
**Timing**			Morning		0	.	.	.	.	.	.	.
**MB0**					0.04429	0.04179	53.6	1.06	0.2939	0.05	-0.03950	0.1281
**Age**					-0.00001	0.004987	50.2	-0.00	0.9982	0.05	-0.01003	0.01000

Type 3 Tests of Fixed Effects

Effect	Num DF	Den DF	F Value	Pr > F
**T**	1	15.6	3.21	0.0925
**S**	1	14.5	1.79	0.2015
**T*S**	1	11.3	0.11	0.7516
**Gen**	1	51.3	0.02	0.8778
**Timing**	1	52.4	0.28	0.5995
**MB0**	1	53.6	1.12	0.2939
**Age**	1	50.2	0.00	0.9982

Least Squares Means												
**Effect**	**T**	**Gen**	**Timing**	**S**	**Estimate**	**Standard Error**	**DF**	**t Value**	**Pr > |t|**	**Alpha**	**Lower**	**Upper**
**T**	A				38.6562	0.1808	14.6	213.84	<.0001	0.05	38.2701	39.0424
**T**	C				38.1790	0.1754	12.4	217.67	<.0001	0.05	37.7981	38.5599
**S**				0	38.5920	0.1807	13.6	213.58	<.0001	0.05	38.2033	38.9806
**S**				1	38.2432	0.1713	12.3	223.31	<.0001	0.05	37.8711	38.6154
**T*S**	A			0	38.8691	0.2538	13.8	153.13	<.0001	0.05	38.3239	39.4143
**T*S**	A			1	38.4433	0.2532	14.6	151.81	<.0001	0.05	37.9023	38.9844
**T*S**	C			0	38.3149	0.2626	14.1	145.91	<.0001	0.05	37.7522	38.8775
**T*S**	C			1	38.0431	0.2318	10.3	164.12	<.0001	0.05	37.5287	38.5576
**Gen**		Y			38.4404	0.1925	39.9	199.69	<.0001	0.05	38.0513	38.8295
**Gen**		Z			38.3948	0.1857	38.1	206.71	<.0001	0.05	38.0188	38.7708
**Timing**			Afternoon		38.3628	0.1558	23.9	246.25	<.0001	0.05	38.0412	38.6844
**Timing**			Morning		38.4724	0.1589	29	242.14	<.0001	0.05	38.1475	38.7973

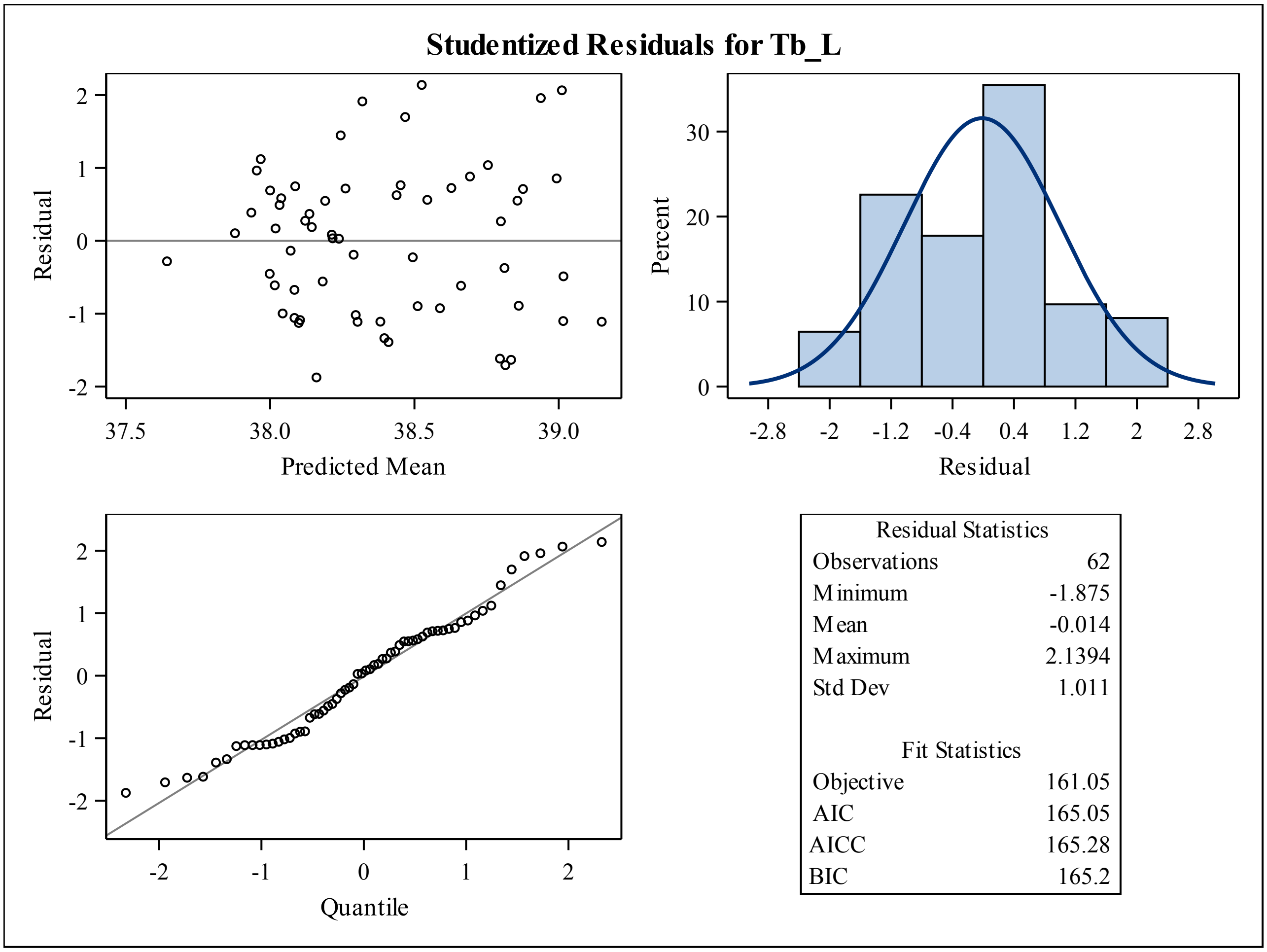

1.1.6. Output from SAS (v. 9.3) Mixed Model analysis for $T_b$mean at 34°C.

Model Information	
Data Set	WORK.THERMLONG
Dependent Variable	Tb_mean
Covariance Structure	Variance Components
Estimation Method	REML
Residual Variance Method	Profile
Fixed Effects SE Method	Model-Based
Degrees of Freedom Method	Satterthwaite

Class Level Information		
Class	Levels	Values
T	2	A C
S	2	0 1
L	8	A1 A2 A3 A4 C1 C2 C3 C4
Gen	2	Y Z
Timing	2	Afternoon Morning

Dimensions	
Covariance Parameters	3
Columns in X	15
Columns in Z	24
Subjects	1
Max Obs per Subject	58

Number of Observations	
Number of Observations Read	58
Number of Observations Used	58
Number of Observations Not Used	0

Covariance Parameter Estimates							
Cov Parm	Estimate	Standard Error	Z Value	Pr > Z	Alpha	Lower	Upper
L(T)	0	.	.	.	.	.	.
S*L(T)	0.7919	0.4819	1.64	0.0502	0.05	0.3171	4.3308
Residual	1.3383	0.3034	4.41	<.0001	0.05	0.8977	2.2079

Fit Statistics	
-2 Res Log Likelihood	201.4
AIC (Smaller is Better)	205.4
AICC (Smaller is Better)	205.6
BIC (Smaller is Better)	205.5

Solution for Fixed Effects

Effect	T	Gen	Timing	S	Estimate	Standard Error	DF	t Value	Pr > |t|	Alpha	Lower	Upper
Intercept					35.2811	1.8028	50	19.57	<.0001	0.05	31.6601	38.9022
T	A				0.8435	0.7748	12.5	1.09	0.2968	0.05	-0.8370	2.5240
T	C				0	.	.	.	.	.	.	.
S				0	0.2555	0.8030	13.6	0.32	0.7552	0.05	-1.4719	1.9829
S				1	0	.	.	.	.	.	.	.
T*S	A			0	-0.4434	1.0922	12.2	-0.41	0.6918	0.05	-2.8179	1.9310
T*S	A			1	0	.	.	.	.	.	.	.
T*S	C			0	0	.	.	.	.	.	.	.
T*S	C			1	0	.	.	.	.	.	.	.
Gen		Y			-0.1485	0.4866	44	-0.31	0.7617	0.05	-1.1293	0.8323
Gen		Z			0	.	.	.	.	.	.	.
Timing			Afternoon		0.3034	0.3429	44.4	0.88	0.3810	0.05	-0.3876	0.9944
Timing			Morning		0	.	.	.	.	.	.	.
MB0					0.1054	0.07166	48.5	1.47	0.1478	0.05	-0.03864	0.2494
Age					0.009528	0.008010	42.8	1.19	0.2408	0.05	-0.00663	0.02568

Type 3 Tests of Fixed Effects

Effect	Num DF	Den DF	F Value	Pr > F
T	1	15.1	1.14	0.3034
S	1	14.9	0.00	0.9545
T*S	1	12.2	0.16	0.6918
Gen	1	44	0.09	0.7617
Timing	1	44.4	0.78	0.3810
MB0	1	48.5	2.16	0.1478
Age	1	42.8	1.41	0.2408

Least Squares Means												
**Effect**	**T**	**Gen**	**Timing**	**S**	**Estimate**	**Standard Error**	**DF**	**t Value**	**Pr > |t|**	**Alpha**	**Lower**	**Upper**
**T**	A				40.0771	0.3964	13.6	101.09	<.0001	0.05	39.2243	40.9299
**T**	C				39.4553	0.4027	13.8	97.99	<.0001	0.05	38.5905	40.3200
**S**				0	39.7831	0.4118	14.9	96.60	<.0001	0.05	38.9045	40.6616
**S**				1	39.7493	0.3857	12.3	103.05	<.0001	0.05	38.9108	40.5877
**T*S**	A			0	39.9831	0.5639	13.8	70.90	<.0001	0.05	38.7721	41.1941
**T*S**	A			1	40.1710	0.5576	13.3	72.04	<.0001	0.05	38.9692	41.3728
**T*S**	C			0	39.5830	0.5999	15.9	65.98	<.0001	0.05	38.3104	40.8556
**T*S**	C			1	39.3275	0.5356	11.5	73.43	<.0001	0.05	38.1548	40.5002
**Gen**		Y			39.6919	0.3779	32.2	105.04	<.0001	0.05	38.9224	40.4615
**Gen**		Z			39.8404	0.3530	27.8	112.85	<.0001	0.05	39.1170	40.5638
**Timing**			Afternoon		39.9179	0.3290	22.2	121.34	<.0001	0.05	39.2360	40.5997
**Timing**			Morning		39.6145	0.3156	20.5	125.53	<.0001	0.05	38.9571	40.2718

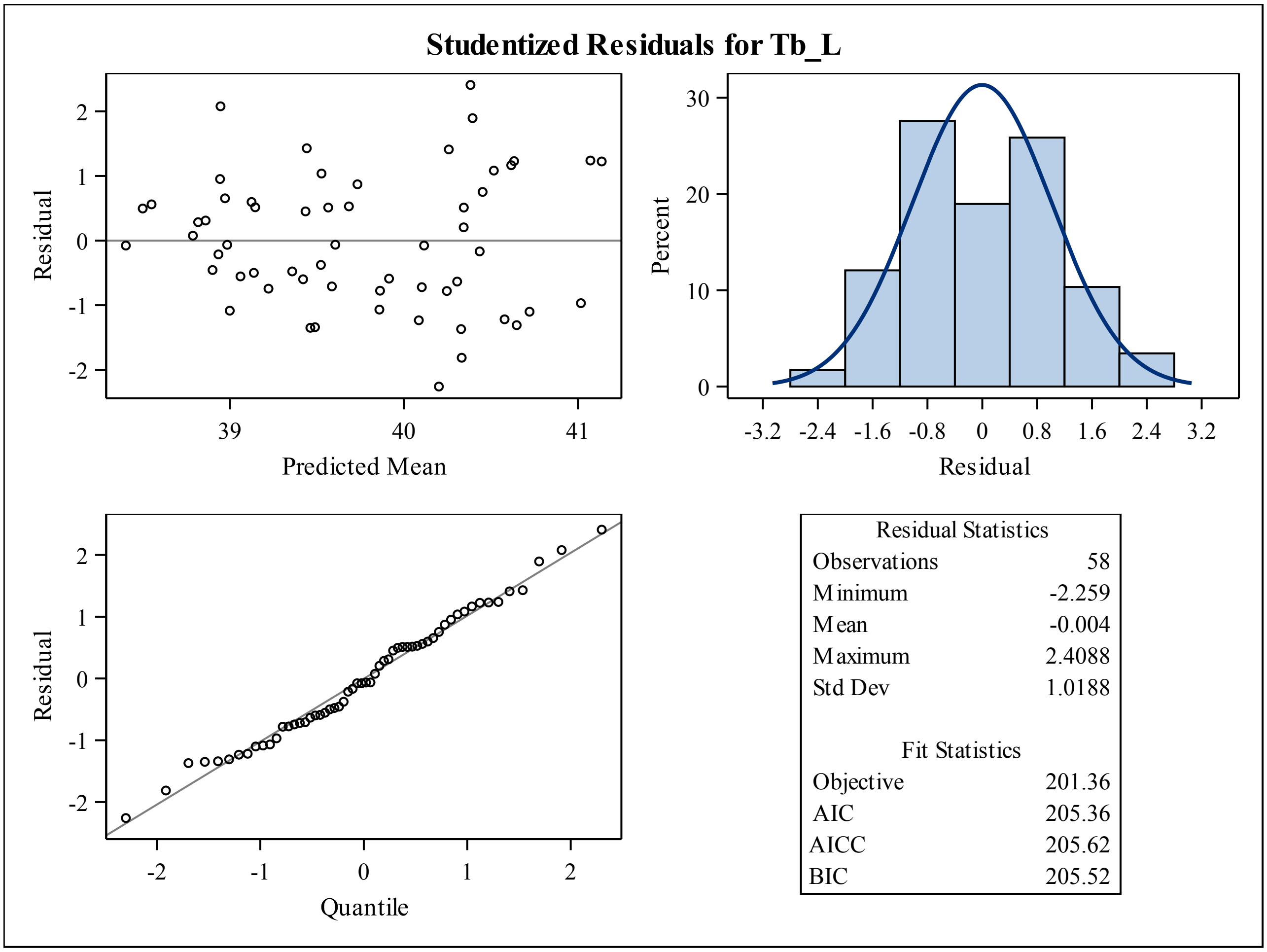

1.2. $T_b$rmr: $T_b$rmr is the T_b recorded at the time of the lowest RMR measurement

1.2.1. Output from SAS (v. 9.3) Mixed Model analysis for $T_b$rmr at 10°C.

Model Information	
Data Set	WORK.THERMLONG
Dependent Variable	Tb_rmr
Covariance Structure	Variance Components
Estimation Method	REML
Residual Variance Method	Profile
Fixed Effects SE Method	Model-Based
Degrees of Freedom Method	Satterthwaite

Class Level Information		
Class	Levels	Values
T	2	A C
S	2	0 1
L	8	A1 A2 A3 A4 C1 C2 C3 C4
Gen	2	Y Z
Timing	2	Afternoon Morning

Dimensions	
Covariance Parameters	3
Columns in X	15
Columns in Z	24
Subjects	1
Max Obs per Subject	106

Number of Observations	
Number of Observations Read	106
Number of Observations Used	106
Number of Observations Not Used	0

Covariance Parameter Estimates							
Cov Parm	Estimate	Standard Error	Z Value	Pr > Z	Alpha	Lower	Upper
L(T)	0.02938	0.05398	0.54	0.2931	0.05	0.004612	3203.18
S*L(T)	0.04505	0.05403	0.83	0.2022	0.05	0.01041	7.2367
Residual	0.3060	0.04670	6.55	<.0001	0.05	0.2318	0.4230

Fit Statistics	
**-2 Res Log Likelihood**	209.9
**AIC (Smaller is Better)**	215.9
**AICC (Smaller is Better)**	216.1
**BIC (Smaller is Better)**	216.1

Solution for Fixed Effects

Effect	T	Gen	Timing	S	Estimate	Standard Error	DF	t Value	Pr > |t|	Alpha	Lower	Upper
**Intercept**					38.2493	0.5819	86	65.73	<.0001	0.05	37.0925	39.4061
**T**	A				-0.08796	0.2577	12.2	-0.34	0.7387	0.05	-0.6485	0.4726
**T**	C				0	.	.	.	.	.	.	.
**S**				0	-0.01929	0.2231	6.82	-0.09	0.9336	0.05	-0.5496	0.5110
**S**				1	0	.	.	.	.	.	.	.
**T*S**	A			0	0.07199	0.3035	5.89	0.24	0.8206	0.05	-0.6740	0.8180
**T*S**	A			1	0	.	.	.	.	.	.	.
**T*S**	C			0	0	.	.	.	.	.	.	.
**T*S**	C			1	0	.	.	.	.	.	.	.
**Gen**		Y			0.02759	0.1727	88.7	0.16	0.8734	0.05	-0.3156	0.3707
**Gen**		Z			0	.	.	.	.	.	.	.
**Timing**			Afternoon		0.1015	0.1157	94.7	0.88	0.3830	0.05	-0.1283	0.3313
**Timing**			Morning		0	.	.	.	.	.	.	.
**MB0**					-0.00390	0.02118	95.1	-0.18	0.8542	0.05	-0.04595	0.03815
**Age**					0.000841	0.002790	88.5	0.30	0.7639	0.05	-0.00470	0.006385

Type 3 Tests of Fixed Effects

Effect	Num DF	Den DF	F Value	Pr > F
**T**	1	6.44	0.06	0.8077
**S**	1	8.41	0.01	0.9226
**T*S**	1	5.89	0.06	0.8206
**Gen**	1	88.7	0.03	0.8734
**Timing**	1	94.7	0.77	0.3830
**MB0**	1	95.1	0.03	0.8542
**Age**	1	88.5	0.09	0.7639

Least Squares Means												
**Effect**	**T**	**Gen**	**Timing**	**S**	**Estimate**	**Standard Error**	**DF**	**t Value**	**Pr > |t|**	**Alpha**	**Lower**	**Upper**
**T**	A				38.2724	0.1410	5.85	271.52	<.0001	0.05	37.9252	38.6195
**T**	C				38.3243	0.1417	5.93	270.51	<.0001	0.05	37.9766	38.6720
**S**				0	38.3067	0.1291	12.2	296.82	<.0001	0.05	38.0260	38.5874
**S**				1	38.2900	0.1274	11.7	300.66	<.0001	0.05	38.0117	38.5683
**T*S**	A			0	38.2987	0.1756	10.8	218.16	<.0001	0.05	37.9114	38.6860
**T*S**	A			1	38.2460	0.1869	13.3	204.67	<.0001	0.05	37.8431	38.6489
**T*S**	C			0	38.3147	0.1852	12.7	206.84	<.0001	0.05	37.9135	38.7159
**T*S**	C			1	38.3340	0.1753	10.7	218.72	<.0001	0.05	37.9467	38.7212
**Gen**		Y			38.3121	0.1347	18.7	284.47	<.0001	0.05	38.0300	38.5943
**Gen**		Z			38.2845	0.1254	14.4	305.38	<.0001	0.05	38.0164	38.5527
**Timing**			Afternoon		38.3491	0.1142	9.91	335.90	<.0001	0.05	38.0944	38.6038
**Timing**			Morning		38.2476	0.1123	9.38	340.62	<.0001	0.05	37.9952	38.5000

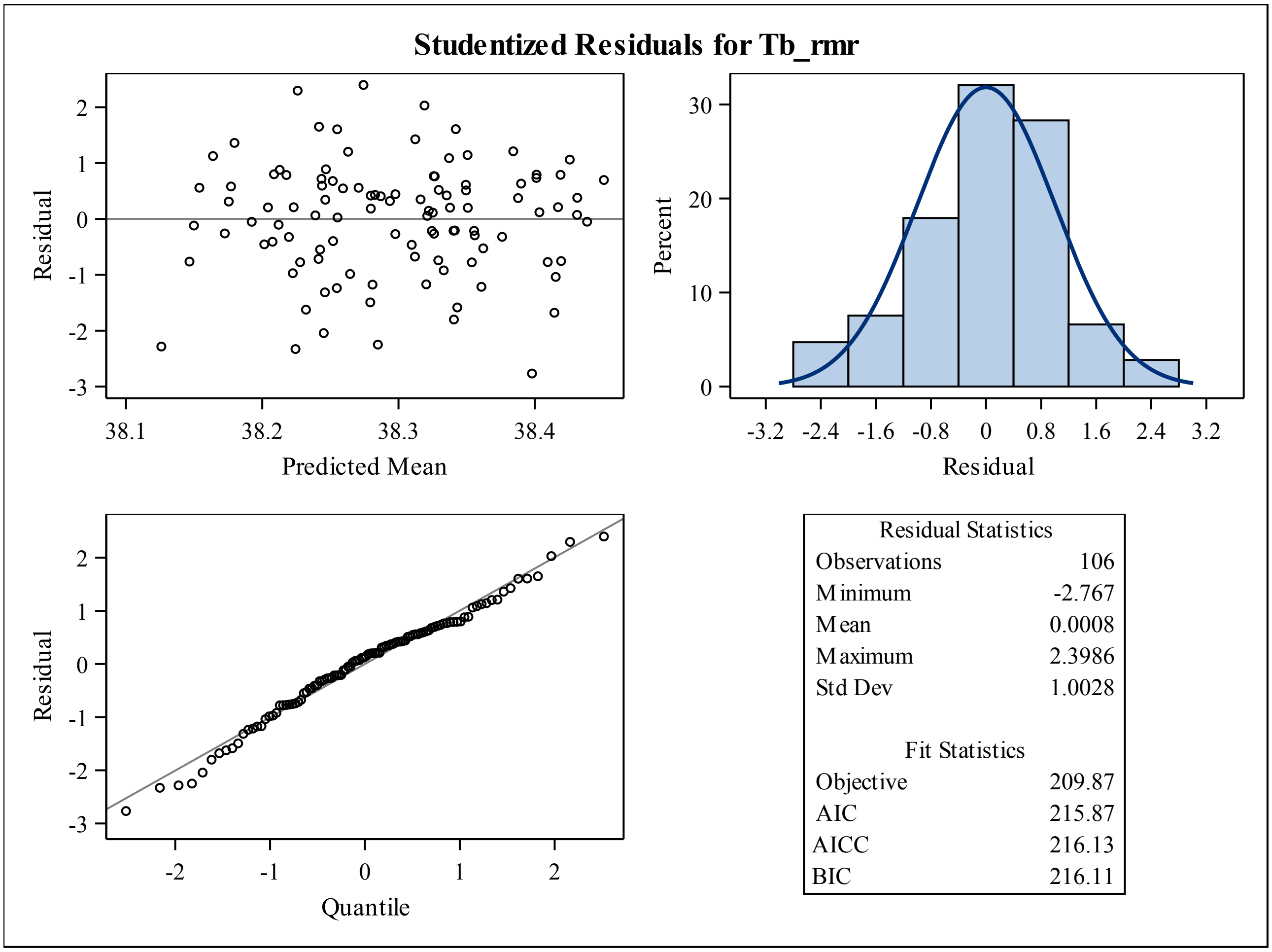

1.2.2. Output from SAS (v. 9.3) Mixed Model analysis for $T_b$rmr at 20°C.

Model Information	
**Data Set**	WORK.THERMLONG
**Dependent Variable**	Tb_rmr
**Covariance Structure**	Variance Components
**Estimation Method**	REML
**Residual Variance Method**	Profile
**Fixed Effects SE Method**	Model-Based
**Degrees of Freedom Method**	Satterthwaite

Class Level Information		
**Class**	**Levels**	**Values**
**T**	2	A C
**S**	2	0 1
**L**	8	A1 A2 A3 A4 C1 C2 C3 C4
**Gen**	2	Y Z
**Timing**	2	Afternoon Morning

Dimensions	
**Covariance Parameters**	3
**Columns in X**	15
**Columns in Z**	24
**Subjects**	1
**Max Obs per Subject**	129

Number of Observations	
**Number of Observations Read**	129
**Number of Observations Used**	129
**Number of Observations Not Used**	0

Covariance Parameter Estimates							
**Cov Parm**	**Estimate**	**Standard Error**	**Z Value**	**Pr > Z**	**Alpha**	**Lower**	**Upper**
**L(T)**	0.02129	0.02589	0.82	0.2055	0.05	0.004857	3.9038
**S*L(T)**	0	.	.	.	.	.	.
**Residual**	0.3734	0.04917	7.59	<.0001	0.05	0.2931	0.4921

Fit Statistics	
-2 Res Log Likelihood	266.9
AIC (Smaller is Better)	270.9
AICC (Smaller is Better)	271.0
BIC (Smaller is Better)	271.0

Solution for Fixed Effects

Effect	T	Gen	Timing	S	Estimate	Standard Error	DF	t Value	Pr > |t|	Alpha	Lower	Upper
Intercept					38.4805	0.5188	102	74.17	<.0001	0.05	37.4514	39.5096
T	A				-0.07108	0.1958	16.3	-0.36	0.7213	0.05	-0.4856	0.3434
T	C				0	.	.	.	.	.	.	.
S				0	-0.1524	0.1638	120	-0.93	0.3539	0.05	-0.4767	0.1718
S				1	0	.	.	.	.	.	.	.
T*S	A			0	0.3048	0.2167	116	1.41	0.1622	0.05	-0.1243	0.7340
T*S	A			1	0	.	.	.	.	.	.	.
T*S	C			0	0	.	.	.	.	.	.	.
T*S	C			1	0	.	.	.	.	.	.	.
Gen		Y			-0.3012	0.1532	118	-1.97	0.0516	0.05	-0.6046	0.002189
Gen		Z			0	.	.	.	.	.	.	.
Timing			Afternoon		0.02777	0.1096	119	0.25	0.8004	0.05	-0.1893	0.2448
Timing			Morning		0	.	.	.	.	.	.	.
MB0					-0.04165	0.01916	106	-2.17	0.0319	0.05	-0.07963	-0.00367
Age					0.005107	0.002471	118	2.07	0.0410	0.05	0.000213	0.01000

Type 3 Tests of Fixed Effects

Effect	Num DF	Den DF	F Value	Pr > F
T	1	7.69	0.26	0.6268
S	1	121	0.00	0.9998
T*S	1	116	1.98	0.1622
Gen	1	118	3.86	0.0516
Timing	1	119	0.06	0.8004
MB0	1	106	4.73	0.0319
Age	1	118	4.27	0.0410

Least Squares Means												
**Effect**	**T**	**Gen**	**Timing**	**S**	**Estimate**	**Standard Error**	**DF**	**t Value**	**Pr > |t|**	**Alpha**	**Lower**	**Upper**
**T**	A				38.0226	0.1089	6.62	349.17	<.0001	0.05	37.7621	38.2831
**T**	C				37.9413	0.1107	7.1	342.75	<.0001	0.05	37.6803	38.2023
**S**				0	37.9819	0.09811	16.4	387.15	<.0001	0.05	37.7744	38.1895
**S**				1	37.9820	0.09740	15.7	389.94	<.0001	0.05	37.7752	38.1888
**T*S**	A			0	38.0988	0.1318	14.3	288.97	<.0001	0.05	37.8166	38.3810
**T*S**	A			1	37.9464	0.1431	17.4	265.25	<.0001	0.05	37.6451	38.2478
**T*S**	C			0	37.8651	0.1423	17.5	266.13	<.0001	0.05	37.5655	38.1646
**T*S**	C			1	38.0175	0.1330	14.6	285.92	<.0001	0.05	37.7334	38.3016
**Gen**		Y			37.8313	0.1039	20.8	364.04	<.0001	0.05	37.6151	38.0476
**Gen**		Z			38.1326	0.1102	25.8	345.97	<.0001	0.05	37.9059	38.3592
**Timing**			Afternoon		37.9958	0.09308	14	408.22	<.0001	0.05	37.7962	38.1955
**Timing**			Morning		37.9681	0.09247	13.6	410.59	<.0001	0.05	37.7692	38.1669

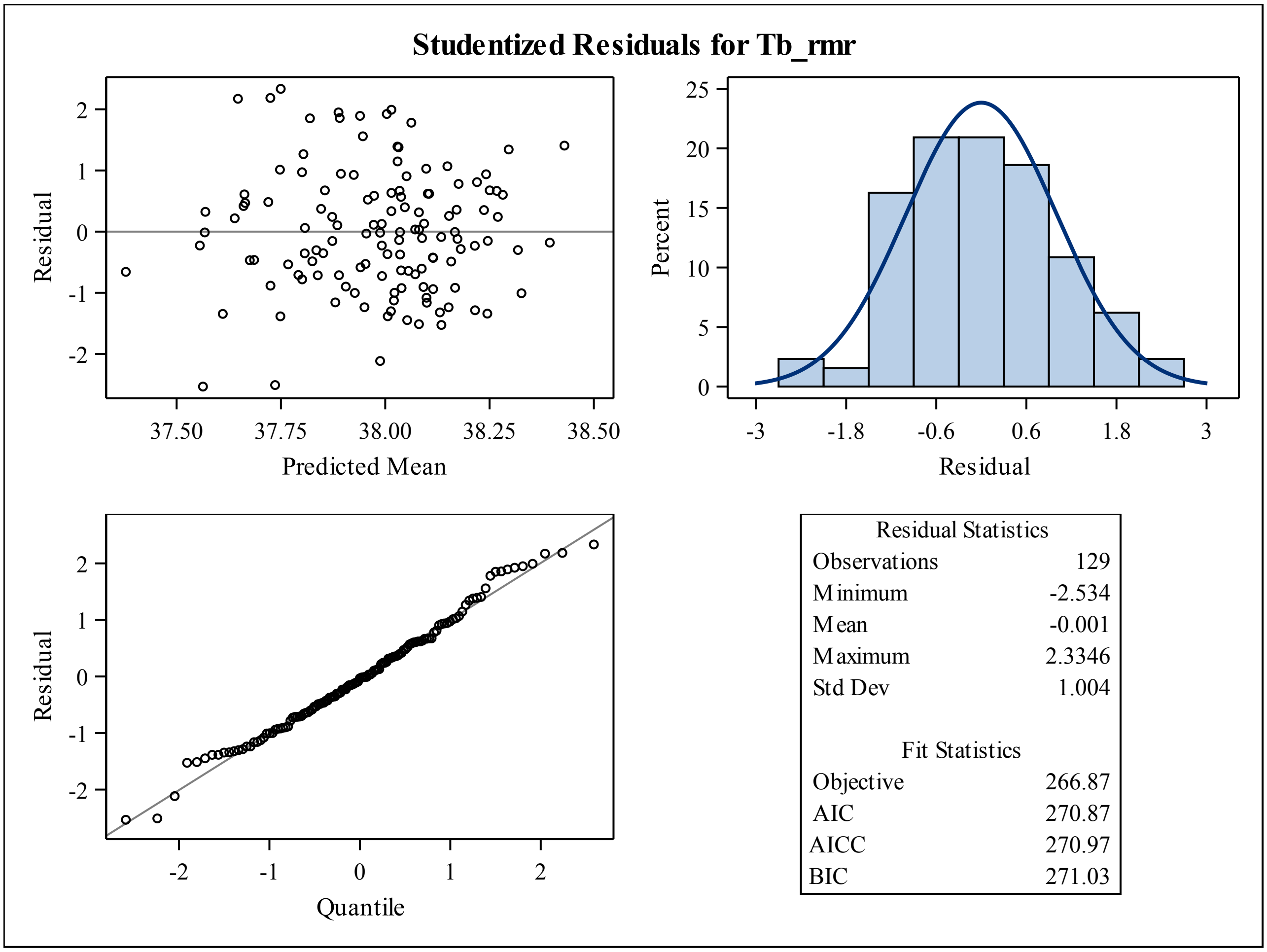

1.2.3. Output from SAS (v. 9.3) Mixed Model analysis for $T_b$rmr at 25°C.

Model Information	
**Data Set**	WORK.THERMLONG
**Dependent Variable**	Tb_rmr
**Covariance Structure**	Variance Components
**Estimation Method**	REML
**Residual Variance Method**	Profile
**Fixed Effects SE Method**	Model-Based
**Degrees of Freedom Method**	Satterthwaite

Class Level Information		
**Class**	**Levels**	**Values**
**T**	2	A C
**S**	2	0 1
**L**	8	A1 A2 A3 A4 C1 C2 C3 C4
**Gen**	2	Y Z
**Timing**	2	Afternoon Morning

Dimensions	
**Covariance Parameters**	3
**Columns in X**	15
**Columns in Z**	24
**Subjects**	1
**Max Obs per Subject**	63

Number of Observations	
**Number of Observations Read**	63
**Number of Observations Used**	63
**Number of Observations Not Used**	0

Covariance Parameter Estimates							
**Cov Parm**	**Estimate**	**Standard Error**	**Z Value**	**Pr > Z**	**Alpha**	**Lower**	**Upper**
**L(T)**	0	.	.	.	.	.	.
**S*L(T)**	0	.	.	.	.	.	.
**Residual**	0.3767	0.07183	5.24	<.0001	0.05	0.2677	0.5692

Fit Statistics	
**-2 Res Log Likelihood**	135.2
**AIC (Smaller is Better)**	137.2
**AICC (Smaller is Better)**	137.3
**BIC (Smaller is Better)**	137.3

Solution for Fixed Effects

Effect	T	Gen	Timing	S	Estimate	Standard Error	DF	t Value	Pr > |t|	Alpha	Lower	Upper
**Intercept**					38.9629	0.7585	55	51.37	<.0001	0.05	37.4429	40.4830
**T**	A				0.4222	0.2300	55	1.84	0.0718	0.05	-0.03877	0.8832
**T**	C				0	.	.	.	.	.	.	.
**S**				0	-0.01778	0.2335	55	-0.08	0.9396	0.05	-0.4857	0.4502
**S**				1	0	.	.	.	.	.	.	.
**T*S**	A			0	-0.07641	0.3116	55	-0.25	0.8072	0.05	-0.7008	0.5480
**T*S**	A			1	0	.	.	.	.	.	.	.
**T*S**	C			0	0	.	.	.	.	.	.	.
**T*S**	C			1	0	.	.	.	.	.	.	.
**Gen**		Y			0.1321	0.2155	55	0.61	0.5423	0.05	-0.2997	0.5639
**Gen**		Z			0	.	.	.	.	.	.	.
**Timing**			Afternoon		-0.1862	0.1583	55	-1.18	0.2444	0.05	-0.5033	0.1310
**Timing**			Morning		0	.	.	.	.	.	.	.
**MB0**					-0.03649	0.03178	55	-1.15	0.2558	0.05	-0.1002	0.02719
**Age**					-0.00130	0.003628	55	-0.36	0.7213	0.05	-0.00857	0.005970

Type 3 Tests of Fixed Effects

Effect	Num DF	Den DF	F Value	Pr > F
**T**	1	55	4.75	0.0337
**S**	1	55	0.10	0.7507
**T*S**	1	55	0.06	0.8072
**Gen**	1	55	0.38	0.5423
**Timing**	1	55	1.38	0.2444
**MB0**	1	55	1.32	0.2558
**Age**	1	55	0.13	0.7213

Least Squares Means												
**Effect**	**T**	**Gen**	**Timing**	**S**	**Estimate**	**Standard Error**	**DF**	**t Value**	**Pr > |t|**	**Alpha**	**Lower**	**Upper**
**T**	A				38.2166	0.1185	55	322.41	<.0001	0.05	37.9791	38.4542
**T**	C				37.8327	0.1167	55	324.24	<.0001	0.05	37.5988	38.0665
**S**				0	37.9967	0.1197	55	317.50	<.0001	0.05	37.7568	38.2365
**S**				1	38.0526	0.1148	55	331.45	<.0001	0.05	37.8226	38.2827
**T*S**	A			0	38.1696	0.1613	55	236.71	<.0001	0.05	37.8464	38.4927
**T*S**	A			1	38.2637	0.1727	55	221.50	<.0001	0.05	37.9176	38.6099
**T*S**	C			0	37.8238	0.1775	55	213.06	<.0001	0.05	37.4680	38.1795
**T*S**	C			1	37.8416	0.1516	55	249.66	<.0001	0.05	37.5378	38.1453
**Gen**		Y			38.0907	0.1324	55	287.65	<.0001	0.05	37.8253	38.3561
**Gen**		Z			37.9586	0.1335	55	284.41	<.0001	0.05	37.6911	38.2261
**Timing**			Afternoon		37.9316	0.1071	55	354.08	<.0001	0.05	37.7169	38.1462
**Timing**			Morning		38.1178	0.1148	55	332.02	<.0001	0.05	37.8877	38.3478

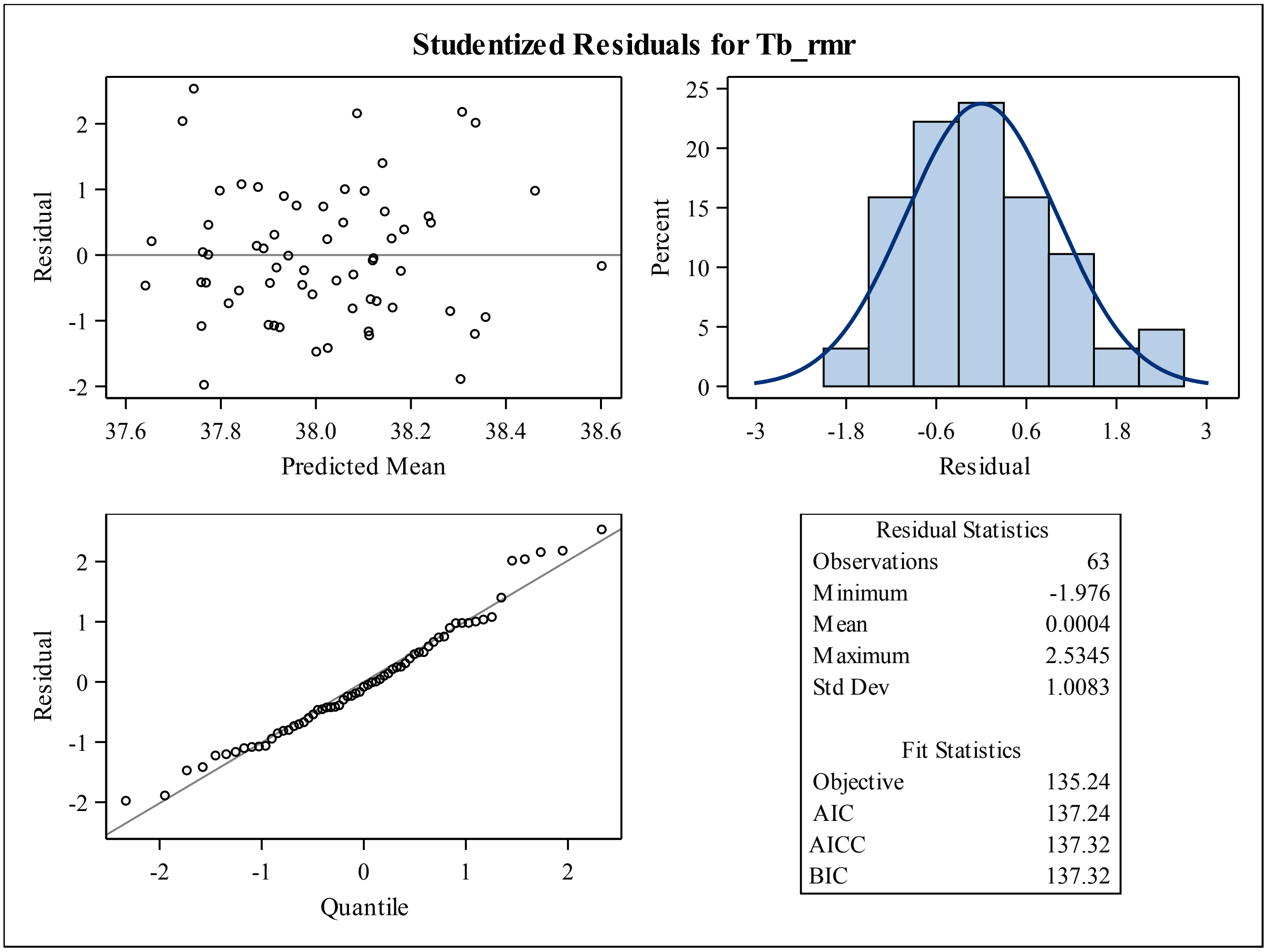

1.2.4. Output from SAS (v. 9.3) Mixed Model analysis for $T_b$rmr at 28°C.

Model Information	
Data Set	WORK.THERMLONG
Dependent Variable	Tb_rmr
Covariance Structure	Variance Components
Estimation Method	REML
Residual Variance Method	Profile
Fixed Effects SE Method	Model-Based
Degrees of Freedom Method	Satterthwaite

Class Level Information		
Class	Levels	Values
T	2	A C
S	2	0 1
L	8	A1 A2 A3 A4 C1 C2 C3 C4
Gen	2	Y Z
Timing	2	Afternoon Morning

Dimensions	
Covariance Parameters	3
Columns in X	15
Columns in Z	24
Subjects	1
Max Obs per Subject	60

Number of Observations	
Number of Observations Read	60
Number of Observations Used	60
Number of Observations Not Used	0

Covariance Parameter Estimates							
Cov Parm	Estimate	Standard Error	Z Value	Pr > Z	Alpha	Lower	Upper
L(T)	0.007552	0.04154	0.18	0.4279	0.05	0.000668	1.274E45
S*L(T)	0	.	.	.	.	.	.
Residual	0.3292	0.07084	4.65	<.0001	0.05	0.2249	0.5279

Fit Statistics	
**-2 Res Log Likelihood**	123.0
**AIC (Smaller is Better)**	127.0
**AICC (Smaller is Better)**	127.2
**BIC (Smaller is Better)**	127.1

Solution for Fixed Effects													
**Effect**	**T**	**Gen**	**Timing**	**S**	**Estimate**	**Standard Error**	**DF**	**t Value**	**Pr > |t|**	**Alpha**	**Lower**	**Upper**
**Intercept**					38.3412	0.7573	48.8	50.63	<.0001	0.05	36.8192	39.8632
**T**	A				0.1769	0.2253	12.8	0.79	0.4465	0.05	-0.3104	0.6642
**T**	C				0	.	.	.	.	.	.	.
**S**				0	0.09169	0.2292	51.2	0.40	0.6908	0.05	-0.3684	0.5517
**S**				1	0	.	.	.	.	.	.	.
**T*S**	A			0	0.1040	0.3019	48.2	0.34	0.7321	0.05	-0.5030	0.7110
**T*S**	A			1	0	.	.	.	.	.	.	.
**T*S**	C			0	0	.	.	.	.	.	.	.
**T*S**	C			1	0	.	.	.	.	.	.	.
**Gen**		Y			0.1612	0.2168	47.2	0.74	0.4608	0.05	-0.2749	0.5974
**Gen**		Z			0	.	.	.	.	.	.	.
**Timing**			Afternoon		-0.1588	0.1536	49.2	-1.03	0.3062	0.05	-0.4675	0.1499
**Timing**			Morning		0	.	.	.	.	.	.	.
**MB0**					0.000975	0.03053	46.2	0.03	0.9747	0.05	-0.06047	0.06241
**Age**					-0.00375	0.003730	48.3	-1.01	0.3195	0.05	-0.01125	0.003747

Type 3 Tests of Fixed Effects				
**Effect**	**Num DF**	**Den DF**	**F Value**	**Pr > F**
**T**	1	5.89	1.50	0.2667
**S**	1	51.8	0.71	0.4041
**T*S**	1	48.2	0.12	0.7321
**Gen**	1	47.2	0.55	0.4608
**Timing**	1	49.2	1.07	0.3062
**MB0**	1	46.2	0.00	0.9747
**Age**	1	48.3	1.01	0.3195

Least Squares Means												
**Effect**	**T**	**Gen**	**Timing**	**S**	**Estimate**	**Standard Error**	**DF**	**t Value**	**Pr > |t|**	**Alpha**	**Lower**	**Upper**
**T**	A				38.1163	0.1237	4.8	308.11	<.0001	0.05	37.7943	38.4384
**T**	C				37.8874	0.1233	4.45	307.38	<.0001	0.05	37.5584	38.2164
**S**				0	38.0737	0.1229	14	309.81	<.0001	0.05	37.8102	38.3373
**S**				1	37.9300	0.1121	11.8	338.37	<.0001	0.05	37.6854	38.1747
**T*S**	A			0	38.2142	0.1674	13.8	228.23	<.0001	0.05	37.8547	38.5737
**T*S**	A			1	38.0185	0.1681	15	226.11	<.0001	0.05	37.6600	38.3770
**T*S**	C			0	37.9333	0.1855	16.7	204.49	<.0001	0.05	37.5414	38.3251
**T*S**	C			1	37.8416	0.1491	9.8	253.76	<.0001	0.05	37.5084	38.1748
**Gen**		Y			38.0825	0.1373	22.8	277.27	<.0001	0.05	37.7982	38.3668
**Gen**		Z			37.9213	0.1331	22.9	284.84	<.0001	0.05	37.6458	38.1967
**Timing**			Afternoon		37.9225	0.1111	11.1	341.39	<.0001	0.05	37.6782	38.1667
**Timing**			Morning		38.0813	0.1120	12.4	340.00	<.0001	0.05	37.8381	38.3245

Studentized Residuals for Tb_rmr

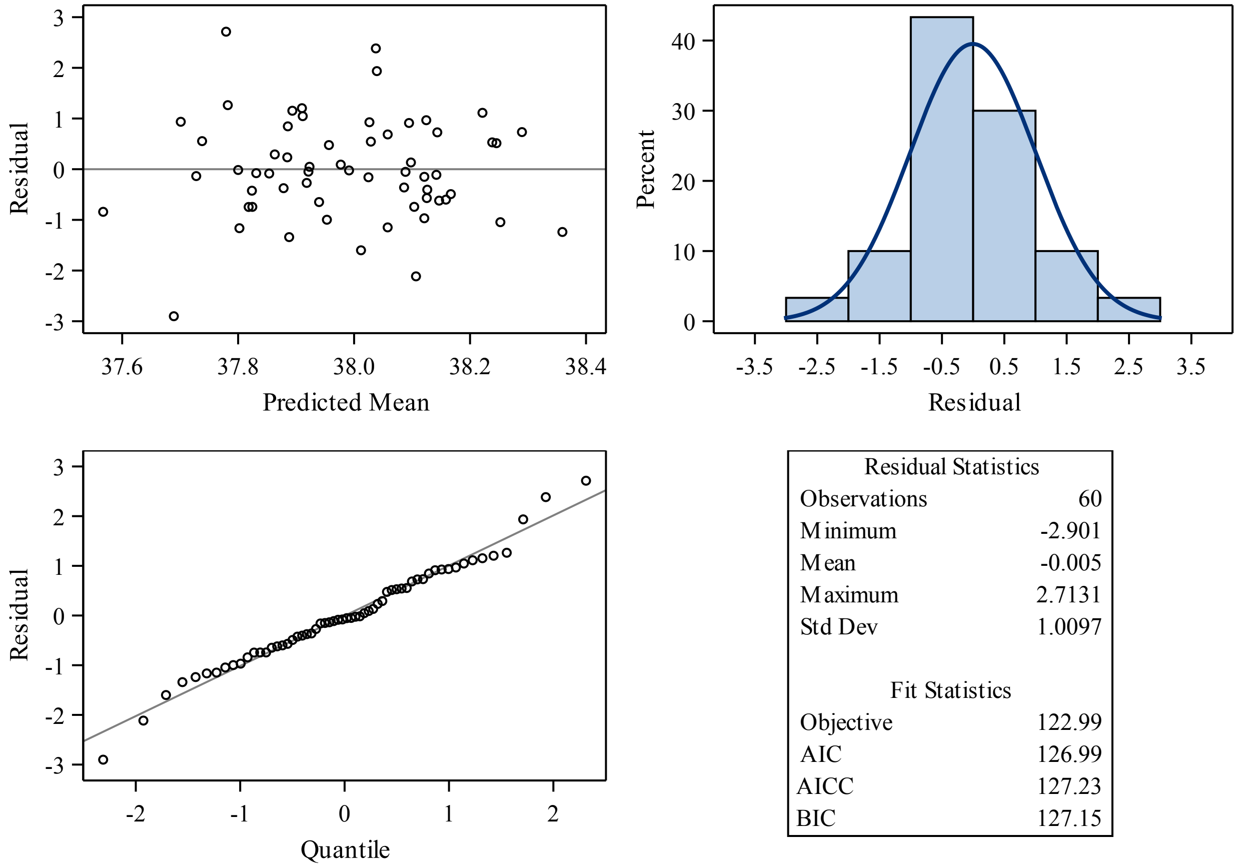

Residual Statistics	
Observations	60
Minimum	-2.901
Mean	-0.005
Maximum	2.7131
Std Dev	1.0097
**Fit Statistics**	
Objective	122.99
AIC	126.99
AICC	127.23
BIC	127.15

1.2.5. Output from SAS (v. 9.3) Mixed Model analysis for $T_b$rmr at 31°C.

Model Information	
**Data Set**	WORK.THERMLONG
**Dependent Variable**	Tb_rmr
**Covariance Structure**	Variance Components
**Estimation Method**	REML
**Residual Variance Method**	Profile
**Fixed Effects SE Method**	Model-Based
**Degrees of Freedom Method**	Satterthwaite

Class Level Information		
**Class**	**Levels**	**Values**
**T**	2	A C
**S**	2	0 1
**L**	8	A1 A2 A3 A4 C1 C2 C3 C4
**Gen**	2	Y Z
**Timing**	2	Afternoon Morning

Dimensions	
**Covariance Parameters**	3
**Columns in X**	15
**Columns in Z**	24
**Subjects**	1
**Max Obs per Subject**	62

Number of Observations	
**Number of Observations Read**	62
**Number of Observations Used**	62
**Number of Observations Not Used**	0

Covariance Parameter Estimates							
**Cov Parm**	**Estimate**	**Standard Error**	**Z Value**	**Pr > Z**	**Alpha**	**Lower**	**Upper**
**L(T)**	0	.	.	.	.	.	.
**S*L(T)**	0.02142	0.05991	0.36	0.3603	0.05	0.002380	1.496E10
**Residual**	0.4110	0.08913	4.61	<.0001	0.05	0.2801	0.6618

Fit Statistics	
**-2 Res Log Likelihood**	139.8
**AIC (Smaller is Better)**	143.8
**AICC (Smaller is Better)**	144.1
**BIC (Smaller is Better)**	144.0

Solution for Fixed Effects

Effect	T	Gen	Timing	S	Estimate	Standard Error	DF	t Value	Pr > |t|	Alpha	Lower	Upper
**Intercept**					37.9385	0.8406	50.9	45.13	<.0001	0.05	36.2508	39.6262
**T**	A				0.1564	0.2639	11.6	0.59	0.5648	0.05	-0.4207	0.7335
**T**	C				0	.	.	.	.	.	.	.
**S**				0	0.1802	0.2677	10.9	0.67	0.5149	0.05	-0.4095	0.7698
**S**				1	0	.	.	.	.	.	.	.
**T*S**	A			0	0.1767	0.3624	10.2	0.49	0.6361	0.05	-0.6281	0.9814
**T*S**	A			1	0	.	.	.	.	.	.	.
**T*S**	C			0	0	.	.	.	.	.	.	.
**T*S**	C			1	0	.	.	.	.	.	.	.
**Gen**		Y			-0.1163	0.2444	52	-0.48	0.6363	0.05	-0.6067	0.3742
**Gen**		Z			0	.	.	.	.	.	.	.
**Timing**			Afternoon		-0.2641	0.1714	53.1	-1.54	0.1292	0.05	-0.6079	0.07963
**Timing**			Morning		0	.	.	.	.	.	.	.
**MB0**					0.002854	0.03417	52.4	0.08	0.9338	0.05	-0.06570	0.07141
**Age**					0.000656	0.004137	50.9	0.16	0.8747	0.05	-0.00765	0.008962

Type 3 Tests of Fixed Effects

Effect	Num DF	Den DF	F Value	Pr > F
**T**	1	14.6	1.41	0.2542
**S**	1	13.5	1.78	0.2041
**T*S**	1	10.2	0.24	0.6361
**Gen**	1	52	0.23	0.6363
**Timing**	1	53.1	2.37	0.1292
**MB0**	1	52.4	0.01	0.9338
**Age**	1	50.9	0.03	0.8747

Least Squares Means												
**Effect**	**T**	**Gen**	**Timing**	**S**	**Estimate**	**Standard Error**	**DF**	**t Value**	**Pr > |t|**	**Alpha**	**Lower**	**Upper**
**T**	A				38.2463	0.1396	13.9	273.89	<.0001	0.05	37.9466	38.5459
**T**	C				38.0015	0.1344	11.2	282.82	<.0001	0.05	37.7063	38.2967
**S**				0	38.2581	0.1390	12.5	275.16	<.0001	0.05	37.9565	38.5597
**S**				1	37.9896	0.1312	11.4	289.50	<.0001	0.05	37.7021	38.2772
**T*S**	A			0	38.4247	0.1955	12.8	196.56	<.0001	0.05	38.0018	38.8476
**T*S**	A			1	38.0678	0.1957	14.1	194.56	<.0001	0.05	37.6485	38.4872
**T*S**	C			0	38.0916	0.2024	12.9	188.23	<.0001	0.05	37.6539	38.5293
**T*S**	C			1	37.9114	0.1760	9.14	215.41	<.0001	0.05	37.5142	38.3087
**Gen**		Y			38.0658	0.1548	40.5	245.95	<.0001	0.05	37.7531	38.3784
**Gen**		Z			38.1820	0.1491	39	256.13	<.0001	0.05	37.8805	38.4836
**Timing**			Afternoon		37.9918	0.1227	23.1	309.58	<.0001	0.05	37.7380	38.2456
**Timing**			Morning		38.2560	0.1262	30.2	303.07	<.0001	0.05	37.9982	38.5137

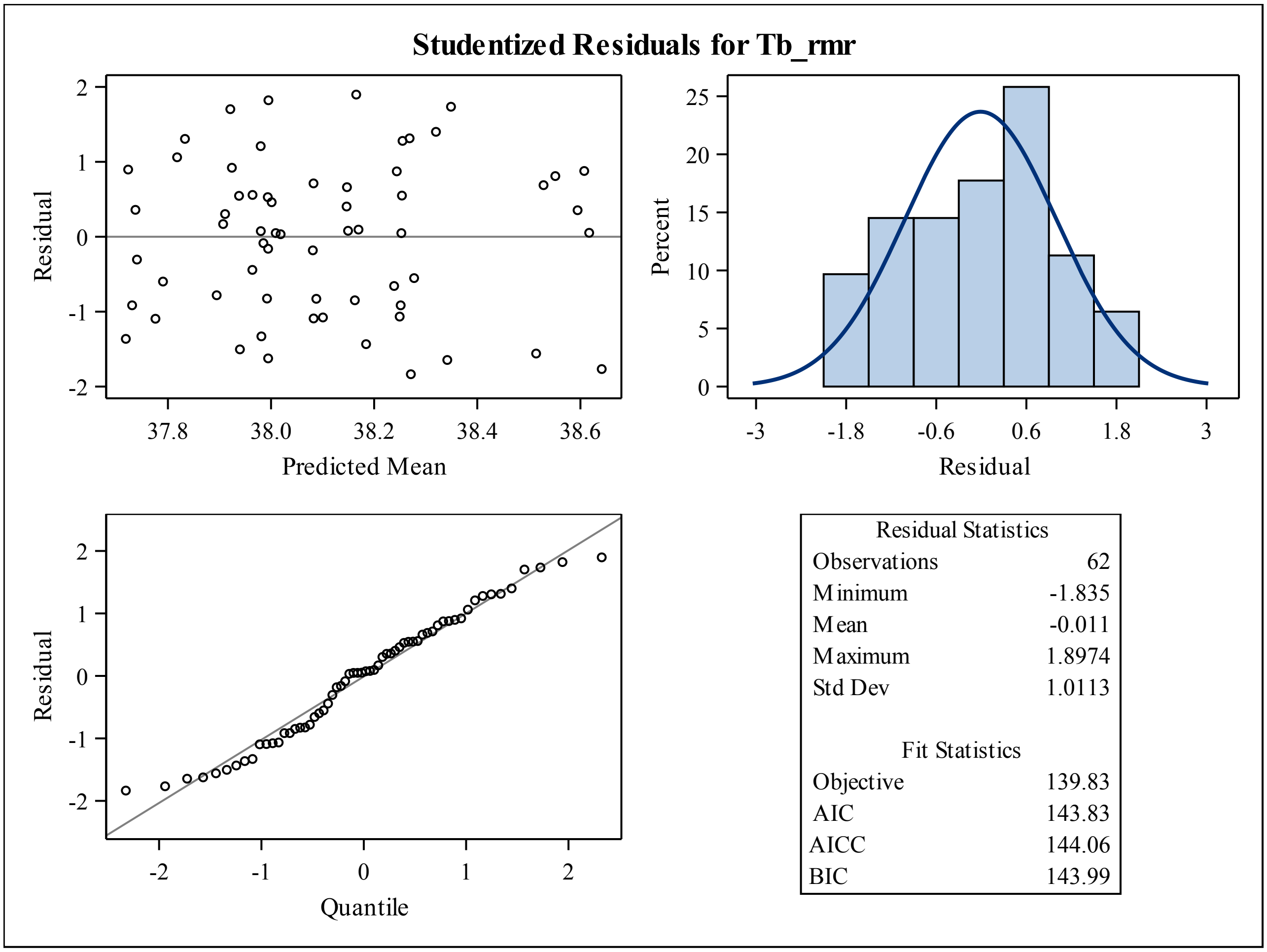

1.2.6. Output from SAS (v. 9.3) Mixed Model analysis for $T_b$rmr at 34°C.

Model Information	
**Data Set**	WORK.THERMLONG
**Dependent Variable**	Tb_rmr
**Covariance Structure**	Variance Components
**Estimation Method**	REML
**Residual Variance Method**	Profile
**Fixed Effects SE Method**	Model-Based
**Degrees of Freedom Method**	Satterthwaite

Class Level Information		
**Class**	**Levels**	**Values**
**T**	2	A C
**S**	2	0 1
**L**	8	A1 A2 A3 A4 C1 C2 C3 C4
**Gen**	2	Y Z
**Timing**	2	Afternoon Morning

Dimensions	
**Covariance Parameters**	3
**Columns in X**	15
**Columns in Z**	24
**Subjects**	1
**Max Obs per Subject**	58

Number of Observations	
**Number of Observations Read**	58
**Number of Observations Used**	58
**Number of Observations Not Used**	0

Covariance Parameter Estimates							
**Cov Parm**	**Estimate**	**Standard Error**	**Z Value**	**Pr > Z**	**Alpha**	**Lower**	**Upper**
**L(T)**	0.05573	0.4697	0.12	0.4528	0.05	0.007551	9.13E110
**S*L(T)**	0.7483	0.6318	1.18	0.1181	0.05	0.2337	12.0137
**Residual**	1.1043	0.2517	4.39	<.0001	0.05	0.7393	1.8274

Fit Statistics	
-2 Res Log Likelihood	193.4
AIC (Smaller is Better)	199.4
AICC (Smaller is Better)	199.9
BIC (Smaller is Better)	199.7

Solution for Fixed Effects

Effect	T	Gen	Timing	S	Estimate	Standard Error	DF	t Value	Pr > |t|	Alpha	Lower	Upper
Intercept					34.5347	1.6681	48.7	20.70	<.0001	0.05	31.1821	37.8872
T	A				1.1614	0.7558	12.1	1.54	0.1500	0.05	-0.4835	2.8062
T	C				0	.	.	.	.	.	.	.
S				0	0.3483	0.7634	6.85	0.46	0.6623	0.05	-1.4647	2.1613
S				1	0	.	.	.	.	.	.	.
T*S	A			0	-0.3096	1.0398	5.9	-0.30	0.7761	0.05	-2.8640	2.2448
T*S	A			1	0	.	.	.	.	.	.	.
T*S	C			0	0	.	.	.	.	.	.	.
T*S	C			1	0	.	.	.	.	.	.	.
Gen		Y			0.3625	0.4440	43.1	0.82	0.4187	0.05	-0.5328	1.2578
Gen		Z			0	.	.	.	.	.	.	.
Timing			Afternoon		-0.01081	0.3129	43.4	-0.03	0.9726	0.05	-0.6417	0.6201
Timing			Morning		0	.	.	.	.	.	.	.
MB0					0.1430	0.06593	45.7	2.17	0.0353	0.05	0.01030	0.2758
Age					0.004173	0.007300	42	0.57	0.5706	0.05	-0.01056	0.01891

Type 3 Tests of Fixed Effects

Effect	Num DF	Den DF	F Value	Pr > F
T	1	7.29	3.03	0.1233
S	1	7.52	0.12	0.7356
T*S	1	5.9	0.09	0.7761
Gen	1	43.1	0.67	0.4187
Timing	1	43.4	0.00	0.9726
MB0	1	45.7	4.71	0.0353
Age	1	42	0.33	0.5706

Least Squares Means												
**Effect**	**T**	**Gen**	**Timing**	**S**	**Estimate**	**Standard Error**	**DF**	**t Value**	**Pr > |t|**	**Alpha**	**Lower**	**Upper**
**T**	A				40.0515	0.3945	6.51	101.52	<.0001	0.05	39.1042	40.9987
**T**	C				39.0449	0.4003	6.7	97.54	<.0001	0.05	38.0898	40.0000
**S**				0	39.6449	0.3996	14.3	99.22	<.0001	0.05	38.7896	40.5002
**S**				1	39.4514	0.3766	11.9	104.77	<.0001	0.05	38.6303	40.2725
**T*S**	A			0	40.0708	0.5482	13.3	73.09	<.0001	0.05	38.8888	41.2528
**T*S**	A			1	40.0321	0.5428	12.8	73.75	<.0001	0.05	38.8577	41.2066
**T*S**	C			0	39.2190	0.5808	15.3	67.52	<.0001	0.05	37.9831	40.4550
**T*S**	C			1	38.8707	0.5239	11.2	74.19	<.0001	0.05	37.7205	40.0209
**Gen**		Y			39.7294	0.3625	16.2	109.59	<.0001	0.05	38.9617	40.4972
**Gen**		Z			39.3669	0.3407	13.8	115.54	<.0001	0.05	38.6351	40.0987
**Timing**			Afternoon		39.5428	0.3204	10.9	123.42	<.0001	0.05	38.8364	40.2491
**Timing**			Morning		39.5536	0.3087	9.55	128.15	<.0001	0.05	38.8614	40.2457

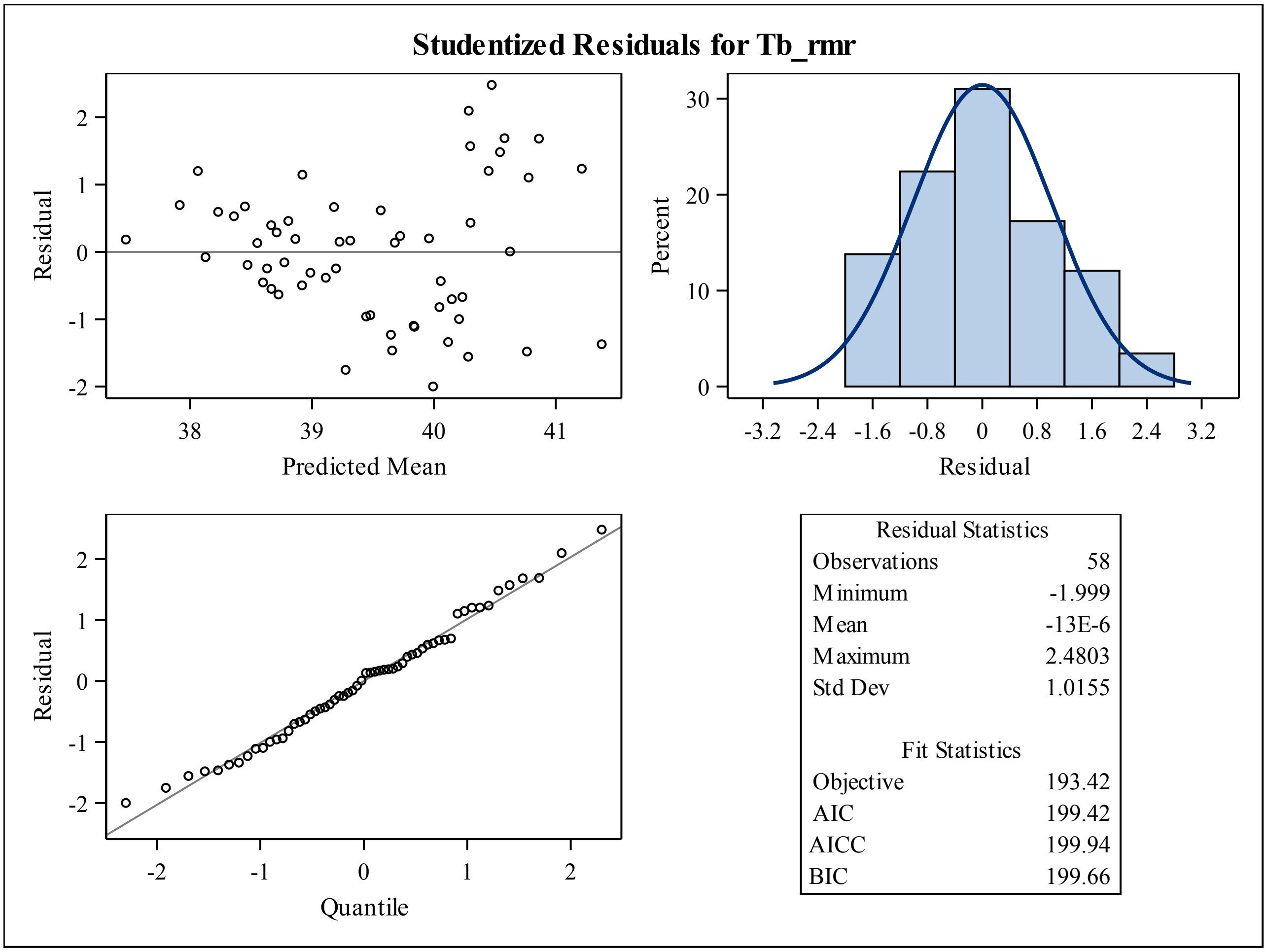

1.3. RMR: lowest resting metabolic rate

1.3.1. Output from SAS (v. 9.3) Mixed Model analysis for RMR at 10°C.

Model Information	
**Data Set**	WORK.THERMLONG
**Dependent Variable**	RMR
**Covariance Structure**	Variance Components
**Estimation Method**	REML
**Residual Variance Method**	Profile
**Fixed Effects SE Method**	Model-Based
**Degrees of Freedom Method**	Satterthwaite

Class Level Information		
**Class**	**Levels**	**Values**
**T**	2	A C
**S**	2	0 1
**L**	8	A1 A2 A3 A4 C1 C2 C3 C4
**Gen**	2	Y Z
**Timing**	2	Afternoon Morning

Dimensions	
**Covariance Parameters**	3
**Columns in X**	15
**Columns in Z**	24
**Subjects**	1
**Max Obs per Subject**	112

Number of Observations	
**Number of Observations Read**	112
**Number of Observations Used**	112
**Number of Observations Not Used**	0

Covariance Parameter Estimates							
**Cov Parm**	**Estimate**	**Standard Error**	**Z Value**	**Pr > Z**	**Alpha**	**Lower**	**Upper**
**L(T)**	0	.	.	.	.	.	.
**S*L(T)**	0	.	.	.	.	.	.
**Residual**	0.04132	0.005729	7.21	<.0001	0.05	0.03204	0.05532

Fit Statistics	
**-2 Res Log Likelihood**	1.5
**AIC (Smaller is Better)**	3.5
**AICC (Smaller is Better)**	3.5
**BIC (Smaller is Better)**	3.5

Solution for Fixed Effects												
**Effect**	**T**	**Gen**	**Timing**	**S**	**Estimate**	**Standard Error**	**DF**	**t Value**	**Pr > |t|**	**Alpha**	**Lower**	**Upper**
**Intercept**					1.6212	0.1758	104	9.22	<.0001	0.05	1.2724	1.9699
**T**	A				0.07675	0.05773	104	1.33	0.1866	0.05	-0.03772	0.1912
**T**	C				0	.	.	.	.	.	.	.
**S**				0	-0.1089	0.05828	104	-1.87	0.0645	0.05	-0.2245	0.006653
**S**				1	0	.	.	.	.	.	.	.
**T*S**	A			0	-0.02548	0.07700	104	-0.33	0.7413	0.05	-0.1782	0.1272
**T*S**	A			1	0	.	.	.	.	.	.	.
**T*S**	C			0	0	.	.	.	.	.	.	.
**T*S**	C			1	0	.	.	.	.	.	.	.
**Gen**		Y			0.003476	0.05989	104	0.06	0.9538	0.05	-0.1153	0.1222
**Gen**		Z			0	.	.	.	.	.	.	.
**Timing**			Afternoon		-0.06005	0.03878	104	-1.55	0.1245	0.05	-0.1370	0.01685
**Timing**			Morning		0	.	.	.	.	.	.	.
**MB0**					0.03446	0.006148	104	5.60	<.0001	0.05	0.02227	0.04665
**Age**					-0.00032	0.000966	104	-0.33	0.7410	0.05	-0.00224	0.001595

Type 3 Tests of Fixed Effects				
**Effect**	**Num DF**	**Den DF**	**F Value**	**Pr > F**
**T**	1	104	2.17	0.1440
**S**	1	104	7.60	0.0069
**T*S**	1	104	0.11	0.7413
**Gen**	1	104	0.00	0.9538
**Timing**	1	104	2.40	0.1245
**MB0**	1	104	31.41	<.0001
**Age**	1	104	0.11	0.7410

Least Squares Means												
Effect	T	Gen	Timing	S	Estimate	Standard Error	DF	t Value	Pr > |t|	Alpha	Lower	Upper
T	A				2.4191	0.02963	104	81.65	<.0001	0.05	2.3603	2.4778
T	C				2.3551	0.02855	104	82.49	<.0001	0.05	2.2985	2.4117
S				0	2.3263	0.02952	104	78.81	<.0001	0.05	2.2677	2.3848
S				1	2.4479	0.02916	104	83.96	<.0001	0.05	2.3901	2.5057
T*S	A			0	2.3519	0.03971	104	59.22	<.0001	0.05	2.2731	2.4306
T*S	A			1	2.4863	0.04370	104	56.89	<.0001	0.05	2.3996	2.5729
T*S	C			0	2.3006	0.04327	104	53.17	<.0001	0.05	2.2148	2.3864
T*S	C			1	2.4095	0.03816	104	63.14	<.0001	0.05	2.3339	2.4852
Gen		Y			2.3888	0.03724	104	64.14	<.0001	0.05	2.3150	2.4627
Gen		Z			2.3853	0.03397	104	70.22	<.0001	0.05	2.3180	2.4527
Timing			Afternoon		2.3571	0.02771	104	85.06	<.0001	0.05	2.3021	2.4120
Timing			Morning		2.4171	0.02705	104	89.35	<.0001	0.05	2.3635	2.4708

Studentized Residuals for RMR

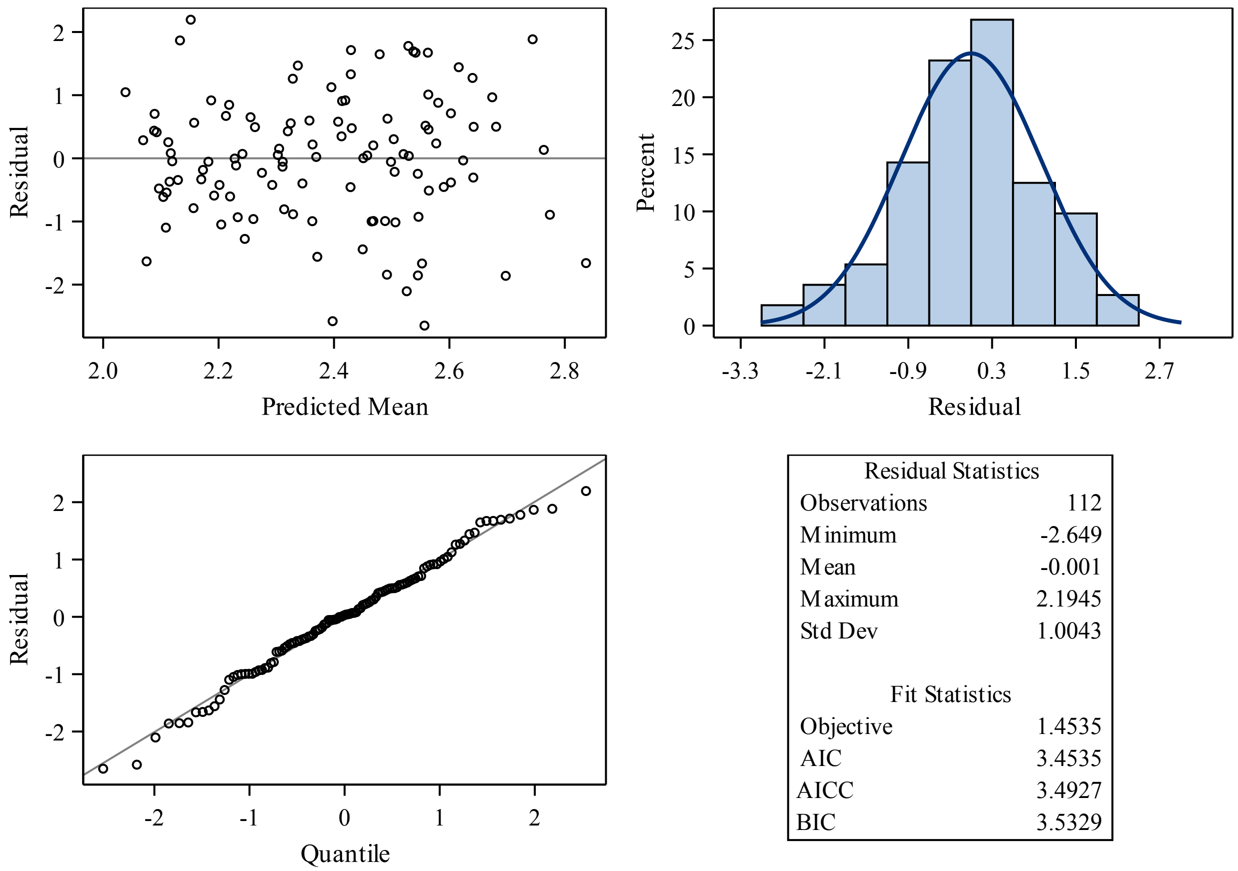

Residual Statistics	
Observations	112
Minimum	-2.649
Mean	-0.001
Maximum	2.1945
Std Dev	1.0043
**Fit Statistics**	
Objective	1.4535
AIC	3.4535
AICC	3.4927
BIC	3.5329

1.3.2. Output from SAS (v. 9.3) Mixed Model analysis for RMR at 20°C.

Model Information	
**Data Set**	WORK.THERMLONG
**Dependent Variable**	RMR
**Covariance Structure**	Variance Components
**Estimation Method**	REML
**Residual Variance Method**	Profile
**Fixed Effects SE Method**	Model-Based
**Degrees of Freedom Method**	Satterthwaite

Class Level Information		
**Class**	**Levels**	**Values**
**T**	2	A C
**S**	2	0 1
**L**	8	A1 A2 A3 A4 C1 C2 C3 C4
**Gen**	2	Y Z
**Timing**	2	Afternoon Morning

Dimensions	
**Covariance Parameters**	3
**Columns in X**	15
**Columns in Z**	24
**Subjects**	1
**Max Obs per Subject**	137

Number of Observations	
**Number of Observations Read**	137
**Number of Observations Used**	137
**Number of Observations Not Used**	0

Covariance Parameter Estimates							
**Cov Parm**	**Estimate**	**Standard Error**	**Z Value**	**Pr > Z**	**Alpha**	**Lower**	**Upper**
**L(T)**	0	.	.	.	.	.	.
**S*L(T)**	0	.	.	.	.	.	.
**Residual**	0.02631	0.003276	8.03	<.0001	0.05	0.02091	0.03413

Fit Statistics	
-2 Res Log Likelihood	-63.7
AIC (Smaller is Better)	-61.7
AICC (Smaller is Better)	-61.7
BIC (Smaller is Better)	-61.7

Solution for Fixed Effects												
Effect	T	Gen	Timing	S	Estimate	Standard Error	DF	t Value	Pr > |t|	Alpha	Lower	Upper
Intercept					0.4922	0.1272	129	3.87	0.0002	0.05	0.2406	0.7439
T	A				0.02610	0.04167	129	0.63	0.5321	0.05	-0.05634	0.1085
T	C				0	.	.	.	.	.	.	.
S				0	-0.01964	0.04174	129	-0.47	0.6387	0.05	-0.1022	0.06294
S				1	0	.	.	.	.	.	.	.
T*S	A			0	0.01671	0.05570	129	0.30	0.7647	0.05	-0.09349	0.1269
T*S	A			1	0	.	.	.	.	.	.	.
T*S	C			0	0	.	.	.	.	.	.	.
T*S	C			1	0	.	.	.	.	.	.	.
Gen		Y			-0.1410	0.03950	129	-3.57	0.0005	0.05	-0.2191	-0.06282
Gen		Z			0	.	.	.	.	.	.	.
Timing			Afternoon		-0.04485	0.02784	129	-1.61	0.1096	0.05	-0.09994	0.01024
Timing			Morning		0	.	.	.	.	.	.	.
MB0					0.04073	0.004572	129	8.91	<.0001	0.05	0.03169	0.04978
Age					0.001415	0.000636	129	2.23	0.0278	0.05	0.000157	0.002673

Type 3 Tests of Fixed Effects				
Effect	Num DF	Den DF	F Value	Pr > F
T	1	129	1.21	0.2724
S	1	129	0.13	0.7222
T*S	1	129	0.09	0.7647
Gen	1	129	12.74	0.0005
Timing	1	129	2.60	0.1096
MB0	1	129	79.36	<.0001
Age	1	129	4.95	0.0278

Least Squares Means												
**Effect**	**T**	**Gen**	**Timing**	**S**	**Estimate**	**Standard Error**	**DF**	**t Value**	**Pr > |t|**	**Alpha**	**Lower**	**Upper**
**T**	A				1.6403	0.02094	129	78.35	<.0001	0.05	1.5989	1.6818
**T**	C				1.6059	0.02101	129	76.45	<.0001	0.05	1.5643	1.6474
**S**				0	1.6175	0.02118	129	76.37	<.0001	0.05	1.5756	1.6594
**S**				1	1.6287	0.02107	129	77.29	<.0001	0.05	1.5871	1.6704
**T*S**	A			0	1.6389	0.02823	129	58.05	<.0001	0.05	1.5830	1.6947
**T*S**	A			1	1.6418	0.03143	129	52.24	<.0001	0.05	1.5796	1.7040
**T*S**	C			0	1.5961	0.03139	129	50.85	<.0001	0.05	1.5340	1.6582
**T*S**	C			1	1.6157	0.02772	129	58.28	<.0001	0.05	1.5608	1.6705
**Gen**		Y			1.5526	0.02301	129	67.49	<.0001	0.05	1.5071	1.5981
**Gen**		Z			1.6936	0.02533	129	66.85	<.0001	0.05	1.6435	1.7437
**Timing**			Afternoon		1.6007	0.01961	129	81.61	<.0001	0.05	1.5619	1.6395
**Timing**			Morning		1.6455	0.01984	129	82.93	<.0001	0.05	1.6063	1.6848

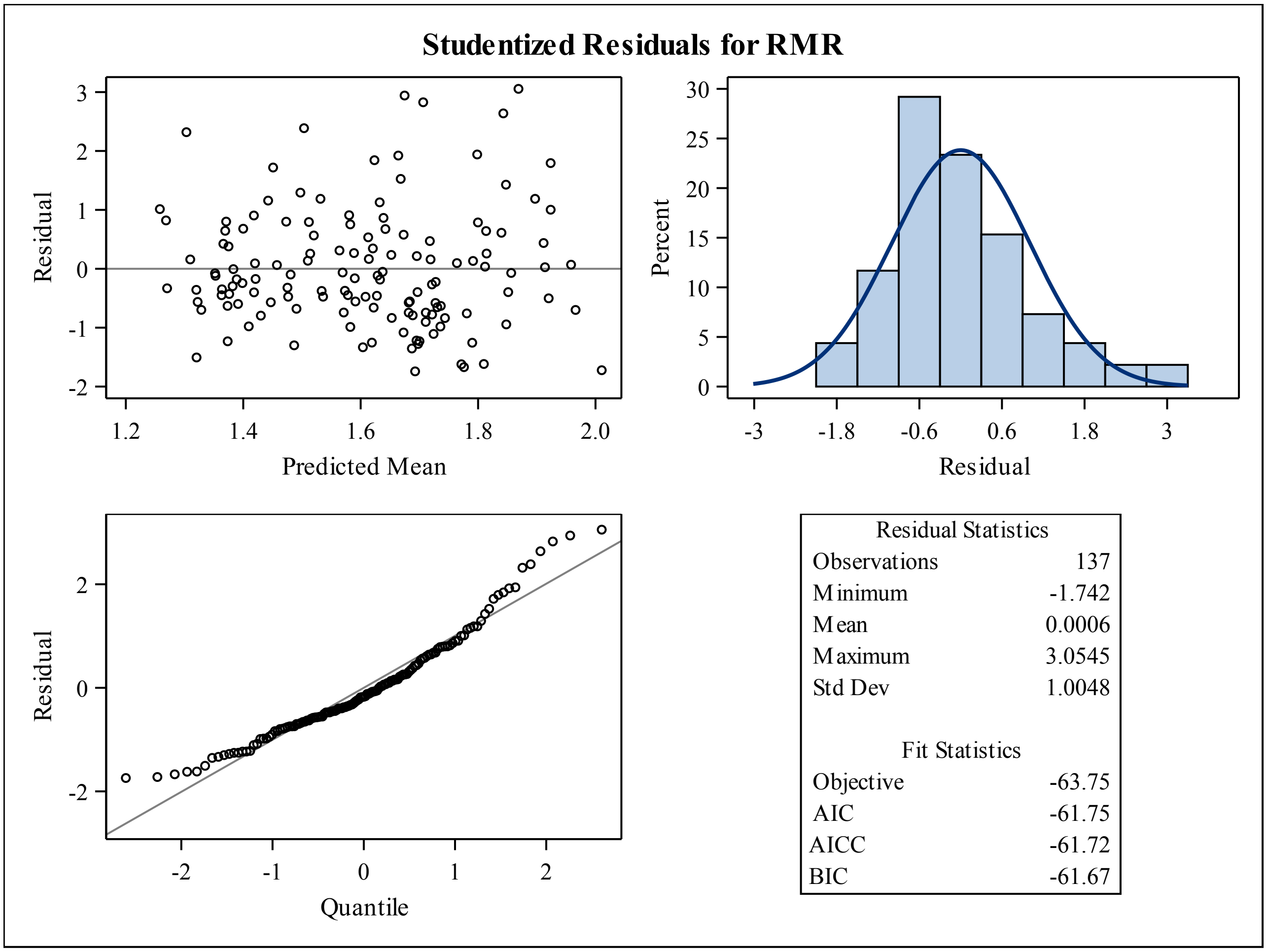

1.3.3. Output from SAS (v. 9.3) Mixed Model analysis for RMR at 25°C.

Model Information	
Data Set	WORK.THERMLONG
Dependent Variable	RMR
Covariance Structure	Variance Components
Estimation Method	REML
Residual Variance Method	Profile
Fixed Effects SE Method	Model-Based
Degrees of Freedom Method	Satterthwaite

Class Level Information		
Class	Levels	Values
T	2	A C
S	2	0 1
L	8	A1 A2 A3 A4 C1 C2 C3 C4
Gen	2	Y Z
Timing	2	Afternoon Morning

Dimensions	
Covariance Parameters	3
Columns in X	15
Columns in Z	24
Subjects	1
Max Obs per Subject	60

Number of Observations	
Number of Observations Read	60
Number of Observations Used	60
Number of Observations Not Used	0

Covariance Parameter Estimates							
Cov Parm	Estimate	Standard Error	Z Value	Pr > Z	Alpha	Lower	Upper
L(T)	0	.	.	.	.	.	.
S*L(T)	0	.	.	.	.	.	.
Residual	0.01396	0.002738	5.10	<.0001	0.05	0.009837	0.02137

Fit Statistics	
**-2 Res Log Likelihood**	-42.2
**AIC (Smaller is Better)**	-40.2
**AICC (Smaller is Better)**	-40.1
**BIC (Smaller is Better)**	-40.1

Solution for Fixed Effects												
**Effect**	**T**	**Gen**	**Timing**	**S**	**Estimate**	**Standard Error**	**DF**	**t Value**	**Pr > |t|**	**Alpha**	**Lower**	**Upper**
**Intercept**					0.5164	0.1507	52	3.43	0.0012	0.05	0.2140	0.8189
**T**	A				0.05789	0.04367	52	1.33	0.1908	0.05	-0.02974	0.1455
**T**	C				0	.	.	.	.	.	.	.
**S**				0	-0.03761	0.04868	52	-0.77	0.4433	0.05	-0.1353	0.06008
**S**				1	0	.	.	.	.	.	.	.
**T*S**	A			0	0.1080	0.06289	52	1.72	0.0920	0.05	-0.01822	0.2342
**T*S**	A			1	0	.	.	.	.	.	.	.
**T*S**	C			0	0	.	.	.	.	.	.	.
**T*S**	C			1	0	.	.	.	.	.	.	.
**Gen**		Y			0.002306	0.04580	52	0.05	0.9600	0.05	-0.08960	0.09422
**Gen**		Z			0	.	.	.	.	.	.	.
**Timing**			Afternoon		-0.01784	0.03133	52	-0.57	0.5715	0.05	-0.08070	0.04502
**Timing**			Morning		0	.	.	.	.	.	.	.
**MB0**					0.02993	0.006018	52	4.97	<.0001	0.05	0.01785	0.04201
**Age**					-0.00063	0.000764	52	-0.82	0.4165	0.05	-0.00216	0.000908

Type 3 Tests of Fixed Effects				
**Effect**	**Num DF**	**Den DF**	**F Value**	**Pr > F**
**T**	1	52	10.52	0.0021
**S**	1	52	0.21	0.6525
**T*S**	1	52	2.95	0.0920
**Gen**	1	52	0.00	0.9600
**Timing**	1	52	0.32	0.5715
**MB0**	1	52	24.73	<.0001
**Age**	1	52	0.67	0.4165

Least Squares Means												
**Effect**	**T**	**Gen**	**Timing**	**S**	**Estimate**	**Standard Error**	**DF**	**t Value**	**Pr > |t|**	**Alpha**	**Lower**	**Upper**
**T**	A				1.2623	0.02361	52	53.48	<.0001	0.05	1.2150	1.3097
**T**	C				1.1505	0.02302	52	49.97	<.0001	0.05	1.1043	1.1967
**S**				0	1.2146	0.02558	52	47.48	<.0001	0.05	1.1633	1.2659
**S**				1	1.1982	0.02218	52	54.03	<.0001	0.05	1.1537	1.2427
**T*S**	A			0	1.2975	0.03319	52	39.10	<.0001	0.05	1.2309	1.3641
**T*S**	A			1	1.2272	0.03354	52	36.59	<.0001	0.05	1.1599	1.2945
**T*S**	C			0	1.1317	0.03785	52	29.90	<.0001	0.05	1.0557	1.2076
**T*S**	C			1	1.1693	0.02850	52	41.03	<.0001	0.05	1.1121	1.2265
**Gen**		Y			1.2076	0.02945	52	41.00	<.0001	0.05	1.1485	1.2667
**Gen**		Z			1.2053	0.02596	52	46.44	<.0001	0.05	1.1532	1.2573
**Timing**			Afternoon		1.1975	0.02167	52	55.25	<.0001	0.05	1.1540	1.2410
**Timing**			Morning		1.2153	0.02265	52	53.65	<.0001	0.05	1.1699	1.2608

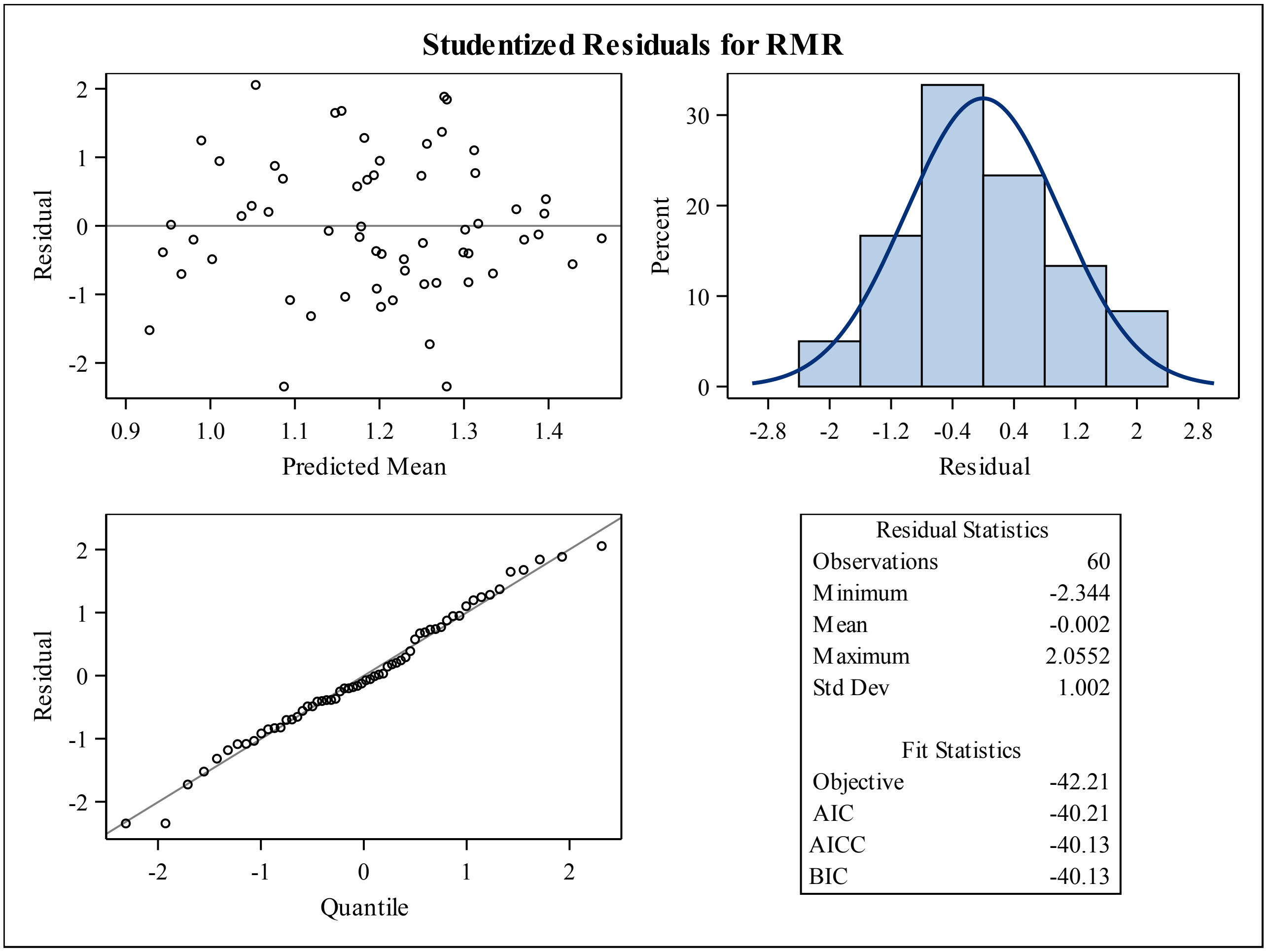

1.3.4. Output from SAS (v. 9.3) Mixed Model analysis for RMR at 28°C.

Model Information	
Data Set	WORK.THERMLONG
Dependent Variable	RMR
Covariance Structure	Variance Components
Estimation Method	REML
Residual Variance Method	Profile
Fixed Effects SE Method	Model-Based
Degrees of Freedom Method	Satterthwaite

Class Level Information		
Class	Levels	Values
T	2	A C
S	2	0 1
L	8	A1 A2 A3 A4 C1 C2 C3 C4
Gen	2	Y Z
Timing	2	Afternoon Morning

Dimensions	
Covariance Parameters	3
Columns in X	15
Columns in Z	24
Subjects	1
Max Obs per Subject	61

Number of Observations	
Number of Observations Read	61
Number of Observations Used	61
Number of Observations Not Used	0

Covariance Parameter Estimates							
Cov Parm	Estimate	Standard Error	Z Value	Pr > Z	Alpha	Lower	Upper
L(T)	0.003190	0.003152	1.01	0.1558	0.05	0.000874	0.1169
S*L(T)	0	.	.	.	.	.	.
Residual	0.01644	0.003379	4.87	<.0001	0.05	0.01141	0.02575

Fit Statistics	
-2 Res Log Likelihood	-29.7
AIC (Smaller is Better)	-25.7
AICC (Smaller is Better)	-25.5
BIC (Smaller is Better)	-25.5

Solution for Fixed Effects												
Effect	T	Gen	Timing	S	Estimate	Standard Error	DF	t Value	Pr > |t|	Alpha	Lower	Upper
Intercept					0.3983	0.1782	51.4	2.24	0.0298	0.05	0.04063	0.7560
T	A				0.08877	0.06244	11.8	1.42	0.1810	0.05	-0.04750	0.2250
T	C				0	.	.	.	.	.	.	.
S				0	-0.01072	0.05202	52.3	-0.21	0.8375	0.05	-0.1151	0.09364
S				1	0	.	.	.	.	.	.	.
T*S	A			0	0.03716	0.06816	49.5	0.55	0.5881	0.05	-0.09978	0.1741
T*S	A			1	0	.	.	.	.	.	.	.
T*S	C			0	0	.	.	.	.	.	.	.
T*S	C			1	0	.	.	.	.	.	.	.
Gen		Y			0.04721	0.05122	49.7	0.92	0.3611	0.05	-0.05568	0.1501
Gen		Z			0	.	.	.	.	.	.	.
Timing			Afternoon		-0.00324	0.03511	50.1	-0.09	0.9269	0.05	-0.07376	0.06728
Timing			Morning		0	.	.	.	.	.	.	.
MB0					0.03181	0.007112	51.5	4.47	<.0001	0.05	0.01754	0.04609
Age					-0.00091	0.000839	50.1	-1.08	0.2850	0.05	-0.00259	0.000778

Type 3 Tests of Fixed Effects				
Effect	Num DF	Den DF	F Value	Pr > F
T	1	7.39	3.77	0.0912
S	1	52.1	0.04	0.8448
T*S	1	49.5	0.30	0.5881
Gen	1	49.7	0.85	0.3611
Timing	1	50.1	0.01	0.9269
MB0	1	51.5	20.01	<.0001
Age	1	50.1	1.17	0.2850

Least Squares Means												
**Effect**	**T**	**Gen**	**Timing**	**S**	**Estimate**	**Standard Error**	**DF**	**t Value**	**Pr > |t|**	**Alpha**	**Lower**	**Upper**
**T**	A				1.1906	0.03853	7.14	30.90	<.0001	0.05	1.0998	1.2814
**T**	C				1.0833	0.03754	6.3	28.86	<.0001	0.05	0.9925	1.1740
**S**				0	1.1409	0.03419	15	33.37	<.0001	0.05	1.0680	1.2137
**S**				1	1.1330	0.03151	12	35.95	<.0001	0.05	1.0643	1.2017
**T*S**	A			0	1.2038	0.04737	15.2	25.41	<.0001	0.05	1.1030	1.3047
**T*S**	A			1	1.1774	0.04614	13.6	25.52	<.0001	0.05	1.0781	1.2767
**T*S**	C			0	1.0779	0.04862	14.6	22.17	<.0001	0.05	0.9740	1.1818
**T*S**	C			1	1.0886	0.04250	10.3	25.61	<.0001	0.05	0.9943	1.1829
**Gen**		Y			1.1605	0.03848	22.5	30.16	<.0001	0.05	1.0808	1.2402
**Gen**		Z			1.1133	0.03458	17	32.20	<.0001	0.05	1.0404	1.1863
**Timing**			Afternoon		1.1353	0.03218	12.9	35.28	<.0001	0.05	1.0657	1.2049
**Timing**			Morning		1.1386	0.03075	11.3	37.03	<.0001	0.05	1.0711	1.2060

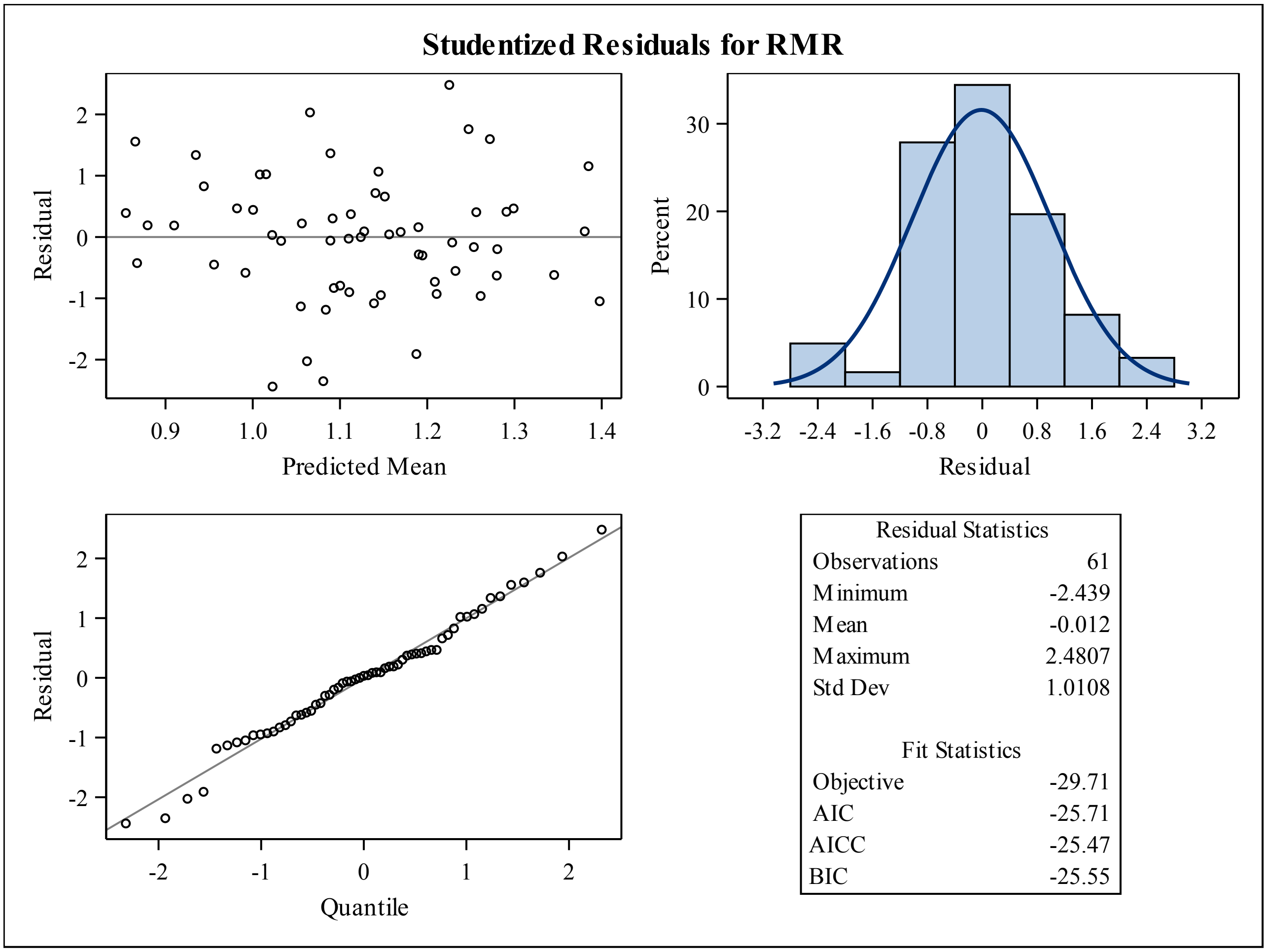

1.3.5. Output from SAS (v. 9.3) Mixed Model analysis for RMR at 31°C.

Model Information	
Data Set	WORK.THERMLONG
Dependent Variable	RMR
Covariance Structure	Variance Components
Estimation Method	REML
Residual Variance Method	Profile
Fixed Effects SE Method	Model-Based
Degrees of Freedom Method	Satterthwaite

Class Level Information		
Class	Levels	Values
T	2	A C
S	2	0 1
L	8	A1 A2 A3 A4 C1 C2 C3 C4
Gen	2	Y Z
Timing	2	Afternoon Morning

Dimensions	
Covariance Parameters	3
Columns in X	15
Columns in Z	24
Subjects	1
Max Obs per Subject	58

Number of Observations	
Number of Observations Read	58
Number of Observations Used	58
Number of Observations Not Used	0

Covariance Parameter Estimates							
Cov Parm	Estimate	Standard Error	Z Value	Pr > Z	Alpha	Lower	Upper
L(T)	0.003710	0.004822	0.77	0.2208	0.05	0.000797	1.3512
S*L(T)	0	.	.	.	.	.	.
Residual	0.03112	0.006606	4.71	<.0001	0.05	0.02136	0.04954

Fit Statistics	
**-2 Res Log Likelihood**	3.8
**AIC (Smaller is Better)**	7.8
**AICC (Smaller is Better)**	8.1
**BIC (Smaller is Better)**	8.0

Solution for Fixed Effects

Effect	T	Gen	Timing	S	Estimate	Standard Error	DF	t Value	Pr > |t|	Alpha	Lower	Upper
**Intercept**					-0.1859	0.2435	47.8	-0.76	0.4488	0.05	-0.6755	0.3037
**T**	A				0.1225	0.08007	13.5	1.53	0.1491	0.05	-0.04984	0.2949
**T**	C				0	.	.	.	.	.	.	.
**S**				0	0.1158	0.06986	49.3	1.66	0.1037	0.05	-0.02456	0.2562
**S**				1	0	.	.	.	.	.	.	.
**T*S**	A			0	-0.03959	0.09760	46.2	-0.41	0.6868	0.05	-0.2360	0.1568
**T*S**	A			1	0	.	.	.	.	.	.	.
**T*S**	C			0	0	.	.	.	.	.	.	.
**T*S**	C			1	0	.	.	.	.	.	.	.
**Gen**		Y			0.06592	0.07170	46.4	0.92	0.3626	0.05	-0.07837	0.2102
**Gen**		Z			0	.	.	.	.	.	.	.
**Timing**			Afternoon		0.05389	0.04973	47.3	1.08	0.2841	0.05	-0.04614	0.1539
**Timing**			Morning		0	.	.	.	.	.	.	.
**MB0**					0.04996	0.009794	47.2	5.10	<.0001	0.05	0.03026	0.06966
**Age**					-0.00069	0.001201	46.6	-0.57	0.5704	0.05	-0.00310	0.001730

Type 3 Tests of Fixed Effects

Effect	Num DF	Den DF	F Value	Pr > F
**T**	1	7.41	2.24	0.1757
**S**	1	48.9	3.01	0.0891
**T*S**	1	46.2	0.16	0.6868
**Gen**	1	46.4	0.85	0.3626
**Timing**	1	47.3	1.17	0.2841
**MB0**	1	47.2	26.02	<.0001
**Age**	1	46.6	0.33	0.5704

Least Squares Means												
**Effect**	**T**	**Gen**	**Timing**	**S**	**Estimate**	**Standard Error**	**DF**	**t Value**	**Pr > |t|**	**Alpha**	**Lower**	**Upper**
**T**	A				1.1876	0.04888	7.89	24.30	<.0001	0.05	1.0746	1.3006
**T**	C				1.0848	0.04533	5.74	23.93	<.0001	0.05	0.9727	1.1970
**S**				0	1.1842	0.04464	17.8	26.53	<.0001	0.05	1.0904	1.2781
**S**				1	1.0882	0.04036	13.5	26.96	<.0001	0.05	1.0013	1.1751
**T*S**	A			0	1.2257	0.06440	20.7	19.03	<.0001	0.05	1.0916	1.3597
**T*S**	A			1	1.1495	0.06029	16.2	19.06	<.0001	0.05	1.0218	1.2772
**T*S**	C			0	1.1427	0.06101	14.8	18.73	<.0001	0.05	1.0125	1.2730
**T*S**	C			1	1.0269	0.05318	10.8	19.31	<.0001	0.05	0.9097	1.1442
**Gen**		Y			1.1692	0.05103	27	22.91	<.0001	0.05	1.0645	1.2739
**Gen**		Z			1.1033	0.04535	20.5	24.33	<.0001	0.05	1.0088	1.1977
**Timing**			Afternoon		1.1632	0.04226	15.6	27.53	<.0001	0.05	1.0734	1.2529
**Timing**			Morning		1.1093	0.03926	12.8	28.26	<.0001	0.05	1.0243	1.1942

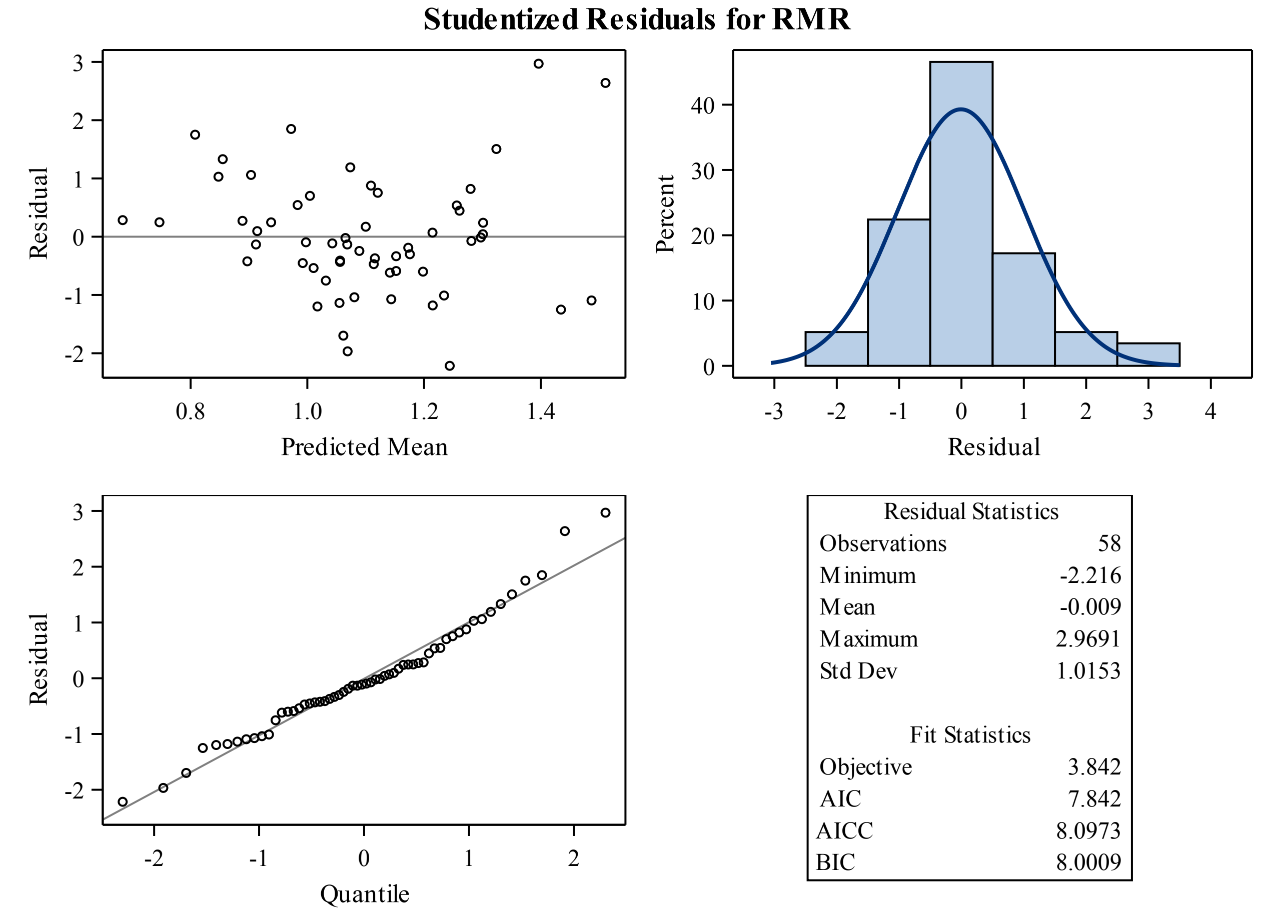

Residual Statistics	
Observations	58
Minimum	-2.216
Mean	-0.009
Maximum	2.9691
Std Dev	1.0153

Fit Statistics	
Objective	3.842
AIC	7.842
AICC	8.0973
BIC	8.0009

1.3.6. Output from SAS (v. 9.3) Mixed Model analysis for RMR at 34°C.

Model Information	
Data Set	WORK.THERMLONG
Dependent Variable	RMR
Covariance Structure	Variance Components
Estimation Method	REML
Residual Variance Method	Profile
Fixed Effects SE Method	Model-Based
Degrees of Freedom Method	Satterthwaite

Class Level Information		
Class	Levels	Values
T	2	A C
S	2	0 1
L	8	A1 A2 A3 A4 C1 C2 C3 C4
Gen	2	Y Z
Timing	2	Afternoon Morning

Dimensions	
Covariance Parameters	3
Columns in X	15
Columns in Z	23
Subjects	1
Max Obs per Subject	50

Number of Observations	
Number of Observations Read	50
Number of Observations Used	50
Number of Observations Not Used	0

Covariance Parameter Estimates							
Cov Parm	Estimate	Standard Error	Z Value	Pr > Z	Alpha	Lower	Upper
L(T)	0	.	.	.	.	.	.
S*L(T)	0.01859	0.01188	1.57	0.0588	0.05	0.007192	0.1149
Residual	0.02680	0.006725	3.98	<.0001	0.05	0.01731	0.04700

Fit Statistics	
-2 Res Log Likelihood	9.8
AIC (Smaller is Better)	13.8
AICC (Smaller is Better)	14.1
BIC (Smaller is Better)	13.9

Solution for Fixed Effects												
Effect	T	Gen	Timing	S	Estimate	Standard Error	DF	t Value	Pr > |t|	Alpha	Lower	Upper
Intercept					0.05819	0.2979	41.9	0.20	0.8461	0.05	-0.5431	0.6595
T	A				0.08262	0.1256	10.5	0.66	0.5247	0.05	-0.1954	0.3606
T	C				0	.	.	.	.	.	.	.
S				0	0.01761	0.1215	11.9	0.14	0.8872	0.05	-0.2474	0.2826
S				1	0	.	.	.	.	.	.	.
T*S	A			0	-0.05915	0.1726	10.8	-0.34	0.7385	0.05	-0.4401	0.3218
T*S	A			1	0	.	.	.	.	.	.	.
T*S	C			0	0	.	.	.	.	.	.	.
T*S	C			1	0	.	.	.	.	.	.	.
Gen		Y			0.08235	0.07917	35.4	1.04	0.3053	0.05	-0.07830	0.2430
Gen		Z			0	.	.	.	.	.	.	.
Timing			Afternoon		-0.00697	0.05380	37.2	-0.13	0.8976	0.05	-0.1160	0.1020
Timing			Morning		0	.	.	.	.	.	.	.
MB0					0.05011	0.01112	41.8	4.51	<.0001	0.05	0.02767	0.07255
Age					-0.00113	0.001303	34.5	-0.87	0.3911	0.05	-0.00378	0.001515

Type 3 Tests of Fixed Effects				
Effect	Num DF	Den DF	F Value	Pr > F
T	1	12.6	0.34	0.5686
S	1	13.5	0.02	0.9002
T*S	1	10.8	0.12	0.7385
Gen	1	35.4	1.08	0.3053
Timing	1	37.2	0.02	0.8976
MB0	1	41.8	20.31	<.0001
Age	1	34.5	0.75	0.3911

Least Squares Means												
**Effect**	**T**	**Gen**	**Timing**	**S**	**Estimate**	**Standard Error**	**DF**	**t Value**	**Pr > |t|**	**Alpha**	**Lower**	**Upper**
**T**	A				1.2519	0.06618	12.5	18.92	<.0001	0.05	1.1084	1.3954
**T**	C				1.1988	0.05972	11.2	20.07	<.0001	0.05	1.0677	1.3300
**S**				0	1.2194	0.06516	14.5	18.71	<.0001	0.05	1.0801	1.3587
**S**				1	1.2314	0.06307	10.6	19.52	<.0001	0.05	1.0919	1.3708
**T*S**	A			0	1.2311	0.09050	14	13.60	<.0001	0.05	1.0371	1.4252
**T*S**	A			1	1.2727	0.09701	11.3	13.12	<.0001	0.05	1.0597	1.4856
**T*S**	C			0	1.2077	0.08990	13.5	13.43	<.0001	0.05	1.0141	1.4012
**T*S**	C			1	1.1900	0.08018	9.61	14.84	<.0001	0.05	1.0104	1.3697
**Gen**		Y			1.2665	0.06271	31.4	20.20	<.0001	0.05	1.1387	1.3944
**Gen**		Z			1.1842	0.05514	23.5	21.47	<.0001	0.05	1.0703	1.2981
**Timing**			Afternoon		1.2219	0.05391	21.9	22.67	<.0001	0.05	1.1101	1.3337
**Timing**			Morning		1.2289	0.04880	16.1	25.18	<.0001	0.05	1.1254	1.3323

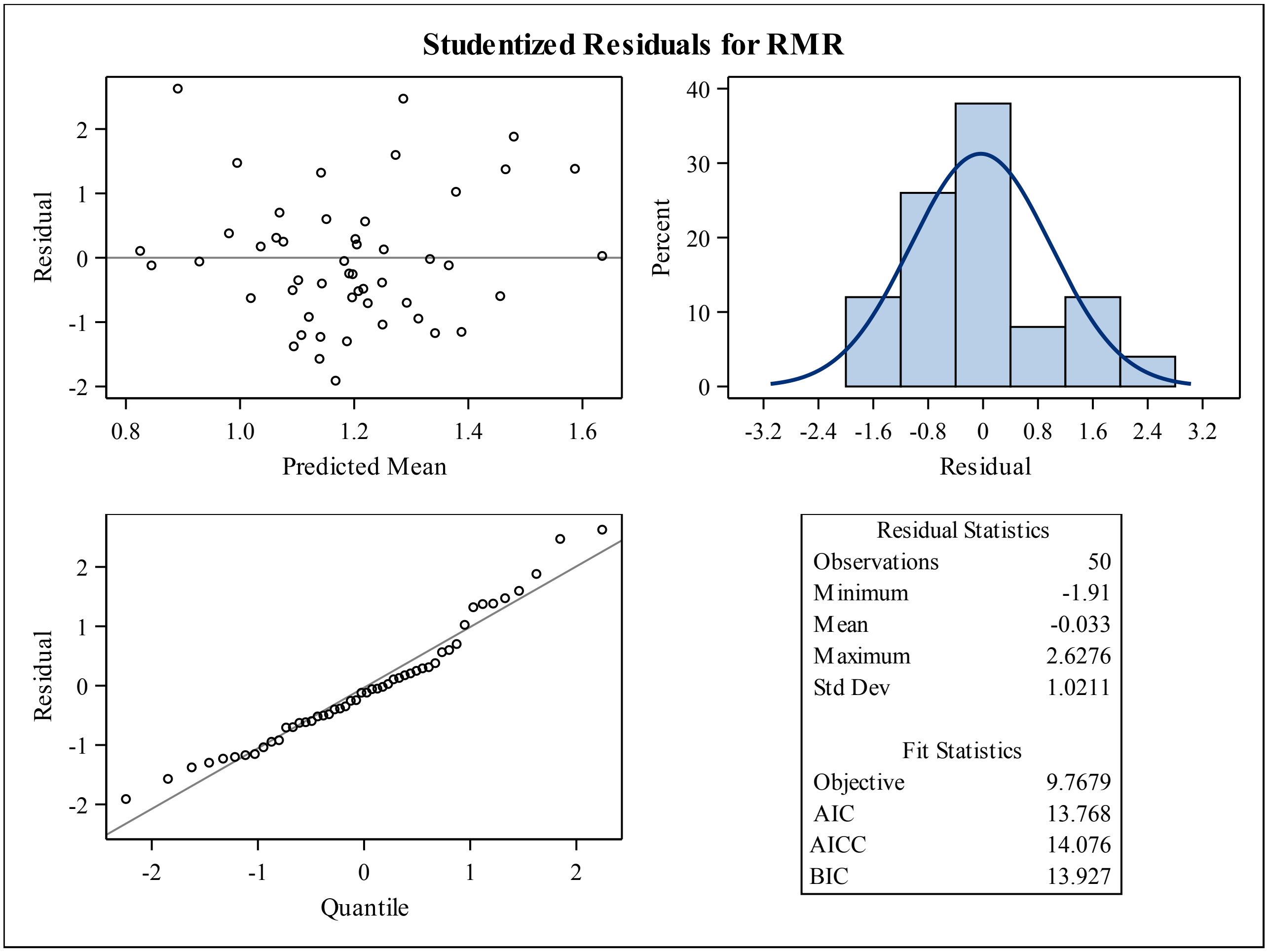

1.4. CT: thermal conductance (estimated for a particular temperature)

1.4.1. Output from SAS (v. 9.3) Mixed Model analysis for CT at 10°C.

Model Information	
Data Set	WORK.THERMLONG
Dependent Variable	CT
Covariance Structure	Variance Components
Estimation Method	REML
Residual Variance Method	Profile
Fixed Effects SE Method	Model-Based
Degrees of Freedom Method	Satterthwaite

Class Level Information		
Class	Levels	Values
T	2	A C
S	2	0 1
L	8	A1 A2 A3 A4 C1 C2 C3 C4
Gen	2	Y Z
Timing	2	Afternoon Morning

Dimensions	
Covariance Parameters	3
Columns in X	15
Columns in Z	24
Subjects	1
Max Obs per Subject	105

Number of Observations	
Number of Observations Read	105
Number of Observations Used	105
Number of Observations Not Used	0

Covariance Parameter Estimates							
Cov Parm	Estimate	Standard Error	Z Value	Pr > Z	Alpha	Lower	Upper
L(T)	0	.	.	.	.	.	.
S*L(T)	0	.	.	.	.	.	.
Residual	0.000058	8.331E-6	6.96	<.0001	0.05	0.000045	0.000079

Fit Statistics	
**-2 Res Log Likelihood**	-633.9
**AIC (Smaller is Better)**	-631.9
**AICC (Smaller is Better)**	-631.9
**BIC (Smaller is Better)**	-631.9

Solution for Fixed Effects												
**Effect**	**T**	**Gen**	**Timing**	**S**	**Estimate**	**Standard Error**	**DF**	**t Value**	**Pr > |t|**	**Alpha**	**Lower**	**Upper**
**Intercept**					0.05214	0.007016	97	7.43	<.0001	0.05	0.03821	0.06606
**T**	A				0.001781	0.002298	97	0.77	0.4403	0.05	-0.00278	0.006342
**T**	C				0	.	.	.	.	.	.	.
**S**				0	-0.00225	0.002222	97	-1.01	0.3137	0.05	-0.00666	0.002160
**S**				1	0	.	.	.	.	.	.	.
**T*S**	A			0	-0.00146	0.002990	97	-0.49	0.6266	0.05	-0.00739	0.004475
**T*S**	A			1	0	.	.	.	.	.	.	.
**T*S**	C			0	0	.	.	.	.	.	.	.
**T*S**	C			1	0	.	.	.	.	.	.	.
**Gen**		Y			0.000711	0.002367	97	0.30	0.7644	0.05	-0.00399	0.005409
**Gen**		Z			0	.	.	.	.	.	.	.
**Timing**			Afternoon		-0.00142	0.001522	97	-0.93	0.3527	0.05	-0.00444	0.001600
**Timing**			Morning		0	.	.	.	.	.	.	.
**MB0**					0.001513	0.000258	97	5.86	<.0001	0.05	0.001000	0.002025
**Age**					-0.00003	0.000038	97	-0.78	0.4382	0.05	-0.00011	0.000046

Type 3 Tests of Fixed Effects				
**Effect**	**Num DF**	**Den DF**	**F Value**	**Pr > F**
**T**	1	97	0.38	0.5373
**S**	1	97	3.03	0.0850
**T*S**	1	97	0.24	0.6266
**Gen**	1	97	0.09	0.7644
**Timing**	1	97	0.87	0.3527
**MB0**	1	97	34.35	<.0001
**Age**	1	97	0.61	0.4382

Least Squares Means												
**Effect**	**T**	**Gen**	**Timing**	**S**	**Estimate**	**Standard Error**	**DF**	**t Value**	**Pr > |t|**	**Alpha**	**Lower**	**Upper**
**T**	A				0.08537	0.001137	97	75.08	<.0001	0.05	0.08311	0.08763
**T**	C				0.08432	0.001129	97	74.66	<.0001	0.05	0.08208	0.08656
**S**				0	0.08335	0.001154	97	72.22	<.0001	0.05	0.08106	0.08564
**S**				1	0.08633	0.001123	97	76.86	<.0001	0.05	0.08410	0.08856
**T*S**	A			0	0.08351	0.001543	97	54.13	<.0001	0.05	0.08045	0.08658
**T*S**	A			1	0.08722	0.001704	97	51.17	<.0001	0.05	0.08384	0.09061
**T*S**	C			0	0.08319	0.001661	97	50.08	<.0001	0.05	0.07990	0.08649
**T*S**	C			1	0.08544	0.001503	97	56.84	<.0001	0.05	0.08246	0.08843
**Gen**		Y			0.08520	0.001471	97	57.92	<.0001	0.05	0.08228	0.08812
**Gen**		Z			0.08449	0.001328	97	63.61	<.0001	0.05	0.08185	0.08712
**Timing**			Afternoon		0.08413	0.001076	97	78.22	<.0001	0.05	0.08200	0.08627
**Timing**			Morning		0.08555	0.001063	97	80.50	<.0001	0.05	0.08344	0.08766

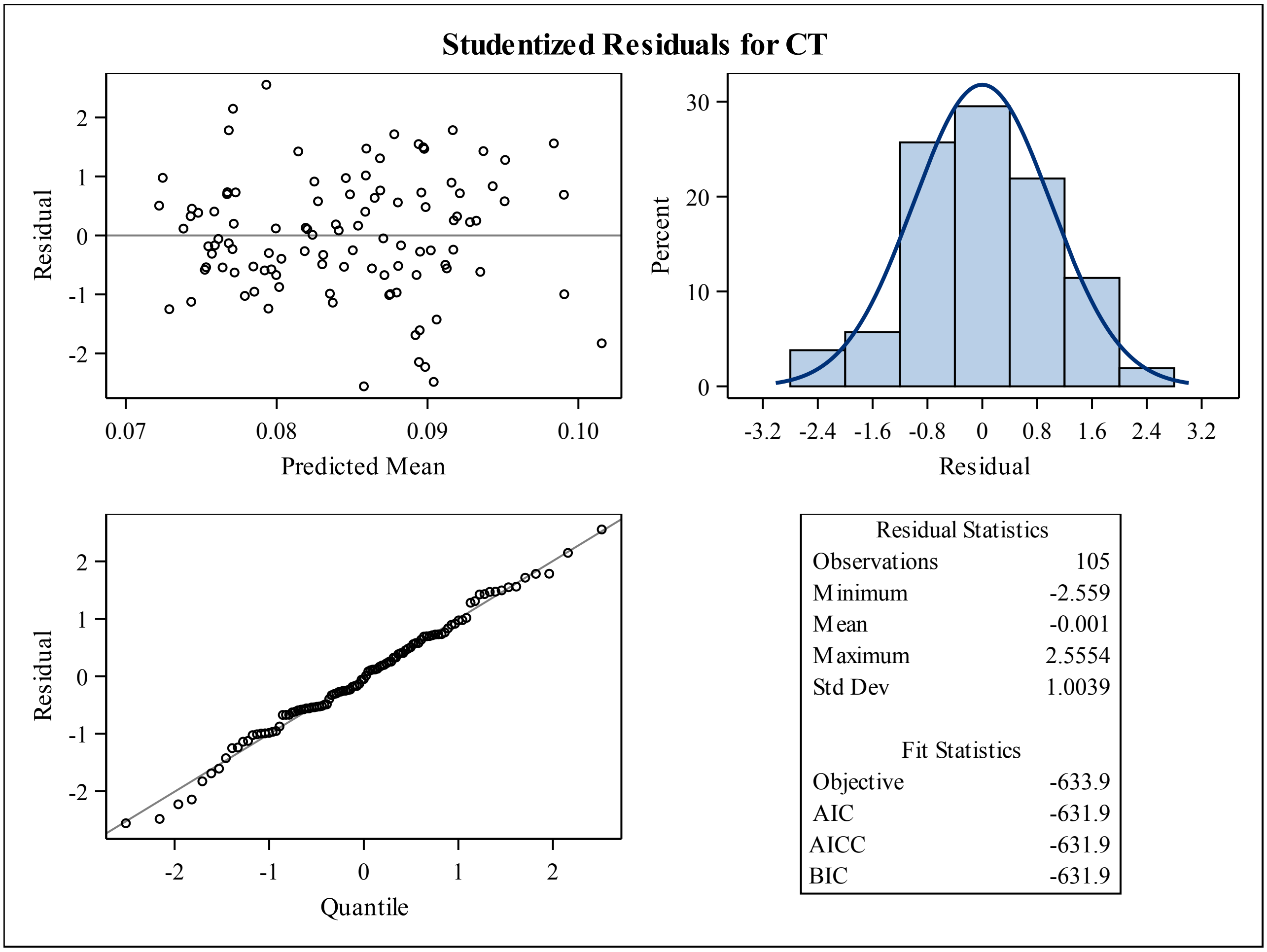

1.4.2. Output from SAS (v. 9.3) Mixed Model analysis for CT at 20°C.

Model Information	
**Data Set**	WORK.THERMLONG
**Dependent Variable**	CT
**Covariance Structure**	Variance Components
**Estimation Method**	REML
**Residual Variance Method**	Profile
**Fixed Effects SE Method**	Model-Based
**Degrees of Freedom Method**	Satterthwaite

Class Level Information		
**Class**	**Levels**	**Values**
**T**	2	A C
**S**	2	0 1
**L**	8	A1 A2 A3 A4 C1 C2 C3 C4
**Gen**	2	Y Z
**Timing**	2	Afternoon Morning

Dimensions	
**Covariance Parameters**	3
**Columns in X**	15
**Columns in Z**	24
**Subjects**	1
**Max Obs per Subject**	127

Number of Observations	
**Number of Observations Read**	127
**Number of Observations Used**	127
**Number of Observations Not Used**	0

Covariance Parameter Estimates							
**Cov Parm**	**Estimate**	**Standard Error**	**Z Value**	**Pr > Z**	**Alpha**	**Lower**	**Upper**
**L(T)**	0	.	.	.	.	.	.
**S*L(T)**	0	.	.	.	.	.	.
**Residual**	0.000072	9.293E-6	7.71	<.0001	0.05	0.000056	0.000094

Fit Statistics	
-2 Res Log Likelihood	-759.2
AIC (Smaller is Better)	-757.2
AICC (Smaller is Better)	-757.1
BIC (Smaller is Better)	-757.1

Solution for Fixed Effects												
Effect	T	Gen	Timing	S	Estimate	Standard Error	DF	t Value	Pr > |t|	Alpha	Lower	Upper
Intercept					0.02050	0.006953	119	2.95	0.0038	0.05	0.006731	0.03427
T	A				0.002518	0.002295	119	1.10	0.2747	0.05	-0.00203	0.007062
T	C				0	.	.	.	.	.	.	.
S				0	-0.00016	0.002246	119	-0.07	0.9421	0.05	-0.00461	0.004283
S				1	0	.	.	.	.	.	.	.
T*S	A			0	-0.00061	0.003021	119	-0.20	0.8412	0.05	-0.00659	0.005375
T*S	A			1	0	.	.	.	.	.	.	.
T*S	C			0	0	.	.	.	.	.	.	.
T*S	C			1	0	.	.	.	.	.	.	.
Gen		Y			-0.00694	0.002148	119	-3.23	0.0016	0.05	-0.01120	-0.00269
Gen		Z			0	.	.	.	.	.	.	.
Timing			Afternoon		-0.00210	0.001520	119	-1.38	0.1694	0.05	-0.00511	0.000908
Timing			Morning		0	.	.	.	.	.	.	.
MB0					0.002619	0.000255	119	10.26	<.0001	0.05	0.002114	0.003125
Age					0.000057	0.000034	119	1.65	0.1019	0.05	-0.00001	0.000124

Type 3 Tests of Fixed Effects				
Effect	Num DF	Den DF	F Value	Pr > F
T	1	119	1.73	0.1915
S	1	119	0.07	0.7855
T*S	1	119	0.04	0.8412
Gen	1	119	10.44	0.0016
Timing	1	119	1.91	0.1694
MB0	1	119	105.30	<.0001
Age	1	119	2.72	0.1019

Least Squares Means												
**Effect**	**T**	**Gen**	**Timing**	**S**	**Estimate**	**Standard Error**	**DF**	**t Value**	**Pr > |t|**	**Alpha**	**Lower**	**Upper**
**T**	A				0.09151	0.001119	119	81.76	<.0001	0.05	0.08930	0.09373
**T**	C				0.08930	0.001151	119	77.57	<.0001	0.05	0.08702	0.09158
**S**				0	0.09017	0.001148	119	78.56	<.0001	0.05	0.08790	0.09244
**S**				1	0.09064	0.001142	119	79.39	<.0001	0.05	0.08838	0.09290
**T*S**	A			0	0.09113	0.001521	119	59.91	<.0001	0.05	0.08811	0.09414
**T*S**	A			1	0.09190	0.001697	119	54.16	<.0001	0.05	0.08854	0.09526
**T*S**	C			0	0.08921	0.001677	119	53.20	<.0001	0.05	0.08589	0.09254
**T*S**	C			1	0.08938	0.001536	119	58.18	<.0001	0.05	0.08634	0.09242
**Gen**		Y			0.08693	0.001246	119	69.79	<.0001	0.05	0.08447	0.08940
**Gen**		Z			0.09387	0.001383	119	67.87	<.0001	0.05	0.09114	0.09661
**Timing**			Afternoon		0.08935	0.001069	119	83.57	<.0001	0.05	0.08724	0.09147
**Timing**			Morning		0.09145	0.001081	119	84.61	<.0001	0.05	0.08931	0.09359

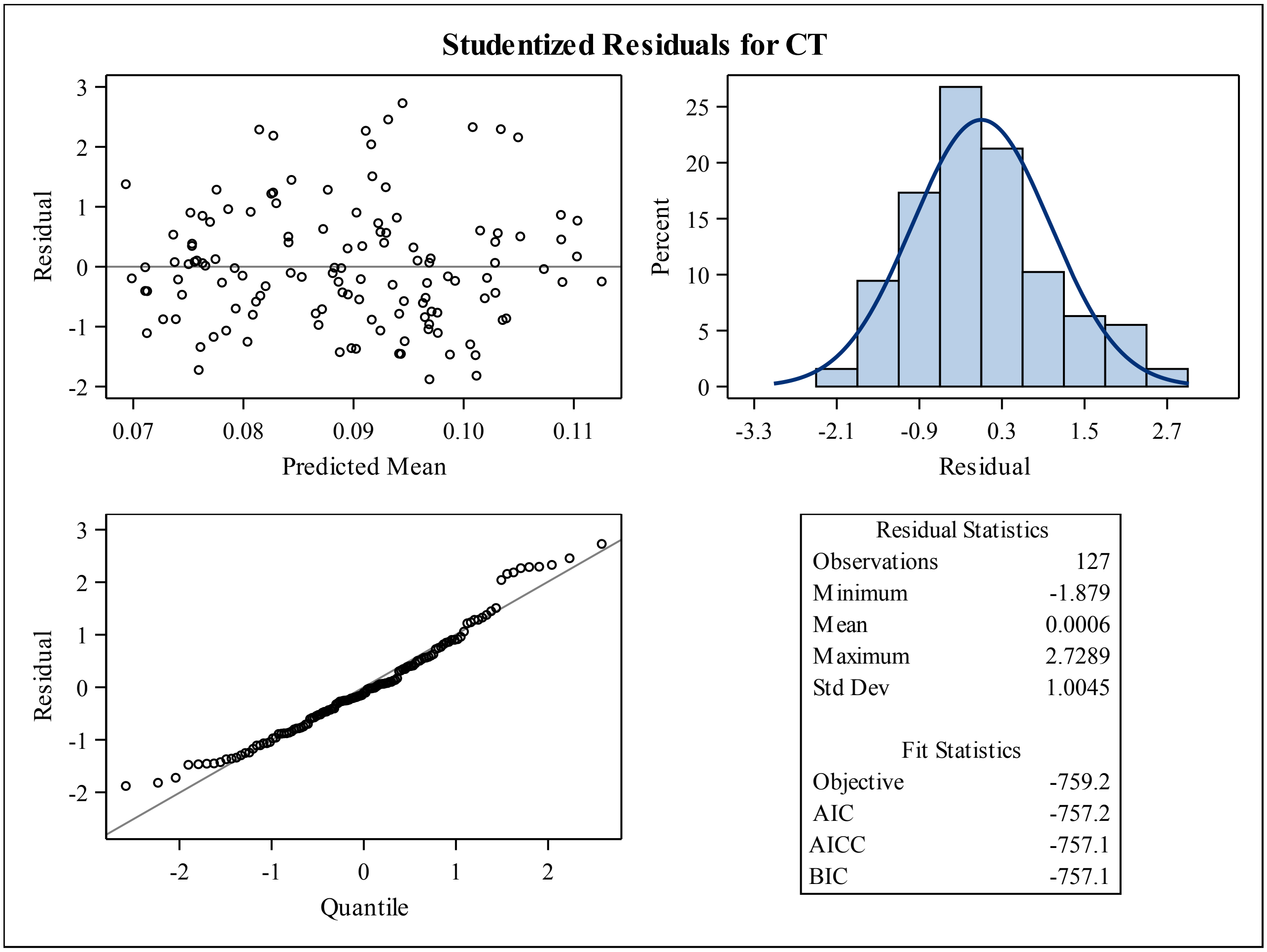

1.4.3. Output from SAS (v. 9.3) Mixed Model analysis for CT at 25°C.

Model Information	
**Data Set**	WORK.THERMLONG
**Dependent Variable**	CT
**Covariance Structure**	Variance Components
**Estimation Method**	REML
**Residual Variance Method**	Profile
**Fixed Effects SE Method**	Model-Based
**Degrees of Freedom Method**	Satterthwaite

Class Level Information		
**Class**	**Levels**	**Values**
**T**	2	A C
**S**	2	0 1
**L**	8	A1 A2 A3 A4 C1 C2 C3 C4
**Gen**	2	Y Z
**Timing**	2	Afternoon Morning

Dimensions	
**Covariance Parameters**	3
**Columns in X**	15
**Columns in Z**	24
**Subjects**	1
**Max Obs per Subject**	57

Number of Observations	
**Number of Observations Read**	57
**Number of Observations Used**	57
**Number of Observations Not Used**	0

Covariance Parameter Estimates							
**Cov Parm**	**Estimate**	**Standard Error**	**Z Value**	**Pr > Z**	**Alpha**	**Lower**	**Upper**
**L(T)**	1.25E-6	5.666E-6	0.22	0.4127	0.05	1.099E-7	8.76E25
**S*L(T)**	0	.	.	.	.	.	.
**Residual**	0.000056	0.000012	4.66	<.0001	0.05	0.000038	0.000090

Fit Statistics	
**-2 Res Log Likelihood**	-307.8
**AIC (Smaller is Better)**	-303.8
**AICC (Smaller is Better)**	-303.5
**BIC (Smaller is Better)**	-303.6

Solution for Fixed Effects

Effect	T	Gen	Timing	S	Estimate	Standard Error	DF	t Value	Pr > |t|	Alpha	Lower	Upper
**Intercept**					0.03237	0.01002	44.9	3.23	0.0023	0.05	0.01218	0.05256
**T**	A				0.001751	0.002961	17.5	0.59	0.5618	0.05	-0.00448	0.007984
**T**	C				0	.	.	.	.	.	.	.
**S**				0	-0.00122	0.003128	49	-0.39	0.6985	0.05	-0.00751	0.005068
**S**				1	0	.	.	.	.	.	.	.
**T*S**	A			0	0.007400	0.004085	47.3	1.81	0.0765	0.05	-0.00082	0.01562
**T*S**	A			1	0	.	.	.	.	.	.	.
**T*S**	C			0	0	.	.	.	.	.	.	.
**T*S**	C			1	0	.	.	.	.	.	.	.
**Gen**		Y			0.000880	0.002939	46.2	0.30	0.7659	0.05	-0.00503	0.006795
**Gen**		Z			0	.	.	.	.	.	.	.
**Timing**			Afternoon		-0.00022	0.002073	47.8	-0.11	0.9162	0.05	-0.00439	0.003949
**Timing**			Morning		0	.	.	.	.	.	.	.
**MB0**					0.002583	0.000419	46.2	6.17	<.0001	0.05	0.001740	0.003425
**Age**					-0.00005	0.000050	46.2	-0.91	0.3671	0.05	-0.00015	0.000055

Type 3 Tests of Fixed Effects

Effect	Num DF	Den DF	F Value	Pr > F
**T**	1	8.51	5.05	0.0529
**S**	1	49	1.14	0.2909
**T*S**	1	47.3	3.28	0.0765
**Gen**	1	46.2	0.09	0.7659
**Timing**	1	47.8	0.01	0.9162
**MB0**	1	46.2	38.05	<.0001
**Age**	1	46.2	0.83	0.3671

Least Squares Means												
**Effect**	**T**	**Gen**	**Timing**	**S**	**Estimate**	**Standard Error**	**DF**	**t Value**	**Pr > |t|**	**Alpha**	**Lower**	**Upper**
**T**	A				0.09575	0.001632	7.49	58.66	<.0001	0.05	0.09194	0.09956
**T**	C				0.09030	0.001640	7.21	55.04	<.0001	0.05	0.08644	0.09415
**S**				0	0.09426	0.001713	22.3	55.04	<.0001	0.05	0.09071	0.09781
**S**				1	0.09178	0.001476	16.3	62.17	<.0001	0.05	0.08866	0.09491
**T*S**	A			0	0.09884	0.002250	20.9	43.94	<.0001	0.05	0.09416	0.1035
**T*S**	A			1	0.09266	0.002224	20.1	41.66	<.0001	0.05	0.08802	0.09730
**T*S**	C			0	0.08969	0.002546	23.6	35.23	<.0001	0.05	0.08443	0.09495
**T*S**	C			1	0.09091	0.001948	13.7	46.67	<.0001	0.05	0.08672	0.09509
**Gen**		Y			0.09346	0.001917	32.6	48.76	<.0001	0.05	0.08956	0.09736
**Gen**		Z			0.09258	0.001748	27.9	52.95	<.0001	0.05	0.08900	0.09616
**Timing**			Afternoon		0.09291	0.001439	15.5	64.58	<.0001	0.05	0.08986	0.09597
**Timing**			Morning		0.09313	0.001578	20.4	59.00	<.0001	0.05	0.08984	0.09642

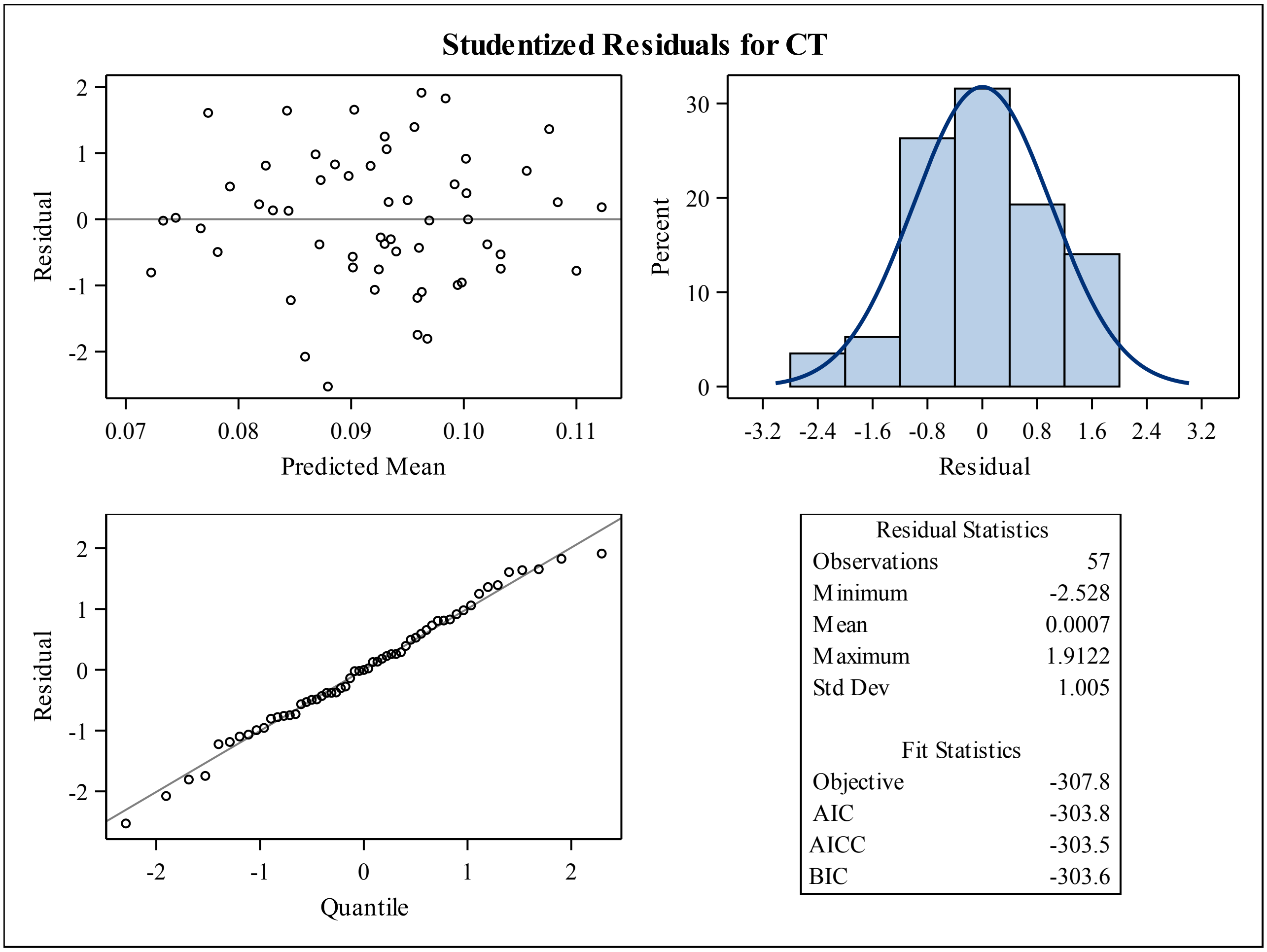

1.4.4. Output from SAS (v. 9.3) Mixed Model analysis for CT at 28°C.

Model Information	
**Data Set**	WORK.THERMLONG
**Dependent Variable**	CT
**Covariance Structure**	Variance Components
**Estimation Method**	REML
**Residual Variance Method**	Profile
**Fixed Effects SE Method**	Model-Based
**Degrees of Freedom Method**	Satterthwaite

Class Level Information		
**Class**	**Levels**	**Values**
**T**	2	A C
**S**	2	0 1
**L**	8	A1 A2 A3 A4 C1 C2 C3 C4
**Gen**	2	Y Z
**Timing**	2	Afternoon Morning

Dimensions	
**Covariance Parameters**	3
**Columns in X**	15
**Columns in Z**	24
**Subjects**	1
**Max Obs per Subject**	57

Number of Observations	
**Number of Observations Read**	57
**Number of Observations Used**	57
**Number of Observations Not Used**	0

Covariance Parameter Estimates							
**Cov Parm**	**Estimate**	**Standard Error**	**Z Value**	**Pr > Z**	**Alpha**	**Lower**	**Upper**
**L(T)**	8.805E-7	0.000012	0.08	0.4701	0.05	7.77E-7	6.37E275
**S*L(T)**	0	.	.	.	.	.	.
**Residual**	0.000122	0.000026	4.65	<.0001	0.05	0.000084	0.000196

Fit Statistics	
-2 Res Log Likelihood	-270.2
AIC (Smaller is Better)	-266.2
AICC (Smaller is Better)	-265.9
BIC (Smaller is Better)	-266.0

Solution for Fixed Effects												
Effect	T	Gen	Timing	S	Estimate	Standard Error	DF	t Value	Pr > |t|	Alpha	Lower	Upper
Intercept					0.03097	0.01475	44.5	2.10	0.0414	0.05	0.001266	0.06068
T	A				0.005765	0.004301	18.1	1.34	0.1967	0.05	-0.00327	0.01480
T	C				0	.	.	.	.	.	.	.
S				0	0.000514	0.004405	48.4	0.12	0.9076	0.05	-0.00834	0.009369
S				1	0	.	.	.	.	.	.	.
T*S	A			0	0.002064	0.005993	48	0.34	0.7321	0.05	-0.00999	0.01411
T*S	A			1	0	.	.	.	.	.	.	.
T*S	C			0	0	.	.	.	.	.	.	.
T*S	C			1	0	.	.	.	.	.	.	.
Gen		Y			0.005514	0.004396	47.2	1.25	0.2159	0.05	-0.00333	0.01436
Gen		Z			0	.	.	.	.	.	.	.
Timing			Afternoon		0.005064	0.003082	48.5	1.64	0.1069	0.05	-0.00113	0.01126
Timing			Morning		0	.	.	.	.	.	.	.
MB0					0.003529	0.000600	41.8	5.88	<.0001	0.05	0.002318	0.004739
Age					-0.00010	0.000074	48	-1.29	0.2020	0.05	-0.00024	0.000053

Type 3 Tests of Fixed Effects				
Effect	Num DF	Den DF	F Value	Pr > F
T	1	8.84	3.75	0.0853
S	1	48.9	0.21	0.6470
T*S	1	48	0.12	0.7321
Gen	1	47.2	1.57	0.2159
Timing	1	48.5	2.70	0.1069
MB0	1	41.8	34.61	<.0001
Age	1	48	1.67	0.2020

Least Squares Means												
**Effect**	**T**	**Gen**	**Timing**	**S**	**Estimate**	**Standard Error**	**DF**	**t Value**	**Pr > |t|**	**Alpha**	**Lower**	**Upper**
**T**	A				0.1181	0.002355	7.71	50.15	<.0001	0.05	0.1126	0.1236
**T**	C				0.1113	0.002294	6.49	48.53	<.0001	0.05	0.1058	0.1168
**S**				0	0.1155	0.002414	19.8	47.85	<.0001	0.05	0.1104	0.1205
**S**				1	0.1139	0.002111	16.1	53.98	<.0001	0.05	0.1095	0.1184
**T*S**	A			0	0.1194	0.003398	24.1	35.14	<.0001	0.05	0.1124	0.1264
**T*S**	A			1	0.1168	0.003175	20	36.79	<.0001	0.05	0.1102	0.1234
**T*S**	C			0	0.1116	0.003485	19	32.01	<.0001	0.05	0.1043	0.1189
**T*S**	C			1	0.1111	0.002842	14.1	39.07	<.0001	0.05	0.1050	0.1172
**Gen**		Y			0.1175	0.002813	31.6	41.75	<.0001	0.05	0.1117	0.1232
**Gen**		Z			0.1120	0.002530	28.6	44.26	<.0001	0.05	0.1068	0.1171
**Timing**			Afternoon		0.1172	0.002194	17.2	53.44	<.0001	0.05	0.1126	0.1219
**Timing**			Morning		0.1122	0.002142	17.6	52.36	<.0001	0.05	0.1077	0.1167

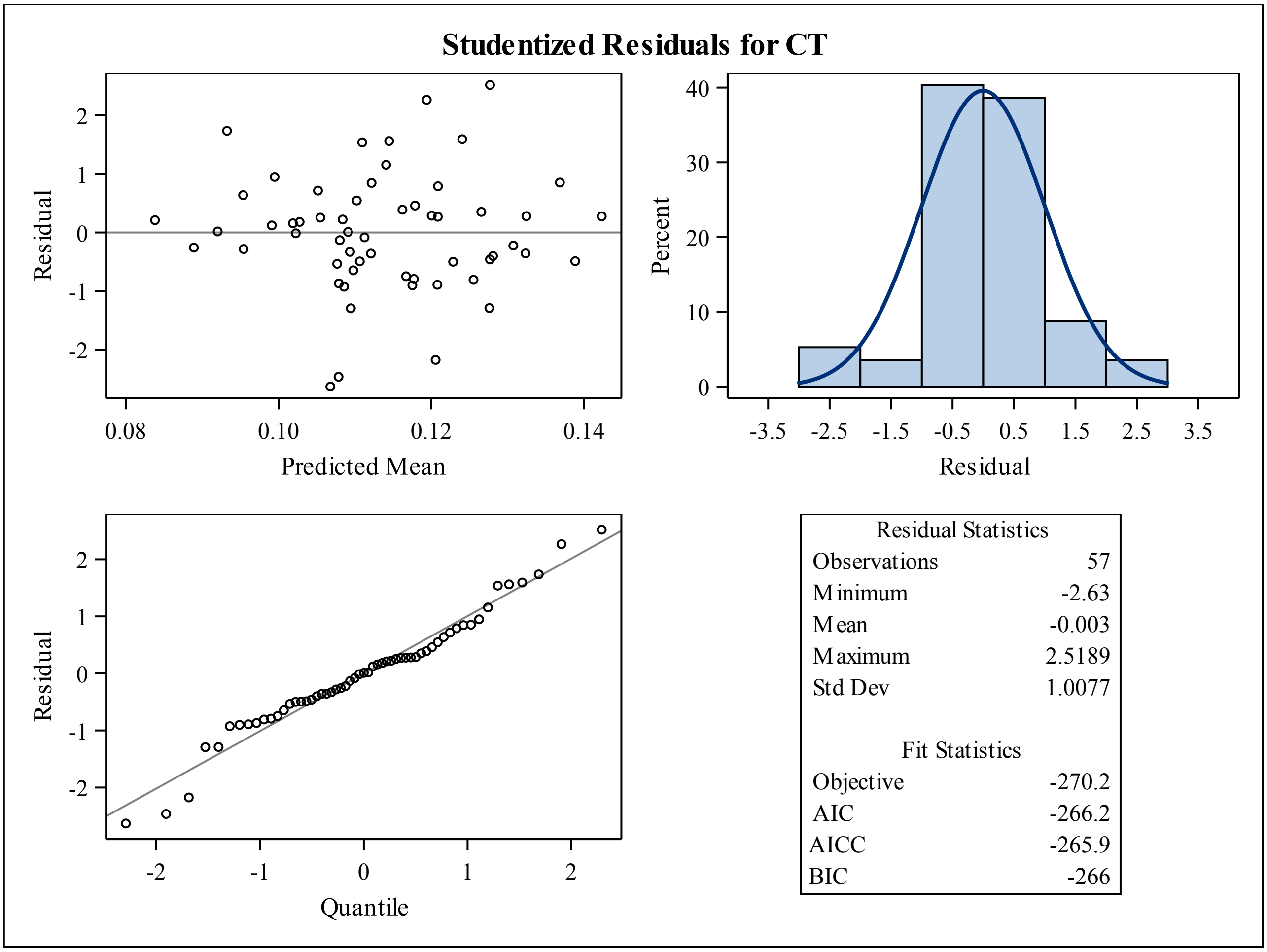

1.4.5. Output from SAS (v. 9.3) Mixed Model analysis for CT at 31°C.

Model Information	
**Data Set**	WORK.THERMLONG
**Dependent Variable**	CT
**Covariance Structure**	Variance Components
**Estimation Method**	REML
**Residual Variance Method**	Profile
**Fixed Effects SE Method**	Model-Based
**Degrees of Freedom Method**	Satterthwaite

Class Level Information		
**Class**	**Levels**	**Values**
**T**	2	A C
**S**	2	0 1
**L**	8	A1 A2 A3 A4 C1 C2 C3 C4
**Gen**	2	Y Z
**Timing**	2	Afternoon Morning

Dimensions	
**Covariance Parameters**	3
**Columns in X**	15
**Columns in Z**	24
**Subjects**	1
**Max Obs per Subject**	54

Number of Observations	
**Number of Observations Read**	54
**Number of Observations Used**	54
**Number of Observations Not Used**	0

Covariance Parameter Estimates							
**Cov Parm**	**Estimate**	**Standard Error**	**Z Value**	**Pr > Z**	**Alpha**	**Lower**	**Upper**
**L(T)**	0.000020	0.000039	0.52	0.3024	0.05	3.065E-6	7.6631
**S*L(T)**	0	.	.	.	.	.	.
**Residual**	0.000312	0.000069	4.51	<.0001	0.05	0.000211	0.000507

Fit Statistics	
**-2 Res Log Likelihood**	-207.6
**AIC (Smaller is Better)**	-203.6
**AICC (Smaller is Better)**	-203.3
**BIC (Smaller is Better)**	-203.4

Solution for Fixed Effects

Effect	T	Gen	Timing	S	Estimate	Standard Error	DF	t Value	Pr > |t|	Alpha	Lower	Upper
**Intercept**					-0.00629	0.02496	44.7	-0.25	0.8024	0.05	-0.05657	0.04400
**T**	A				0.01161	0.007701	16.2	1.51	0.1508	0.05	-0.00470	0.02793
**T**	C				0	.	.	.	.	.	.	.
**S**				0	0.008473	0.006993	45.9	1.21	0.2319	0.05	-0.00560	0.02255
**S**				1	0	.	.	.	.	.	.	.
**T*S**	A			0	-0.00567	0.01005	43.8	-0.56	0.5755	0.05	-0.02594	0.01459
**T*S**	A			1	0	.	.	.	.	.	.	.
**T*S**	C			0	0	.	.	.	.	.	.	.
**T*S**	C			1	0	.	.	.	.	.	.	.
**Gen**		Y			0.005901	0.007392	42.3	0.80	0.4292	0.05	-0.00901	0.02082
**Gen**		Z			0	.	.	.	.	.	.	.
**Timing**			Afternoon		0.01079	0.005226	43.3	2.06	0.0450	0.05	0.000254	0.02133
**Timing**			Morning		0	.	.	.	.	.	.	.
**MB0**					0.006148	0.001063	45.5	5.79	<.0001	0.05	0.004009	0.008288
**Age**					-0.00005	0.000126	42.2	-0.43	0.6682	0.05	-0.00031	0.000200

Type 3 Tests of Fixed Effects

Effect	Num DF	Den DF	F Value	Pr > F
**T**	1	8.45	1.87	0.2064
**S**	1	45.5	1.04	0.3128
**T*S**	1	43.8	0.32	0.5755
**Gen**	1	42.3	0.64	0.4292
**Timing**	1	43.3	4.26	0.0450
**MB0**	1	45.5	33.47	<.0001
**Age**	1	42.2	0.19	0.6682

Least Squares Means												
**Effect**	**T**	**Gen**	**Timing**	**S**	**Estimate**	**Standard Error**	**DF**	**t Value**	**Pr > |t|**	**Alpha**	**Lower**	**Upper**
**T**	A				0.1612	0.004566	9.11	35.29	<.0001	0.05	0.1508	0.1715
**T**	C				0.1524	0.004181	6.18	36.45	<.0001	0.05	0.1422	0.1625
**S**				0	0.1596	0.004299	20.4	37.12	<.0001	0.05	0.1506	0.1685
**S**				1	0.1540	0.003810	15.2	40.40	<.0001	0.05	0.1458	0.1621
**T*S**	A			0	0.1626	0.006297	25.2	25.81	<.0001	0.05	0.1496	0.1755
**T*S**	A			1	0.1598	0.005776	18.7	27.66	<.0001	0.05	0.1477	0.1719
**T*S**	C			0	0.1566	0.005837	16.3	26.83	<.0001	0.05	0.1443	0.1690
**T*S**	C			1	0.1481	0.005033	12.5	29.43	<.0001	0.05	0.1372	0.1591
**Gen**		Y			0.1597	0.005048	31.8	31.64	<.0001	0.05	0.1494	0.1700
**Gen**		Z			0.1538	0.004427	25.2	34.74	<.0001	0.05	0.1447	0.1629
**Timing**			Afternoon		0.1622	0.004094	19.5	39.61	<.0001	0.05	0.1536	0.1707
**Timing**			Morning		0.1514	0.003827	16.1	39.56	<.0001	0.05	0.1433	0.1595

Studentized Residuals for CT

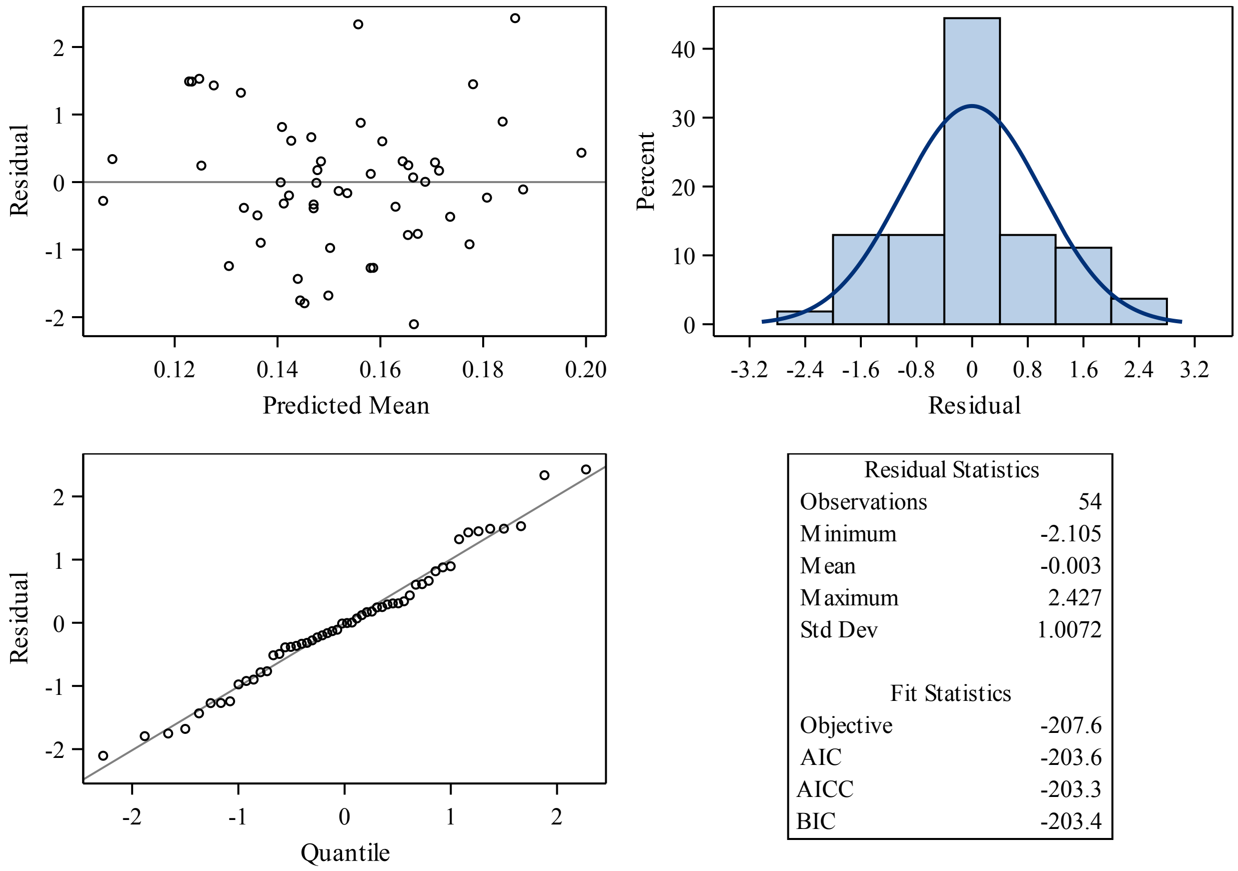

Residual Statistics	
Observations	54
Minimum	-2.105
Mean	-0.003
Maximum	2.427
Std Dev	1.0072

Fit Statistics	
Objective	-207.6
AIC	-203.6
AICC	-203.3
BIC	-203.4

1.4.6. Output from SAS (v. 9.3) Mixed Model analysis for CT at 34°C.

Model Information	
**Data Set**	WORK.THERMLONG
**Dependent Variable**	CT
**Covariance Structure**	Variance Components
**Estimation Method**	REML
**Residual Variance Method**	Profile
**Fixed Effects SE Method**	Model-Based
**Degrees of Freedom Method**	Satterthwaite

Class Level Information		
**Class**	**Levels**	**Values**
**T**	2	A C
**S**	2	0 1
**L**	8	A1 A2 A3 A4 C1 C2 C3 C4
**Gen**	2	Y Z
**Timing**	2	Afternoon Morning

Dimensions	
**Covariance Parameters**	3
**Columns in X**	15
**Columns in Z**	23
**Subjects**	1
**Max Obs per Subject**	47

Number of Observations	
**Number of Observations Read**	47
**Number of Observations Used**	47
**Number of Observations Not Used**	0

Covariance Parameter Estimates							
**Cov Parm**	**Estimate**	**Standard Error**	**Z Value**	**Pr > Z**	**Alpha**	**Lower**	**Upper**
**L(T)**	0	.	.	.	.	.	.
**S*L(T)**	0	.	.	.	.	.	.
**Residual**	0.001050	0.000238	4.42	<.0001	0.05	0.000704	0.001731

Fit Statistics	
-2 Res Log Likelihood	-126.8
AIC (Smaller is Better)	-124.8
AICC (Smaller is Better)	-124.7
BIC (Smaller is Better)	-124.7

Solution for Fixed Effects												
Effect	T	Gen	Timing	S	Estimate	Standard Error	DF	t Value	Pr > |t|	Alpha	Lower	Upper
Intercept					0.07370	0.04900	39	1.50	0.1406	0.05	-0.02542	0.1728
T	A				-0.01063	0.01399	39	-0.76	0.4520	0.05	-0.03893	0.01767
T	C				0	.	.	.	.	.	.	.
S				0	0.002934	0.01361	39	0.22	0.8304	0.05	-0.02459	0.03046
S				1	0	.	.	.	.	.	.	.
T*S	A			0	0.004822	0.01933	39	0.25	0.8044	0.05	-0.03429	0.04393
T*S	A			1	0	.	.	.	.	.	.	.
T*S	C			0	0	.	.	.	.	.	.	.
T*S	C			1	0	.	.	.	.	.	.	.
Gen		Y			0.02387	0.01498	39	1.59	0.1192	0.05	-0.00643	0.05418
Gen		Z			0	.	.	.	.	.	.	.
Timing			Afternoon		0.01946	0.01012	39	1.92	0.0618	0.05	-0.00101	0.03993
Timing			Morning		0	.	.	.	.	.	.	.
MB0					0.008657	0.001953	39	4.43	<.0001	0.05	0.004707	0.01261
Age					-0.00049	0.000258	39	-1.88	0.0669	0.05	-0.00101	0.000036

Type 3 Tests of Fixed Effects				
Effect	Num DF	Den DF	F Value	Pr > F
T	1	39	0.57	0.4542
S	1	39	0.23	0.6311
T*S	1	39	0.06	0.8044
Gen	1	39	2.54	0.1192
Timing	1	39	3.70	0.0618
MB0	1	39	19.65	<.0001
Age	1	39	3.55	0.0669

Least Squares Means												
**Effect**	**T**	**Gen**	**Timing**	**S**	**Estimate**	**Standard Error**	**DF**	**t Value**	**Pr > |t|**	**Alpha**	**Lower**	**Upper**
**T**	A				0.2370	0.007888	39	30.05	<.0001	0.05	0.2211	0.2530
**T**	C				0.2452	0.006787	39	36.13	<.0001	0.05	0.2315	0.2590
**S**				0	0.2438	0.007935	39	30.72	<.0001	0.05	0.2277	0.2598
**S**				1	0.2384	0.006869	39	34.71	<.0001	0.05	0.2246	0.2523
**T*S**	A			0	0.2409	0.01132	39	21.28	<.0001	0.05	0.2180	0.2638
**T*S**	A			1	0.2331	0.01091	39	21.37	<.0001	0.05	0.2111	0.2552
**T*S**	C			0	0.2467	0.01056	39	23.36	<.0001	0.05	0.2253	0.2681
**T*S**	C			1	0.2438	0.008556	39	28.49	<.0001	0.05	0.2265	0.2611
**Gen**		Y			0.2531	0.009710	39	26.06	<.0001	0.05	0.2334	0.2727
**Gen**		Z			0.2292	0.008194	39	27.97	<.0001	0.05	0.2126	0.2458
**Timing**			Afternoon		0.2508	0.007500	39	33.44	<.0001	0.05	0.2357	0.2660
**Timing**			Morning		0.2314	0.006643	39	34.83	<.0001	0.05	0.2180	0.2448

Studentized Residuals for CT

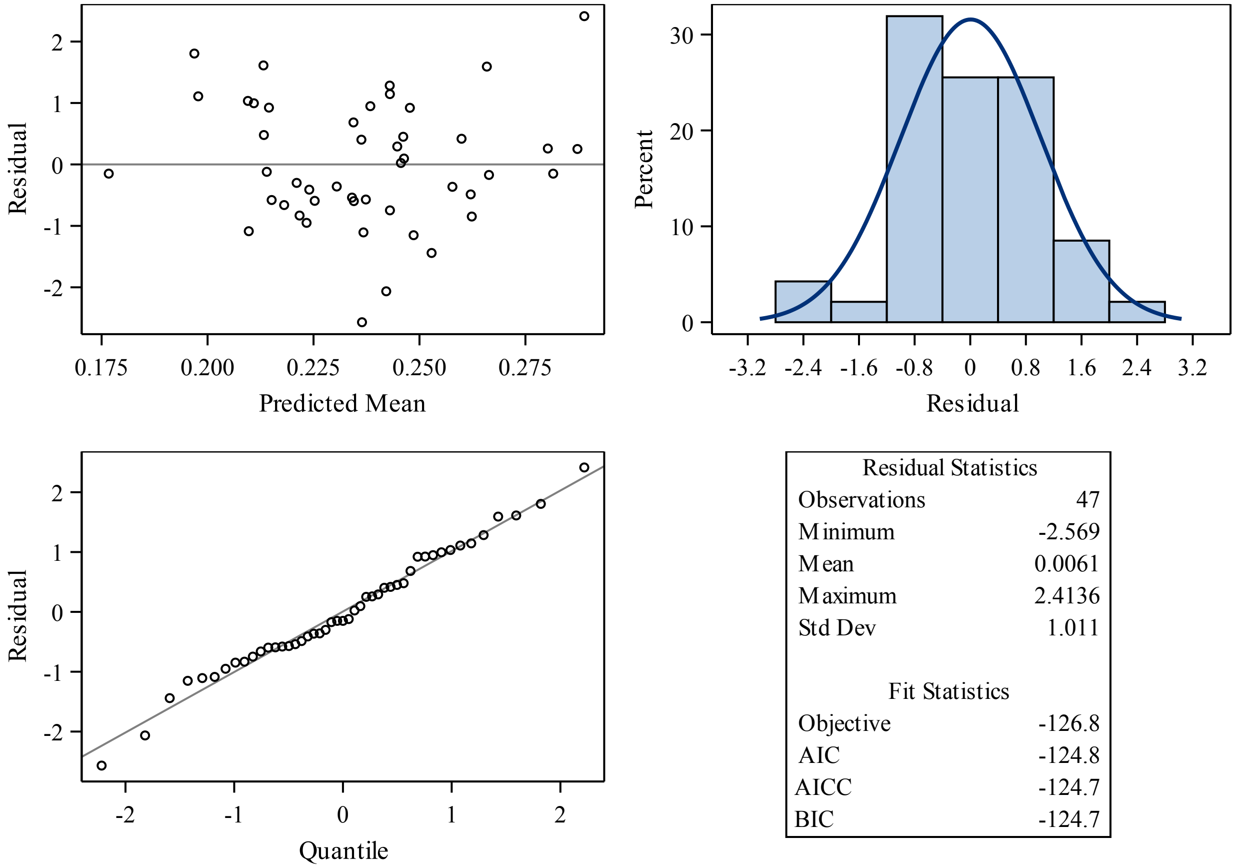

Residual Statistics	
Observations	47
Minimum	-2.569
Mean	0.0061
Maximum	2.4136
Std Dev	1.011
**Fit Statistics**	
Objective	-126.8
AIC	-124.8
AICC	-124.7
BIC	-124.7

Part 2. Analyses of traits associated with therogenic capacity

Mixed model analyses as described in Part 1.

2.1. $T_b$cold: T_b after maximum thermogenesis experiment. Output from SAS (v. 9.3) Mixed Model analysis for $T_b$cold after maximum thermogenesis experiment.

Model Information	
**Data Set**	WORK.THERMO
**Dependent Variable**	Tb_cold
**Covariance Structure**	Variance Components
**Estimation Method**	REML
**Residual Variance Method**	Profile
**Fixed Effects SE Method**	Model-Based
**Degrees of Freedom Method**	Satterthwaite

Class Level Information		
**Class**	**Levels**	**Values**
**T**	2	A C
**S**	2	0 1
**L**	8	A1 A2 A3 A4 C1 C2 C3 C4
**Gen**	2	Y Z

Dimensions	
**Covariance Parameters**	3
**Columns in X**	13
**Columns in Z**	24
**Subjects**	1
**Max Obs per Subject**	93

Number of Observations	
**Number of Observations Read**	101
**Number of Observations Used**	93
**Number of Observations Not Used**	8

Covariance Parameter Estimates							
**Cov Parm**	**Estimate**	**Standard Error**	**Z Value**	**Pr > Z**	**Alpha**	**Lower**	**Upper**
**L(T)**	0	.	.	.	.	.	.
**S*L(T)**	0	.	.	.	.	.	.
**Residual**	1.7268	0.2633	6.56	<.0001	0.05	1.3079	2.3860

Fit Statistics	
-2 Res Log Likelihood	324.2
AIC (Smaller is Better)	326.2
AICC (Smaller is Better)	326.2
BIC (Smaller is Better)	326.2

Solution for Fixed Effects											
Effect	T	Gen	S	Estimate	Standard Error	DF	t Value	Pr > |t|	Alpha	Lower	Upper
Intercept				24.9754	1.2291	86	20.32	<.0001	0.05	22.5321	27.4187
T	A			0.3806	0.4095	86	0.93	0.3553	0.05	-0.4334	1.1945
T	C			0	.	.	.	.	.	.	.
S			0	0.7633	0.3978	86	1.92	0.0584	0.05	-0.02759	1.5541
S			1	0	.	.	.	.	.	.	.
T*S	A		0	-0.5207	0.5474	86	-0.95	0.3442	0.05	-1.6089	0.5675
T*S	A		1	0	.	.	.	.	.	.	.
T*S	C		0	0	.	.	.	.	.	.	.
T*S	C		1	0	.	.	.	.	.	.	.
Gen		Y		-0.8669	0.3999	86	-2.17	0.0329	0.05	-1.6619	-0.07188
Gen		Z		0	.	.	.	.	.	.	.
MB_Tbcold				0.1235	0.04823	86	2.56	0.0122	0.05	0.02765	0.2194
Age_Tbcold				0.003362	0.006211	86	0.54	0.5898	0.05	-0.00899	0.01571

Type 3 Tests of Fixed Effects				
Effect	Num DF	Den DF	F Value	Pr > F
T	1	86	0.15	0.7002
S	1	86	2.74	0.1018
T*S	1	86	0.90	0.3442
Gen	1	86	4.70	0.0329
MB_Tbcold	1	86	6.56	0.0122
Age_Tbcold	1	86	0.29	0.5898

Least Squares Means											
**Effect**	**T**	**Gen**	**S**	**Estimate**	**Standard Error**	**DF**	**t Value**	**Pr > |t|**	**Alpha**	**Lower**	**Upper**
**T**	A			28.6027	0.2187	86	130.76	<.0001	0.05	28.1679	29.0376
**T**	C			28.4825	0.2018	86	141.15	<.0001	0.05	28.0814	28.8836
**S**			0	28.7941	0.2112	86	136.34	<.0001	0.05	28.3742	29.2139
**S**			1	28.2912	0.2044	86	138.38	<.0001	0.05	27.8847	28.6976
**T*S**	A		0	28.7240	0.2932	86	97.97	<.0001	0.05	28.1412	29.3068
**T*S**	A		1	28.4814	0.3130	86	90.99	<.0001	0.05	27.8591	29.1037
**T*S**	C		0	28.8641	0.3019	86	95.61	<.0001	0.05	28.2640	29.4643
**T*S**	C		1	28.1009	0.2635	86	106.64	<.0001	0.05	27.5770	28.6247
**Gen**		Y		28.1092	0.2647	86	106.19	<.0001	0.05	27.5829	28.6354
**Gen**		Z		28.9761	0.2237	86	129.51	<.0001	0.05	28.5313	29.4208

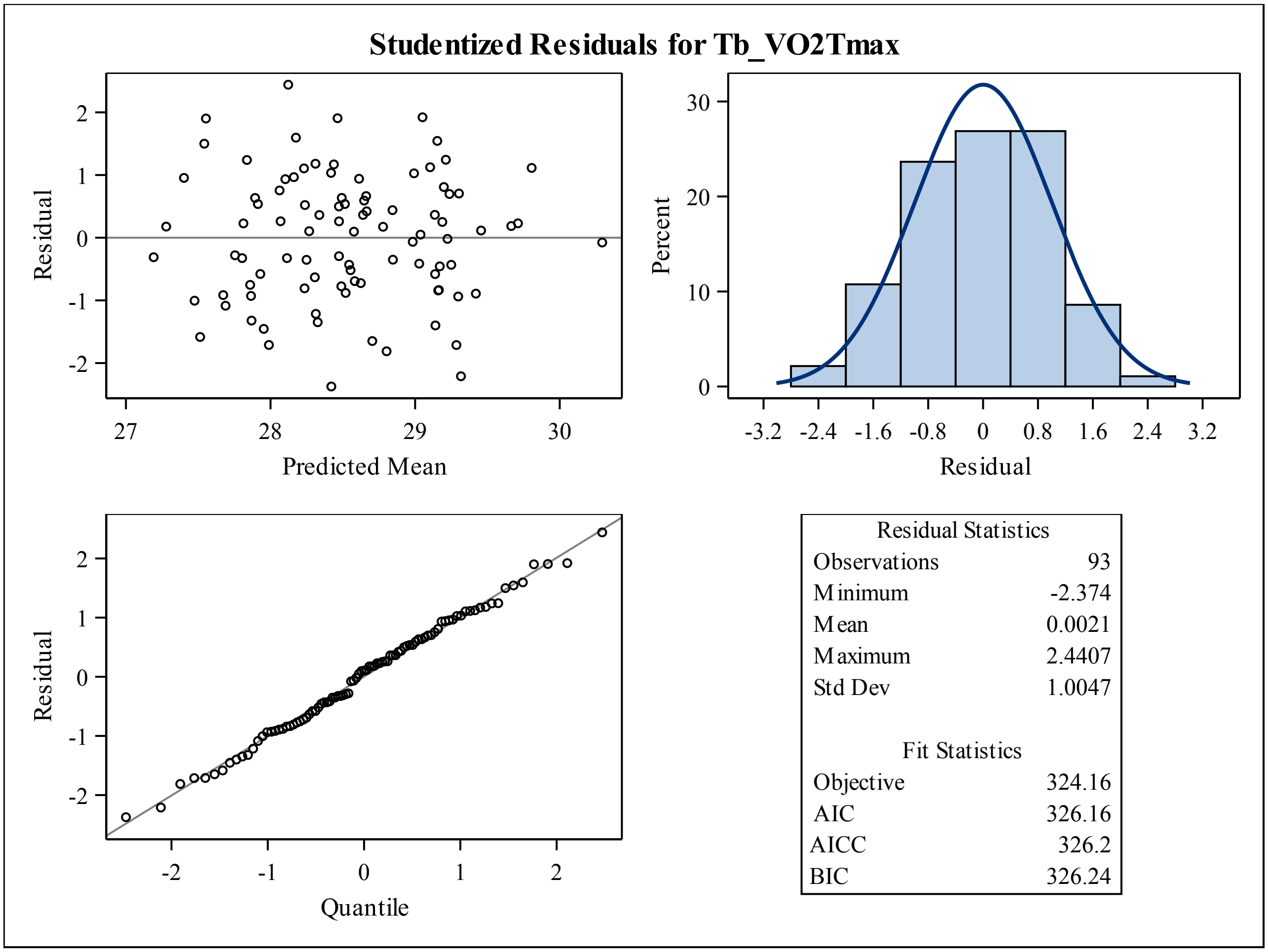

2.2. $\dot{V}O_2$cold: maximal MR during thermogenesis experiment. Output from SAS (v. 9.3) Mixed Model analysis for $\dot{V}O_2$cold from the maximum thermogenesis experiment.

Model Information	
Data Set	WORK.THERMO
Dependent Variable	VO2cold
Covariance Structure	Variance Components
Estimation Method	REML
Residual Variance Method	Profile
Fixed Effects SE Method	Model-Based
Degrees of Freedom Method	Satterthwaite

Class Level Information		
Class	Levels	Values
T	2	A C
S	2	0 1
L	8	A1 A2 A3 A4 C1 C2 C3 C4
Gen	2	Y Z

Dimensions	
Covariance Parameters	3
Columns in X	13
Columns in Z	24
Subjects	1
Max Obs per Subject	100

Number of Observations	
Number of Observations Read	100
Number of Observations Used	100
Number of Observations Not Used	0

Covariance Parameter Estimates							
Cov Parm	Estimate	Standard Error	Z Value	Pr > Z	Alpha	Lower	Upper
L(T)	0	.	.	.	.	.	.
S*L(T)	0	.	.	.	.	.	.
Residual	0.1959	0.02872	6.82	<.0001	0.05	0.1498	0.2670

Fit Statistics	
**-2 Res Log Likelihood**	145.9
**AIC (Smaller is Better)**	147.9
**AICC (Smaller is Better)**	147.9
**BIC (Smaller is Better)**	147.9

Solution for Fixed Effects											
**Effect**	**T**	**Gen**	**S**	**Estimate**	**Standard Error**	**DF**	**t Value**	**Pr > |t|**	**Alpha**	**Lower**	**Upper**
**Intercept**				-0.1124	0.4043	93	-0.28	0.7816	0.05	-0.9153	0.6904
**T**	A			0.4967	0.1338	93	3.71	0.0003	0.05	0.2310	0.7623
**T**	C			0	.	.	.	.	.	.	.
**S**			0	0.2522	0.1301	93	1.94	0.0556	0.05	-0.00616	0.5105
**S**			1	0	.	.	.	.	.	.	.
**T*S**	A		0	-0.02196	0.1777	93	-0.12	0.9020	0.05	-0.3749	0.3310
**T*S**	A		1	0	.	.	.	.	.	.	.
**T*S**	C		0	0	.	.	.	.	.	.	.
**T*S**	C		1	0	.	.	.	.	.	.	.
**Gen**		Y		-0.2474	0.1317	93	-1.88	0.0634	0.05	-0.5090	0.01413
**Gen**		Z		0	.	.	.	.	.	.	.
**MB_VO2cold**				0.1592	0.01570	93	10.14	<.0001	0.05	0.1280	0.1903
**Age_VO2cold**				0.002389	0.002068	93	1.16	0.2510	0.05	-0.00172	0.006495

Type 3 Tests of Fixed Effects				
**Effect**	**Num DF**	**Den DF**	**F Value**	**Pr > F**
**T**	1	93	22.60	<.0001
**S**	1	93	5.68	0.0192
**T*S**	1	93	0.02	0.9020
**Gen**	1	93	3.53	0.0634
**MB_VO2cold**	1	93	102.75	<.0001
**Age_VO2cold**	1	93	1.33	0.2510

Least Squares Means											
**Effect**	**T**	**Gen**	**S**	**Estimate**	**Standard Error**	**DF**	**t Value**	**Pr > |t|**	**Alpha**	**Lower**	**Upper**
**T**	A			4.6890	0.07162	93	65.47	<.0001	0.05	4.5468	4.8313
**T**	C			4.2033	0.06598	93	63.71	<.0001	0.05	4.0723	4.3344
**S**			0	4.5668	0.07043	93	64.84	<.0001	0.05	4.4269	4.7067
**S**			1	4.3256	0.06653	93	65.02	<.0001	0.05	4.1935	4.4577
**T*S**	A		0	4.8042	0.09699	93	49.53	<.0001	0.05	4.6115	4.9968
**T*S**	A		1	4.5739	0.1026	93	44.57	<.0001	0.05	4.3701	4.7777
**T*S**	C		0	4.3294	0.09950	93	43.51	<.0001	0.05	4.1318	4.5270
**T*S**	C		1	4.0773	0.08525	93	47.83	<.0001	0.05	3.9080	4.2465
**Gen**		Y		4.3225	0.08865	93	48.76	<.0001	0.05	4.1464	4.4985
**Gen**		Z		4.5699	0.07127	93	64.12	<.0001	0.05	4.4284	4.7114

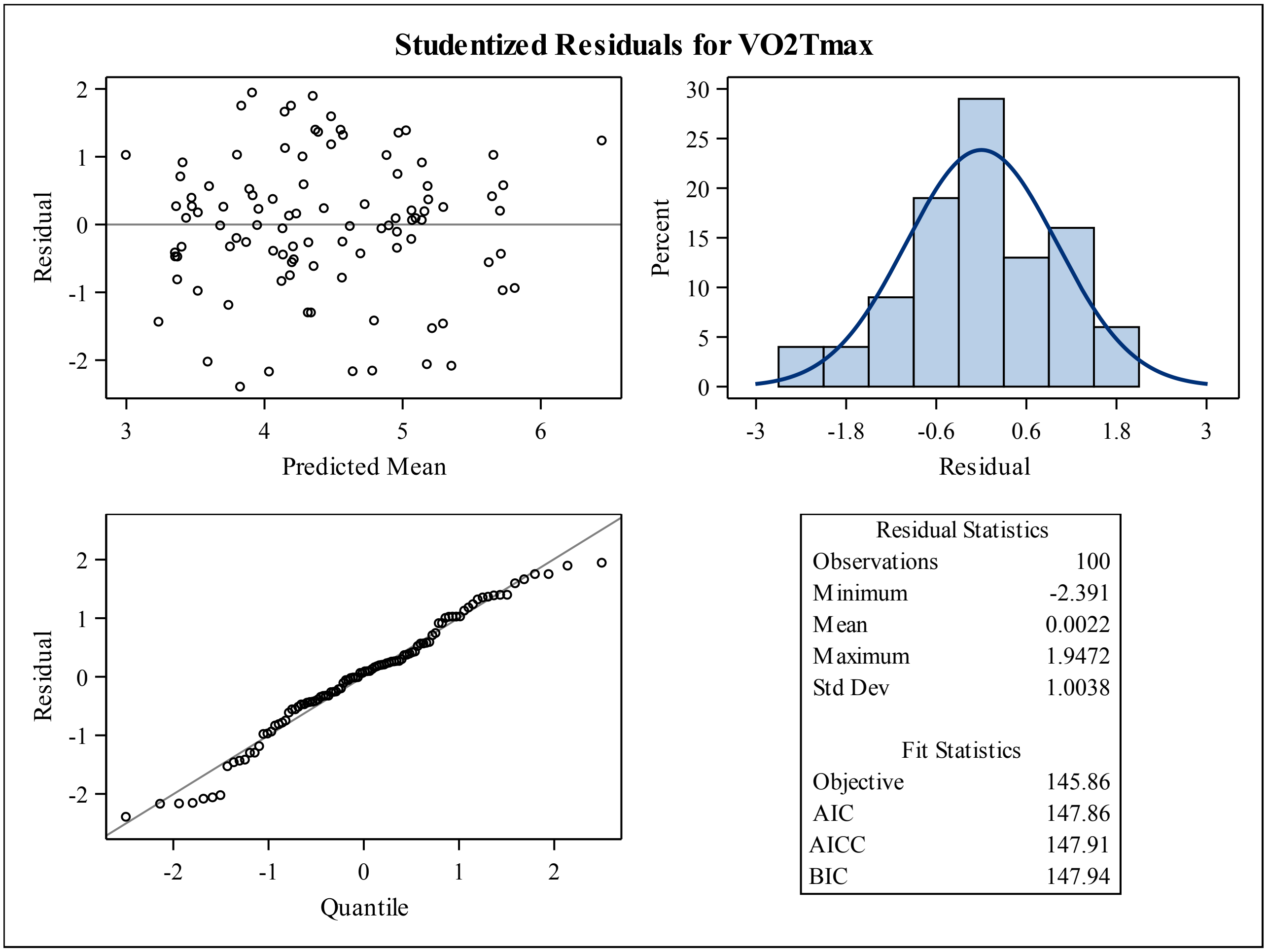

Part 3. Repeated measures analyses for $T_b$rmr, RMR and CT at temperatures around the thermoneutral zone

We used a repeated-measures extension of the Mixed Model described in Part 1 to perform analyses for combined results from the trial performed at temperatures around the TNZ (25, 28, 31, and 34°C). In addition to the factors described above, the model included a fixed repeated-measures factor for T_a (treated as a grouping factor), interactions of T_a with Selection, Sex and Line, and the random effect of Individual ("subject"). As the analyses performed separately for each T_a revealed large differences of residual variance, in the repeated measures model an "unstructured" type of residual (co)variance matrix was assumed. To compare the four T_a groups, Tukey-Kramer post-hoc tests were performed. In all the above analyses, variance was constrained to non-negative values (default approach in SAS), and Satterthwaite approximation for non-orthogonal models was applied to calculate the denominator degrees of freedom.

3.1. $T_b$rmr. Output from SAS (v. 9.3) Repeated Measures Model for $T_b$rmr for combined results from the trial performed at temperatures around the TNZ (25, 28, 31, and 34°C).

Model Information	
**Data Set**	WORK.THERMLONG
**Dependent Variable**	Tb_rmr
**Covariance Structures**	Variance Components, Unstructured
**Subject Effect**	ID
**Estimation Method**	REML
**Residual Variance Method**	None
**Fixed Effects SE Method**	Model-Based
**Degrees of Freedom Method**	Satterthwaite

Class Level Information		
**Class**	**Levels**	**Values**
**Ta**	4	25 28 31 34
**T**	2	A C
**S**	2	0 1
**L**	8	A1 A2 A3 A4 C1 C2 C3 C4
**Gen**	2	Y Z
**Timing**	2	Afternoon Morning
**ID**	63	Y49489 Y49560 Y49937 Y50477 Y50676 Y50901 Y50954 Y50961 Y51184 Y51240 Y51319 Y51482 Y51577 Y51662 Y51745 Y51767 Y51798 Y51821 Y51870 Y52096 Y52119 Y52321 Y52640 Y52843 Y52852 Y53007 Y53011 Y53143 Y53414 Y53504 Y53514 Y53605 Y53641 Z54929 Z55058 Z55209 Z55222 Z55274 Z55665 Z55667 Z55702 Z55739 Z55741 Z55839 Z55851 Z55968 Z56223 Z56442 Z56494 Z56531 Z56557 Z56568 Z56661 Z56675 Z56787 Z56885 Z56996 Z60028 Z60236 Z60558 Z60607 Z60720 Z60756

Dimensions	
**Covariance Parameters**	13
**Columns in X**	35
**Columns in Z**	56
**Subjects**	1
**Max Obs per Subject**	243

Number of Observations	
Number of Observations Read	243
Number of Observations Used	243
Number of Observations Not Used	0

Cov Parm	Subject	Estimate	Standard Error	Z Value	Pr Z	Alpha	Lower	Upper
L(T)		0.01614	0.03372	0.48	0.3161	0.05	0.002262	54864
S*L(T)		0	.	.	.	.	.	.
Ta*L(T)		0	.	.	.	.	.	.
UN(1,1)	ID	0.3700	0.07168	5.16	<.0001	0.05	0.2617	0.5632
UN(2,1)	ID	0.2220	0.05979	3.71	0.0002	0.05	0.1048	0.3392
UN(2,2)	ID	0.3432	0.06940	4.95	<.0001	0.05	0.2394	0.5332
UN(3,1)	ID	0.2398	0.06525	3.68	0.0002	0.05	0.1119	0.3677
UN(3,2)	ID	0.2286	0.06302	3.63	0.0003	0.05	0.1050	0.3521
UN(3,3)	ID	0.4263	0.08425	5.06	<.0001	0.05	0.2996	0.6550
UN(4,1)	ID	-0.00133	0.1264	-0.01	0.9916	0.05	-0.2491	0.2464
UN(4,2)	ID	-0.00527	0.1243	-0.04	0.9662	0.05	-0.2489	0.2383
UN(4,3)	ID	0.3417	0.1400	2.44	0.0146	0.05	0.06737	0.6160
UN(4,4)	ID	1.7875	0.3622	4.93	<.0001	0.05	1.2461	2.7799

Fit Statistics	
-2 Res Log Likelihood	492.5
AIC (Smaller is Better)	514.5
AICC (Smaller is Better)	515.7
BIC (Smaller is Better)	515.4

Effect	T	Gen	Timing	Ta	S	Estimate	Standard Error	DF	t Value	Pr > |t|	Alpha	Lower	Upper
Intercept						39.0819	0.7011	67.9	55.74	<.0001	0.05	37.6828	40.4810
T	A					1.5715	0.3847	62.2	4.08	0.0001	0.05	0.8025	2.3405
T	C					0	.	.	.	.	.	.	.
S					0	-0.2124	0.3798	67.6	-0.56	0.5779	0.05	-0.9704	0.5456

Solution for Fixed Effects													
**Effect**	**T**	**Gen**	**Timing**	**Ta**	**S**	**Estimate**	**Standard Error**	**DF**	**t Value**	**Pr > |t|**	**Alpha**	**Lower**	**Upper**
**S**					1	0	.	.	.	.	.	.	.
**Ta**				25		-0.9642	0.3193	55	-3.02	0.0038	0.05	-1.6041	-0.3242
**Ta**				28		-1.0183	0.3182	52.9	-3.20	0.0023	0.05	-1.6565	-0.3801
**Ta**				31		-1.0145	0.2709	54.2	-3.74	0.0004	0.05	-1.5575	-0.4714
**Ta**				34		0	.	.	.	.	.	.	.
**T*S**	A				0	0.009785	0.2573	50	0.04	0.9698	0.05	-0.5070	0.5265
**T*S**	A				1	0	.	.	.	.	.	.	.
**T*S**	C				0	0	.	.	.	.	.	.	.
**T*S**	C				1	0	.	.	.	.	.	.	.
**Ta*T**	A			25		-1.2935	0.3809	55.2	-3.40	0.0013	0.05	-2.0568	-0.5302
**Ta*T**	C			25		0	.	.	.	.	.	.	.
**Ta*T**	A			28		-1.3373	0.3800	53.1	-3.52	0.0009	0.05	-2.0994	-0.5751
**Ta*T**	C			28		0	.	.	.	.	.	.	.
**Ta*T**	A			31		-1.2527	0.3233	54.3	-3.87	0.0003	0.05	-1.9007	-0.6046
**Ta*T**	C			31		0	.	.	.	.	.	.	.
**Ta*T**	A			34		0	.	.	.	.	.	.	.
**Ta*T**	C			34		0	.	.	.	.	.	.	.
**Ta*S**				25	0	0.2655	0.3812	55.3	0.70	0.4890	0.05	-0.4983	1.0293
**Ta*S**				25	1	0	.	.	.	.	.	.	.
**Ta*S**				28	0	0.3776	0.3803	53.4	0.99	0.3253	0.05	-0.3852	1.1403
**Ta*S**				28	1	0	.	.	.	.	.	.	.
**Ta*S**				31	0	0.5335	0.3238	54.4	1.65	0.1052	0.05	-0.1155	1.1825
**Ta*S**				31	1	0	.	.	.	.	.	.	.
**Ta*S**				34	0	0	.	.	.	.	.	.	.
**Ta*S**				34	1	0	.	.	.	.	.	.	.
**Gen**		Y				0.2673	0.1799	51.2	1.49	0.1435	0.05	-0.09381	0.6283
**Gen**		Z				0	.	.	.	.	.	.	.
**Timing**			Afternoon			-0.1425	0.1305	49.4	-1.09	0.2803	0.05	-0.4048	0.1198
**Timing**			Morning			0	.	.	.	.	.	.	.
**MB0**						0.003841	0.02698	48.6	0.14	0.8874	0.05	-0.05040	0.05808
**Age**						-0.00297	0.003042	52.2	-0.98	0.3330	0.05	-0.00908	0.003131

Type 3 Tests of Fixed Effects				
**Effect**	**Num DF**	**Den DF**	**F Value**	**Pr > F**
**T**	1	8.69	10.78	0.0099
**S**	1	56.8	0.29	0.5928
**Ta**	3	55.9	24.03	<.0001
**T*S**	1	50	0.00	0.9698
**Ta*T**	3	55.8	5.12	0.0034
**Ta*S**	3	55.9	2.67	0.0563
**Gen**	1	51.2	2.21	0.1435
**Timing**	1	49.4	1.19	0.2803
**MB0**	1	48.6	0.02	0.8874
**Age**	1	52.2	0.95	0.3330

Least Squares Means															
**Effect**	**T**	**Gen**	**Timing**	**Ta**	**S**	**MB0**	**Age**	**Estimate**	**Standard Error**	**DF**	**t Value**	**Pr > |t|**	**Alpha**	**Lower**	**Upper**
**T**	A					24.86	141.38	38.7167	0.1269	8.21	305.07	<.0001	0.05	38.4253	39.0080
**T**	C					24.86	141.38	38.1111	0.1234	7.06	308.93	<.0001	0.05	37.8199	38.4023
**S**					0	24.86	141.38	38.4572	0.1184	20.1	324.82	<.0001	0.05	38.2103	38.7041
**S**					1	24.86	141.38	38.3706	0.1152	20	332.97	<.0001	0.05	38.1302	38.6110
**Ta**				25		24.86	141.38	38.0233	0.08926	6.88	425.98	<.0001	0.05	37.8115	38.2351
**Ta**				28		24.86	141.38	38.0033	0.08764	6.08	433.61	<.0001	0.05	37.7895	38.2171
**Ta**				31		24.86	141.38	38.1274	0.09450	8.88	403.46	<.0001	0.05	37.9132	38.3416
**Ta**				34		24.86	141.38	39.5015	0.1802	49.4	219.18	<.0001	0.05	39.1394	39.8636
**T*S**	A				0	24.86	141.38	38.7624	0.1603	19.3	241.81	<.0001	0.05	38.4273	39.0976
**T*S**	A				1	24.86	141.38	38.6709	0.1665	21.3	232.23	<.0001	0.05	38.3249	39.0169
**T*S**	C				0	24.86	141.38	38.1520	0.1705	19.2	223.74	<.0001	0.05	37.7953	38.5086
**T*S**	C				1	24.86	141.38	38.0702	0.1505	15.8	252.93	<.0001	0.05	37.7508	38.3896
**Ta*T**	A			25		24.86	141.38	38.1648	0.1330	8.3	287.06	<.0001	0.05	37.8601	38.4695
**Ta*T**	C			25		24.86	141.38	37.8819	0.1291	7.29	293.39	<.0001	0.05	37.5790	38.1847
**Ta*T**	A			28		24.86	141.38	38.1229	0.1309	7.41	291.32	<.0001	0.05	37.8169	38.4289
**Ta*T**	C			28		24.86	141.38	37.8837	0.1277	6.67	296.77	<.0001	0.05	37.5788	38.1887
**Ta*T**	A			31		24.86	141.38	38.2893	0.1413	10.8	271.06	<.0001	0.05	37.9779	38.6007
**Ta*T**	C			31		24.86	141.38	37.9655	0.1356	9.1	280.08	<.0001	0.05	37.6594	38.2717
**Ta*T**	A			34		24.86	141.38	40.2897	0.2623	51.5	153.59	<.0001	0.05	39.7632	40.8162

Least Squares Means															
**Effect**	**T**	**Gen**	**Timing**	**Ta**	**S**	**MB0**	**Age**	**Estimate**	**Standard Error**	**DF**	**t Value**	**Pr > |t|**	**Alpha**	**Lower**	**Upper**
**Ta*T**	C			34		24.86	141.38	38.7133	0.2527	50.2	153.21	<.0001	0.05	38.2058	39.2208
**Ta*S**				25	0	24.86	141.38	38.0523	0.1237	18.1	307.56	<.0001	0.05	37.7925	38.3121
**Ta*S**				25	1	24.86	141.38	37.9943	0.1229	19.2	309.18	<.0001	0.05	37.7373	38.2513
**Ta*S**				28	0	24.86	141.38	38.0884	0.1229	16.4	309.96	<.0001	0.05	37.8284	38.3483
**Ta*S**				28	1	24.86	141.38	37.9183	0.1194	16	317.53	<.0001	0.05	37.6651	38.1715
**Ta*S**				31	0	24.86	141.38	38.2904	0.1320	23.5	290.05	<.0001	0.05	38.0176	38.5632
**Ta*S**				31	1	24.86	141.38	37.9644	0.1298	24	292.38	<.0001	0.05	37.6965	38.2324
**Ta*S**				34	0	24.86	141.38	39.3977	0.2611	58.8	150.92	<.0001	0.05	38.8753	39.9202
**Ta*S**				34	1	24.86	141.38	39.6052	0.2460	56.9	160.99	<.0001	0.05	39.1126	40.0979
**Gen**		Y				24.86	141.38	38.5475	0.1220	23.5	315.99	<.0001	0.05	38.2955	38.7996
**Gen**		Z				24.86	141.38	38.2803	0.1250	26.8	306.36	<.0001	0.05	38.0238	38.5367
**Timing**			Afternoon			24.86	141.38	38.3426	0.1044	14.6	367.21	<.0001	0.05	38.1195	38.5658
**Timing**			Morning			24.86	141.38	38.4851	0.1092	17.1	352.28	<.0001	0.05	38.2547	38.7155
**T**	A					25.00	140.00	38.7213	0.1261	8.06	306.99	<.0001	0.05	38.4308	39.0118
**T**	C					25.00	140.00	38.1158	0.1244	7.21	306.36	<.0001	0.05	37.8233	38.4083
**S**					0	25.00	140.00	38.4619	0.1204	20.7	319.43	<.0001	0.05	38.2112	38.7125
**S**					1	25.00	140.00	38.3752	0.1134	19.3	338.37	<.0001	0.05	38.1381	38.6124
**Ta**				25		25.00	140.00	38.0280	0.08933	6.86	425.72	<.0001	0.05	37.8159	38.2401
**Ta**				28		25.00	140.00	38.0080	0.08777	6.08	433.02	<.0001	0.05	37.7939	38.2221
**Ta**				31		25.00	140.00	38.1321	0.09461	8.87	403.06	<.0001	0.05	37.9176	38.3466
**Ta**				34		25.00	140.00	39.5061	0.1804	49.4	218.96	<.0001	0.05	39.1436	39.8686
**T*S**	A				0	25.00	140.00	38.7671	0.1610	19.5	240.84	<.0001	0.05	38.4308	39.1034
**T*S**	A				1	25.00	140.00	38.6756	0.1647	20.8	234.84	<.0001	0.05	38.3329	39.0182
**T*S**	C				0	25.00	140.00	38.1566	0.1727	19.6	220.94	<.0001	0.05	37.7959	38.5174
**T*S**	C				1	25.00	140.00	38.0749	0.1498	15.6	254.25	<.0001	0.05	37.7567	38.3931
**Ta*T**	A			25		25.00	140.00	38.1694	0.1321	8.13	288.91	<.0001	0.05	37.8656	38.4733
**Ta*T**	C			25		25.00	140.00	37.8865	0.1301	7.43	291.29	<.0001	0.05	37.5825	38.1905
**Ta*T**	A			28		25.00	140.00	38.1276	0.1301	7.27	293.10	<.0001	0.05	37.8223	38.4329
**Ta*T**	C			28		25.00	140.00	37.8884	0.1286	6.8	294.56	<.0001	0.05	37.5824	38.1944
**Ta*T**	A			31		25.00	140.00	38.2939	0.1405	10.7	272.50	<.0001	0.05	37.9835	38.6044
**Ta*T**	C			31		25.00	140.00	37.9702	0.1365	9.24	278.26	<.0001	0.05	37.6628	38.2776
**Ta*T**	A			34		25.00	140.00	40.2944	0.2620	51.4	153.79	<.0001	0.05	39.7684	40.8203
**Ta*T**	C			34		25.00	140.00	38.7179	0.2533	50.4	152.87	<.0001	0.05	38.2093	39.2265
**Ta*S**				25	0	25.00	140.00	38.0570	0.1255	18.6	303.18	<.0001	0.05	37.7939	38.3201
**Ta*S**				25	1	25.00	140.00	37.9990	0.1211	18.5	313.68	<.0001	0.05	37.7450	38.2530

Least Squares Means															
**Effect**	**T**	**Gen**	**Timing**	**Ta**	**S**	**MB0**	**Age**	**Estimate**	**Standard Error**	**DF**	**t Value**	**Pr > |t|**	**Alpha**	**Lower**	**Upper**
**Ta*S**				28	0	25.00	140.00	38.0930	0.1248	17	305.26	<.0001	0.05	37.8297	38.3563
**Ta*S**				28	1	25.00	140.00	37.9229	0.1176	15.3	322.41	<.0001	0.05	37.6727	38.1732
**Ta*S**				31	0	25.00	140.00	38.2951	0.1338	24.1	286.29	<.0001	0.05	38.0190	38.5711
**Ta*S**				31	1	25.00	140.00	37.9691	0.1282	23.3	296.17	<.0001	0.05	37.7041	38.2341
**Ta*S**				34	0	25.00	140.00	39.4024	0.2621	59.5	150.34	<.0001	0.05	38.8780	39.9267
**Ta*S**				34	1	25.00	140.00	39.6099	0.2452	56.3	161.53	<.0001	0.05	39.1187	40.1011
**Gen**		Y				25.00	140.00	38.5522	0.1250	24.9	308.53	<.0001	0.05	38.2947	38.8096
**Gen**		Z				25.00	140.00	38.2849	0.1222	25.3	313.18	<.0001	0.05	38.0333	38.5365
**Timing**			Afternoon			25.00	140.00	38.3473	0.1054	14.9	363.98	<.0001	0.05	38.1226	38.5720
**Timing**			Morning			25.00	140.00	38.4898	0.1086	16.7	354.32	<.0001	0.05	38.2603	38.7193

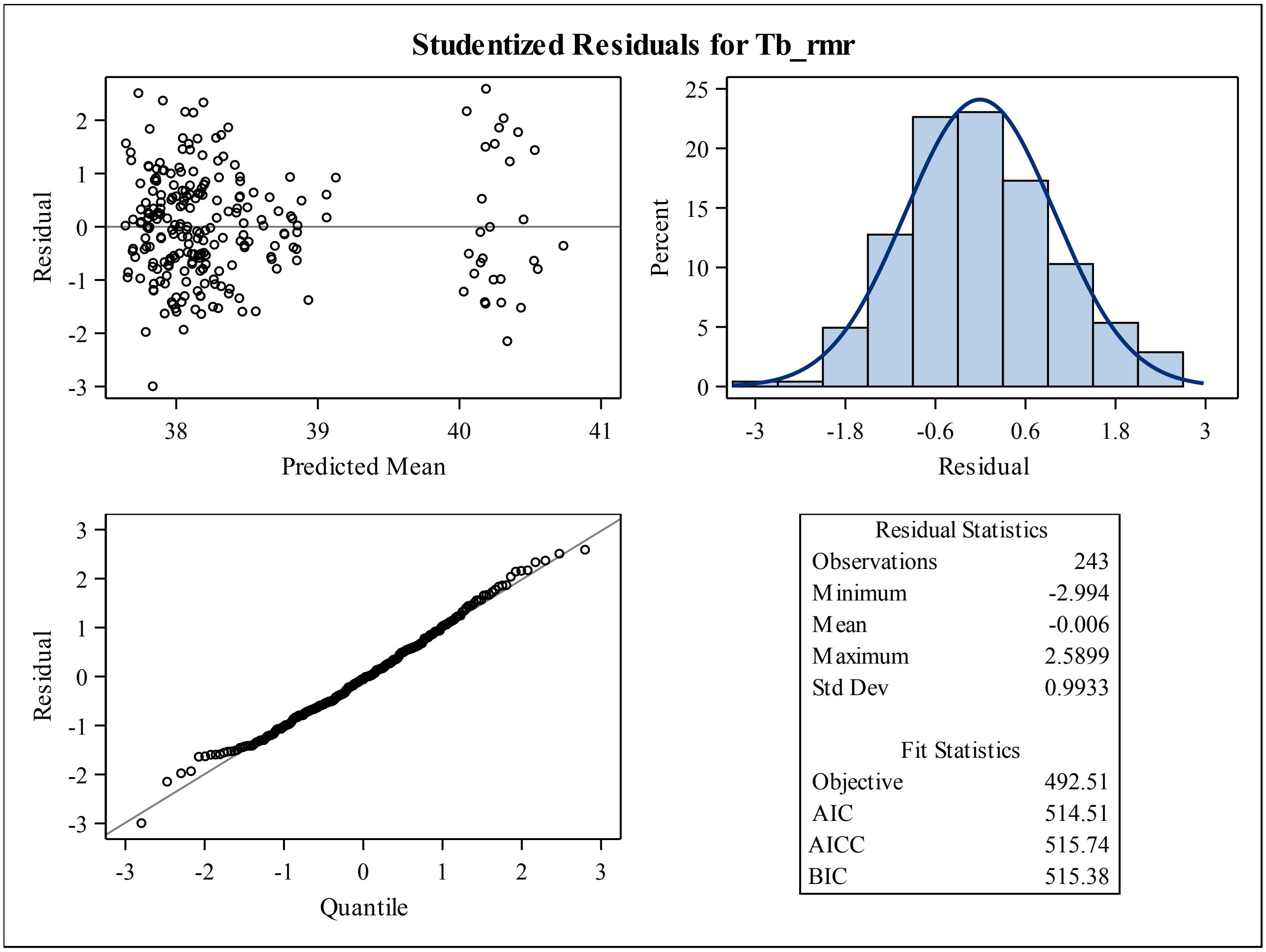

3.2. RMR. Output from SAS (v. 9.3) Repeated Measures Model for RMR for combined results from the trial performed at temperatures around the TNZ (25, 28, 31, and 34°C).

Model Information	
**Data Set**	WORK.THERMLONG
**Dependent Variable**	RMR
**Covariance Structures**	Variance Components, Unstructured
**Subject Effect**	ID
**Estimation Method**	REML
**Residual Variance Method**	None
**Fixed Effects SE Method**	Model-Based
**Degrees of Freedom Method**	Satterthwaite

Class Level Information		
**Class**	**Levels**	**Values**
**Ta**	4	25 28 31 34
**T**	2	A C
**S**	2	0 1
**L**	8	A1 A2 A3 A4 C1 C2 C3 C4
**Gen**	2	Y Z
**Timing**	2	Afternoon Morning
**ID**	64	Y49489 Y49560 Y49937 Y50477 Y50676 Y50901 Y50954 Y50961 Y51184 Y51188 Y51319 Y51482 Y51577 Y51662 Y51745 Y51767 Y51798 Y51821 Y51870 Y52096 Y52119 Y52321 Y52640 Y52843 Y52852 Y53007 Y53011 Y53143 Y53414 Y53504 Y53514 Y53605 Z54676 Z54929 Z55058 Z55209 Z55222 Z55274 Z55665 Z55667 Z55702 Z55739 Z55741 Z55839 Z55851 Z55968 Z56052 Z56223 Z56442 Z56494 Z56531 Z56557 Z56568 Z56661 Z56675 Z56787 Z56885 Z56996 Z60028 Z60236 Z60558 Z60607 Z60720 Z60756

Dimensions	
**Covariance Parameters**	13
**Columns in X**	35
**Columns in Z**	56
**Subjects**	1
**Max Obs per Subject**	229

Number of Observations	
**Number of Observations Read**	229
**Number of Observations Used**	229
**Number of Observations Not Used**	0

Covariance Parameter Estimates								
**Cov Parm**	**Subject**	**Estimate**	**Standard Error**	**Z Value**	**Pr Z**	**Alpha**	**Lower**	**Upper**
**L(T)**		5.42E-20	.	.	.	.	.	.
**S*L(T)**		0.001479	0.002290	0.65	0.2592	0.05	0.000271	5.7218
**Ta*L(T)**		0.000508	0.000733	0.69	0.2440	0.05	0.000099	0.6748
**UN(1,1)**	ID	0.01378	0.002842	4.85	<.0001	0.05	0.009555	0.02162
**UN(2,1)**	ID	0.01073	0.002763	3.88	0.0001	0.05	0.005316	0.01615
**UN(2,2)**	ID	0.01725	0.003661	4.71	<.0001	0.05	0.01184	0.02746
**UN(3,1)**	ID	0.009310	0.003659	2.54	0.0109	0.05	0.002139	0.01648
**UN(3,2)**	ID	0.01447	0.004309	3.36	0.0008	0.05	0.006025	0.02292
**UN(3,3)**	ID	0.03403	0.007182	4.74	<.0001	0.05	0.02340	0.05401
**UN(4,1)**	ID	0.003348	0.004588	0.73	0.4656	0.05	-0.00564	0.01234
**UN(4,2)**	ID	0.01022	0.005247	1.95	0.0513	0.05	-0.00006	0.02051
**UN(4,3)**	ID	0.02457	0.008654	2.84	0.0045	0.05	0.007613	0.04154
**UN(4,4)**	ID	0.04489	0.01260	3.56	0.0002	0.05	0.02770	0.08505

Fit Statistics	
**-2 Res Log Likelihood**	-201.3
**AIC (Smaller is Better)**	-177.3
**AICC (Smaller is Better)**	-175.7
**BIC (Smaller is Better)**	-176.3

Solution for Fixed Effects													
**Effect**	**T**	**Gen**	**Timing**	**Ta**	**S**	**Estimate**	**Standard Error**	**DF**	**t Value**	**Pr > |t|**	**Alpha**	**Lower**	**Upper**
**Intercept**						0.4444	0.1539	58.7	2.89	0.0054	0.05	0.1365	0.7523
**T**	A					0.09522	0.07279	38.9	1.31	0.1985	0.05	-0.05203	0.2425
**T**	C					0	.	.	.	.	.	.	.
**S**					0	-0.09439	0.07136	33.6	-1.32	0.1949	0.05	-0.2395	0.05070

Solution for Fixed Effects													
**Effect**	**T**	**Gen**	**Timing**	**Ta**	**S**	**Estimate**	**Standard Error**	**DF**	**t Value**	**Pr > |t|**	**Alpha**	**Lower**	**Upper**
**S**					1	0	.	.	.	.	.	.	.
**Ta**				25		-0.05735	0.05220	44.2	-1.10	0.2779	0.05	-0.1625	0.04784
**Ta**				28		-0.1229	0.04790	44.6	-2.56	0.0138	0.05	-0.2194	-0.02635
**Ta**				31		-0.1680	0.04242	30.6	-3.96	0.0004	0.05	-0.2546	-0.08147
**Ta**				34		0	.	.	.	.	.	.	.
**T*S**	A				0	0.07201	0.06820	8.83	1.06	0.3191	0.05	-0.08272	0.2267
**T*S**	A				1	0	.	.	.	.	.	.	.
**T*S**	C				0	0	.	.	.	.	.	.	.
**T*S**	C				1	0	.	.	.	.	.	.	.
**Ta*T**	A			25		-0.02877	0.06658	41.6	-0.43	0.6679	0.05	-0.1632	0.1056
**Ta*T**	C			25		0	.	.	.	.	.	.	.
**Ta*T**	A			28		-0.03839	0.06156	42.3	-0.62	0.5362	0.05	-0.1626	0.08582
**Ta*T**	C			28		0	.	.	.	.	.	.	.
**Ta*T**	A			31		0.005508	0.05531	24.8	0.10	0.9215	0.05	-0.1084	0.1195
**Ta*T**	C			31		0	.	.	.	.	.	.	.
**Ta*T**	A			34		0	.	.	.	.	.	.	.
**Ta*T**	C			34		0	.	.	.	.	.	.	.
**Ta*S**				25	0	0.08554	0.06177	40.7	1.38	0.1736	0.05	-0.03922	0.2103
**Ta*S**				25	1	0	.	.	.	.	.	.	.
**Ta*S**				28	0	0.08063	0.05631	30.2	1.43	0.1625	0.05	-0.03434	0.1956
**Ta*S**				28	1	0	.	.	.	.	.	.	.
**Ta*S**				31	0	0.1089	0.04916	41.3	2.22	0.0323	0.05	0.009671	0.2082
**Ta*S**				31	1	0	.	.	.	.	.	.	.
**Ta*S**				34	0	0	.	.	.	.	.	.	.
**Ta*S**				34	1	0	.	.	.	.	.	.	.
**Gen**		Y				0.02400	0.04216	51.5	0.57	0.5716	0.05	-0.06062	0.1086
**Gen**		Z				0	.	.	.	.	.	.	.
**Timing**			Afternoon			-0.01721	0.02924	52.9	-0.59	0.5587	0.05	-0.07587	0.04145
**Timing**			Morning			0	.	.	.	.	.	.	.
**MB0**						0.03499	0.005783	52.1	6.05	<.0001	0.05	0.02338	0.04659
**Age**						-0.00073	0.000701	52.2	-1.04	0.3015	0.05	-0.00214	0.000675

Type 3 Tests of Fixed Effects				
**Effect**	**Num DF**	**Den DF**	**F Value**	**Pr > F**
**T**	1	19.4	7.65	0.0121
**S**	1	19.3	0.06	0.8070
**Ta**	3	8.53	12.08	0.0020
**T*S**	1	8.83	1.11	0.3191
**Ta*T**	3	8.48	0.32	0.8120
**Ta*S**	3	42.6	1.66	0.1899
**Gen**	1	51.5	0.32	0.5716
**Timing**	1	52.9	0.35	0.5587
**MB0**	1	52.1	36.60	<.0001
**Age**	1	52.2	1.09	0.3015

Least Squares Means															
**Effect**	**T**	**Gen**	**Timing**	**Ta**	**S**	**MB0**	**Age**	**Estimate**	**Standard Error**	**DF**	**t Value**	**Pr > |t|**	**Alpha**	**Lower**	**Upper**
**T**	A					24.68	140.44	1.2245	0.02994	21.7	40.89	<.0001	0.05	1.1624	1.2867
**T**	C					24.68	140.44	1.1087	0.02714	14.4	40.85	<.0001	0.05	1.0507	1.1668
**S**					0	24.68	140.44	1.1718	0.02894	18.5	40.50	<.0001	0.05	1.1112	1.2325
**S**					1	24.68	140.44	1.1614	0.02826	17.4	41.09	<.0001	0.05	1.1019	1.2210
**T*S**	A				0	24.68	140.44	1.2477	0.04026	18.4	30.99	<.0001	0.05	1.1633	1.3322
**T*S**	A				1	24.68	140.44	1.2013	0.04066	18.4	29.55	<.0001	0.05	1.1160	1.2866
**T*S**	C				0	24.68	140.44	1.0959	0.04010	15.5	27.33	<.0001	0.05	1.0107	1.1812
**T*S**	C				1	24.68	140.44	1.1215	0.03615	12.1	31.02	<.0001	0.05	1.0429	1.2002
**Ta*T**	A			25		24.68	140.44	1.2493	0.02963	12.3	42.16	<.0001	0.05	1.1849	1.3137
**Ta*T**	C			25		24.68	140.44	1.1468	0.02801	9.8	40.94	<.0001	0.05	1.0842	1.2094
**Ta*T**	A			28		24.68	140.44	1.1717	0.03170	16.9	36.96	<.0001	0.05	1.1048	1.2386
**Ta*T**	C			28		24.68	140.44	1.0789	0.02959	12.8	36.46	<.0001	0.05	1.0148	1.1429
**Ta*T**	A			31		24.68	140.44	1.1845	0.04139	38.5	28.62	<.0001	0.05	1.1008	1.2683
**Ta*T**	C			31		24.68	140.44	1.0478	0.03675	27.8	28.52	<.0001	0.05	0.9725	1.1231
**Ta*T**	A			34		24.68	140.44	1.2926	0.04924	47.4	26.25	<.0001	0.05	1.1936	1.3916
**Ta*T**	C			34		24.68	140.44	1.1614	0.04209	40.4	27.59	<.0001	0.05	1.0764	1.2464
**Ta*S**				25	0	24.68	140.44	1.2116	0.02852	12.8	42.48	<.0001	0.05	1.1499	1.2734
**Ta*S**				25	1	24.68	140.44	1.1845	0.02765	11.7	42.83	<.0001	0.05	1.1241	1.2449
**Ta*S**				28	0	24.68	140.44	1.1364	0.03023	16.2	37.59	<.0001	0.05	1.0724	1.2004

Least Squares Means															
**Effect**	**T**	**Gen**	**Timing**	**Ta**	**S**	**MB0**	**Age**	**Estimate**	**Standard Error**	**DF**	**t Value**	**Pr > |t|**	**Alpha**	**Lower**	**Upper**
**Ta*S**				28	1	24.68	140.44	1.1141	0.02955	15.5	37.71	<.0001	0.05	1.0513	1.1770
**Ta*S**				31	0	24.68	140.44	1.1415	0.03894	38.1	29.31	<.0001	0.05	1.0626	1.2203
**Ta*S**				31	1	24.68	140.44	1.0909	0.03791	35.8	28.78	<.0001	0.05	1.0140	1.1678
**Ta*S**				34	0	24.68	140.44	1.1978	0.04579	44.4	26.16	<.0001	0.05	1.1056	1.2901
**Ta*S**				34	1	24.68	140.44	1.2562	0.04437	44.2	28.31	<.0001	0.05	1.1668	1.3456
**Gen**		Y				24.68	140.44	1.1786	0.02935	46.7	40.16	<.0001	0.05	1.1196	1.2377
**Gen**		Z				24.68	140.44	1.1546	0.02800	42.2	41.24	<.0001	0.05	1.0981	1.2111
**Timing**			Afternoon			24.68	140.44	1.1580	0.02460	29.9	47.08	<.0001	0.05	1.1078	1.2083
**Timing**			Morning			24.68	140.44	1.1752	0.02407	31.6	48.83	<.0001	0.05	1.1262	1.2243
**T**	A					25.00	140.00	1.2360	0.02960	21.1	41.76	<.0001	0.05	1.1745	1.2976
**T**	C					25.00	140.00	1.1202	0.02761	15	40.58	<.0001	0.05	1.0614	1.1791
**S**					0	25.00	140.00	1.1833	0.02957	19.5	40.01	<.0001	0.05	1.1215	1.2451
**S**					1	25.00	140.00	1.1729	0.02769	16.5	42.36	<.0001	0.05	1.1144	1.2315
**Ta**				25		25.00	140.00	1.2096	0.01971	10	61.37	<.0001	0.05	1.1656	1.2535
**Ta**				28		25.00	140.00	1.1368	0.02099	13.5	54.15	<.0001	0.05	1.0916	1.1819
**Ta**				31		25.00	140.00	1.1277	0.02714	31.9	41.55	<.0001	0.05	1.0724	1.1830
**Ta**				34		25.00	140.00	1.2385	0.03202	43.7	38.67	<.0001	0.05	1.1740	1.3031
**T*S**	A				0	25.00	140.00	1.2592	0.04035	18.5	31.21	<.0001	0.05	1.1746	1.3438
**T*S**	A				1	25.00	140.00	1.2128	0.04006	17.7	30.28	<.0001	0.05	1.1286	1.2971
**T*S**	C				0	25.00	140.00	1.1074	0.04093	16.3	27.06	<.0001	0.05	1.0208	1.1941
**T*S**	C				1	25.00	140.00	1.1330	0.03593	11.9	31.54	<.0001	0.05	1.0547	1.2114
**Ta*T**	A			25		25.00	140.00	1.2608	0.02928	11.9	43.06	<.0001	0.05	1.1969	1.3247
**Ta*T**	C			25		25.00	140.00	1.1583	0.02848	10.3	40.67	<.0001	0.05	1.0951	1.2216
**Ta*T**	A			28		25.00	140.00	1.1832	0.03135	16.4	37.74	<.0001	0.05	1.1169	1.2495
**Ta*T**	C			28		25.00	140.00	1.0904	0.03002	13.3	36.33	<.0001	0.05	1.0257	1.1551
**Ta*T**	A			31		25.00	140.00	1.1961	0.04113	38	29.08	<.0001	0.05	1.1128	1.2793
**Ta*T**	C			31		25.00	140.00	1.0593	0.03708	28.4	28.57	<.0001	0.05	0.9834	1.1352
**Ta*T**	A			34		25.00	140.00	1.3041	0.04906	47.1	26.58	<.0001	0.05	1.2054	1.4028
**Ta*T**	C			34		25.00	140.00	1.1729	0.04239	41.1	27.67	<.0001	0.05	1.0873	1.2585
**Ta*S**				25	0	25.00	140.00	1.2231	0.02918	13.6	41.92	<.0001	0.05	1.1604	1.2859
**Ta*S**				25	1	25.00	140.00	1.1960	0.02707	11	44.18	<.0001	0.05	1.1364	1.2555
**Ta*S**				28	0	25.00	140.00	1.1479	0.03083	17.1	37.24	<.0001	0.05	1.0829	1.2129
**Ta*S**				28	1	25.00	140.00	1.1257	0.02899	14.6	38.83	<.0001	0.05	1.0637	1.1876
**Ta*S**				31	0	25.00	140.00	1.1530	0.03940	39	29.26	<.0001	0.05	1.0733	1.2327
**Ta*S**				31	1	25.00	140.00	1.1024	0.03747	34.9	29.42	<.0001	0.05	1.0263	1.1785

Least Squares Means														
**Effect**	**T**	**Gen**	**Timing**	**Ta**	**S**	**MB0**	**Age**	**Estimate**	**Standard Error**	**DF**	**t Value**	**Pr > |t|**	**Alpha**	**Lower**	**Upper**
**Ta*S**				34	0	25.00	140.00	1.2093	0.04621	45.6	26.17	<.0001	0.05	1.1163	1.3024
**Ta*S**				34	1	25.00	140.00	1.2677	0.04401	43.3	28.80	<.0001	0.05	1.1790	1.3565
**Gen**		Y				25.00	140.00	1.1901	0.02986	47.3	39.86	<.0001	0.05	1.1301	1.2502
**Gen**		Z				25.00	140.00	1.1661	0.02755	41.4	42.34	<.0001	0.05	1.1105	1.2218
**Timing**			Afternoon			25.00	140.00	1.1695	0.02476	30.5	47.24	<.0001	0.05	1.1190	1.2201
**Timing**			Morning			25.00	140.00	1.1867	0.02401	31	49.43	<.0001	0.05	1.1378	1.2357
**Ta**				25		24.68	140.44	1.1980	0.01964	9.91	61.01	<.0001	0.05	1.1542	1.2418
**Ta**				28		24.68	140.44	1.1253	0.02095	13.5	53.71	<.0001	0.05	1.0802	1.1704
**Ta**				31		24.68	140.44	1.1162	0.02711	31.8	41.17	<.0001	0.05	1.0609	1.1714
**Ta**				34		24.68	140.44	1.2270	0.03197	43.4	38.38	<.0001	0.05	1.1626	1.2915

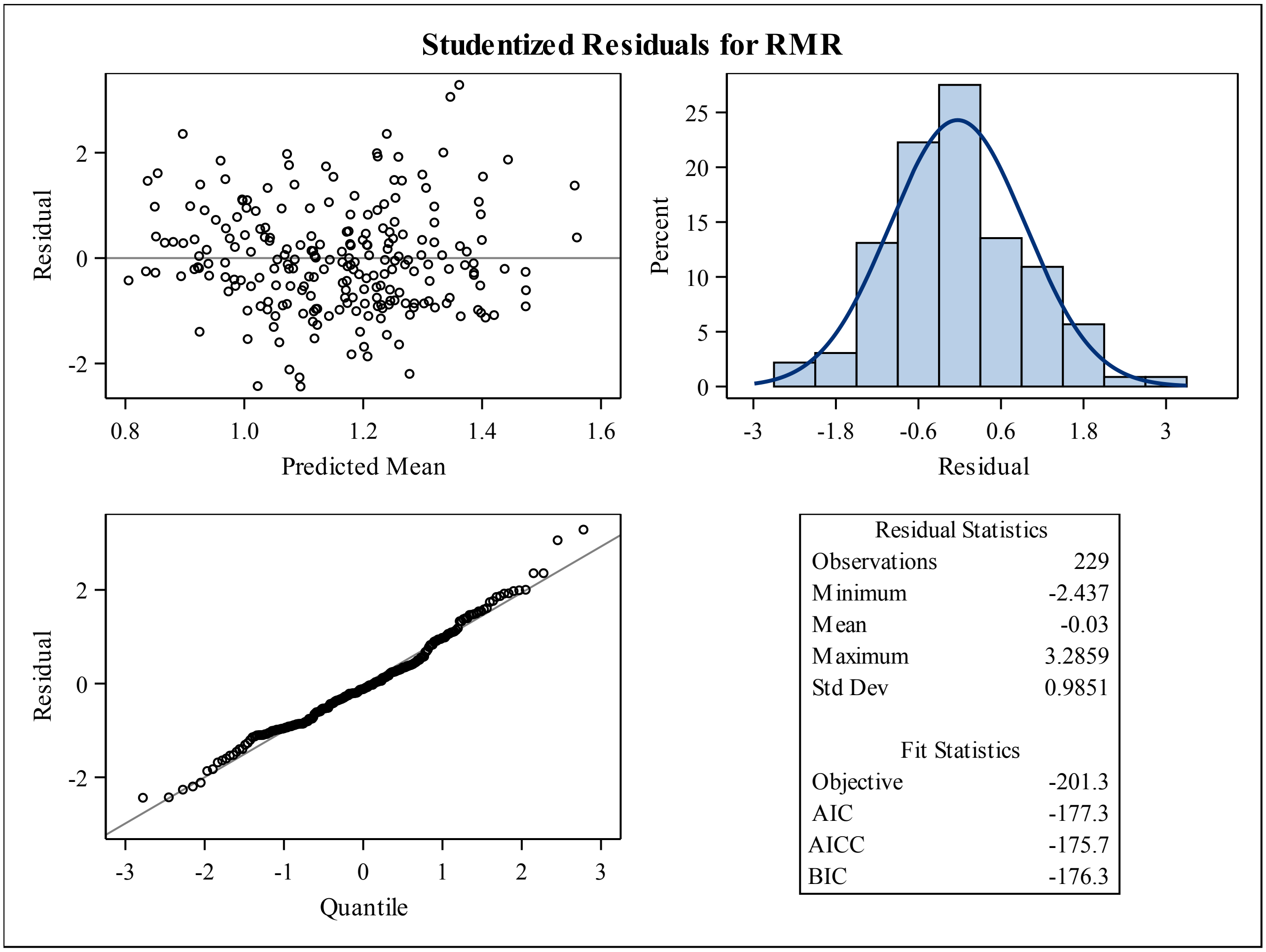

3.3. CT. Output from SAS (v. 9.3) Repeated Measures Model for CT for combined results from the trial performed at temperatures around the TNZ (25, 28, 31, and 34°C).

Model Information	
**Data Set**	WORK.THERMLONG
**Dependent Variable**	CT
**Covariance Structures**	Variance Components, Unstructured
**Subject Effect**	ID
**Estimation Method**	REML
**Residual Variance Method**	None
**Fixed Effects SE Method**	Model-Based
**Degrees of Freedom Method**	Satterthwaite

Class Level Information		
**Class**	**Levels**	**Values**
**Ta**	4	25 28 31 34
**T**	2	A C
**S**	2	0 1
**L**	8	A1 A2 A3 A4 C1 C2 C3 C4
**Gen**	2	Y Z
**Timing**	2	Afternoon Morning
**ID**	61	Y49489 Y49560 Y49937 Y50477 Y50676 Y50901 Y50954 Y50961 Y51184 Y51319 Y51482 Y51577 Y51662 Y51745 Y51767 Y51798 Y51821 Y51870 Y52096 Y52119 Y52321 Y52640 Y52843 Y52852 Y53007 Y53011 Y53143 Y53414 Y53504 Y53514 Y53605 Z54929 Z55058 Z55209 Z55222 Z55274 Z55665 Z55667 Z55702 Z55739 Z55741 Z55839 Z55851 Z55968 Z56223 Z56442 Z56494 Z56531 Z56557 Z56568 Z56661 Z56675 Z56787 Z56885 Z56996 Z60028 Z60236 Z60558 Z60607 Z60720 Z60756

Dimensions	
**Covariance Parameters**	13
**Columns in X**	35
**Columns in Z**	56
**Subjects**	1
**Max Obs per Subject**	215

Number of Observations	
Number of Observations Read	215
Number of Observations Used	215
Number of Observations Not Used	0

Covariance Parameter Estimates								
Cov Parm	Subject	Estimate	Standard Error	Z Value	Pr Z	Alpha	Lower	Upper
L(T)		0	.	.	.	.	.	.
S*L(T)		7.536E-6	8.302E-6	0.91	0.1820	0.05	1.878E-6	0.000587
Ta*L(T)		0	.	.	.	.	.	.
UN(1,1)	ID	0.000053	0.000011	4.70	<.0001	0.05	0.000036	0.000085
UN(2,1)	ID	0.000052	0.000015	3.49	0.0005	0.05	0.000023	0.000082
UN(2,2)	ID	0.000129	0.000027	4.80	<.0001	0.05	0.000089	0.000203
UN(3,1)	ID	0.000068	0.000028	2.43	0.0152	0.05	0.000013	0.000123
UN(3,2)	ID	0.000131	0.000044	2.97	0.0030	0.05	0.000045	0.000218
UN(3,3)	ID	0.000460	0.000101	4.57	<.0001	0.05	0.000312	0.000744
UN(4,1)	ID	-0.00002	0.000042	-0.51	0.6107	0.05	-0.00010	0.000060
UN(4,2)	ID	6.591E-6	0.000070	0.09	0.9253	0.05	-0.00013	0.000144
UN(4,3)	ID	0.000422	0.000134	3.14	0.0017	0.05	0.000159	0.000685
UN(4,4)	ID	0.001207	0.000256	4.72	<.0001	0.05	0.000829	0.001920

Fit Statistics	
-2 Res Log Likelihood	-1097.4
AIC (Smaller is Better)	-1075.4
AICC (Smaller is Better)	-1073.9
BIC (Smaller is Better)	-1074.5

Solution for Fixed Effects													
Effect	T	Gen	Timing	Ta	S	Estimate	Standard Error	DF	t Value	Pr > |t|	Alpha	Lower	Upper
Intercept						0.1878	0.01213	86.9	15.47	<.0001	0.05	0.1637	0.2119
T	A					-0.00358	0.01024	53.5	-0.35	0.7281	0.05	-0.02410	0.01695
T	C					0	.	.	.	.	.	.	.
S					0	-0.01285	0.01010	53.2	-1.27	0.2088	0.05	-0.03310	0.007407

Solution for Fixed Effects													
**Effect**	**T**	**Gen**	**Timing**	**Ta**	**S**	**Estimate**	**Standard Error**	**DF**	**t Value**	**Pr > |t|**	**Alpha**	**Lower**	**Upper**
**S**					1	0	.	.	.	.	.	.	.
**Ta**				25		-0.1528	0.008110	49.9	-18.84	<.0001	0.05	-0.1691	-0.1365
**Ta**				28		-0.1310	0.008179	47	-16.02	<.0001	0.05	-0.1475	-0.1145
**Ta**				31		-0.09064	0.006641	42.4	-13.65	<.0001	0.05	-0.1040	-0.07724
**Ta**				34		0	.	.	.	.	.	.	.
**T*S**	A				0	0.01058	0.004511	11.9	2.35	0.0372	0.05	0.000744	0.02042
**T*S**	A				1	0	.	.	.	.	.	.	.
**T*S**	C				0	0	.	.	.	.	.	.	.
**T*S**	C				1	0	.	.	.	.	.	.	.
**Ta*T**	A			25		0.004590	0.01021	51.4	0.45	0.6549	0.05	-0.01590	0.02508
**Ta*T**	C			25		0	.	.	.	.	.	.	.
**Ta*T**	A			28		0.007320	0.01029	48.7	0.71	0.4801	0.05	-0.01335	0.02799
**Ta*T**	C			28		0	.	.	.	.	.	.	.
**Ta*T**	A			31		0.01255	0.008415	43.2	1.49	0.1432	0.05	-0.00442	0.02952
**Ta*T**	C			31		0	.	.	.	.	.	.	.
**Ta*T**	A			34		0	.	.	.	.	.	.	.
**Ta*T**	C			34		0	.	.	.	.	.	.	.
**Ta*S**				25	0	0.009623	0.01007	51.2	0.96	0.3436	0.05	-0.01058	0.02983
**Ta*S**				25	1	0	.	.	.	.	.	.	.
**Ta*S**				28	0	0.005884	0.01014	48.4	0.58	0.5645	0.05	-0.01450	0.02627
**Ta*S**				28	1	0	.	.	.	.	.	.	.
**Ta*S**				31	0	0.002379	0.008274	42.8	0.29	0.7751	0.05	-0.01431	0.01907
**Ta*S**				31	1	0	.	.	.	.	.	.	.
**Ta*S**				34	0	0	.	.	.	.	.	.	.
**Ta*S**				34	1	0	.	.	.	.	.	.	.
**Gen**		Y				0.000966	0.002672	49.5	0.36	0.7192	0.05	-0.00440	0.006335
**Gen**		Z				0	.	.	.	.	.	.	.
**Timing**			Afternoon			0.001077	0.001877	47.9	0.57	0.5686	0.05	-0.00270	0.004851
**Timing**			Morning			0	.	.	.	.	.	.	.
**MB0**						0.002566	0.000380	48.9	6.75	<.0001	0.05	0.001802	0.003330
**Age**						-0.00006	0.000045	49.4	-1.39	0.1704	0.05	-0.00015	0.000028

Type 3 Tests of Fixed Effects				
Effect	Num DF	Den DF	F Value	Pr > F
T	1	41.3	3.46	0.0701
S	1	40.9	0.54	0.4651
Ta	3	49.8	356.27	<.0001
T*S	1	11.9	5.50	0.0372
Ta*T	3	49.8	1.66	0.1874
Ta*S	3	49.6	1.15	0.3395
Gen	1	49.5	0.13	0.7192
Timing	1	47.9	0.33	0.5686
MB0	1	48.9	45.52	<.0001
Age	1	49.4	1.94	0.1704

Least Squares Means															
Effect	T	Gen	Timing	Ta	S	MB0	Age	Estimate	Standard Error	DF	t Value	Pr > |t|	Alpha	Lower	Upper
T	A					24.66	140.03	0.1533	0.003123	43.4	49.10	<.0001	0.05	0.1470	0.1596
T	C					24.66	140.03	0.1455	0.002730	35.5	53.30	<.0001	0.05	0.1400	0.1510
S					0	24.66	140.03	0.1479	0.002996	40.7	49.36	<.0001	0.05	0.1418	0.1539
S					1	24.66	140.03	0.1510	0.002852	38.5	52.93	<.0001	0.05	0.1452	0.1567
T*S	A				0	24.66	140.03	0.1544	0.003984	42.9	38.76	<.0001	0.05	0.1464	0.1625
T*S	A				1	24.66	140.03	0.1522	0.003881	41.8	39.23	<.0001	0.05	0.1444	0.1601
T*S	C				0	24.66	140.03	0.1413	0.003729	37.2	37.90	<.0001	0.05	0.1338	0.1489
T*S	C				1	24.66	140.03	0.1497	0.003491	32.6	42.87	<.0001	0.05	0.1426	0.1568
Ta*T	A			25		24.66	140.03	0.09520	0.001804	17.1	52.76	<.0001	0.05	0.09140	0.09901
Ta*T	C			25		24.66	140.03	0.08890	0.001715	14.3	51.83	<.0001	0.05	0.08523	0.09257
Ta*T	A			28		24.66	140.03	0.1178	0.002463	34.4	47.85	<.0001	0.05	0.1128	0.1228
Ta*T	C			28		24.66	140.03	0.1088	0.002312	29.6	47.06	<.0001	0.05	0.1041	0.1135
Ta*T	A			31		24.66	140.03	0.1617	0.004527	48.7	35.71	<.0001	0.05	0.1526	0.1708
Ta*T	C			31		24.66	140.03	0.1474	0.003918	43.2	37.63	<.0001	0.05	0.1395	0.1553
Ta*T	A			34		24.66	140.03	0.2386	0.007678	51.1	31.07	<.0001	0.05	0.2232	0.2540
Ta*T	C			34		24.66	140.03	0.2369	0.006427	50	36.85	<.0001	0.05	0.2240	0.2498
Ta*S				25	0	24.66	140.03	0.09308	0.001802	16.9	51.65	<.0001	0.05	0.08928	0.09689
Ta*S				25	1	24.66	140.03	0.09102	0.001719	14.7	52.94	<.0001	0.05	0.08735	0.09469
Ta*S				28	0	24.66	140.03	0.1125	0.002446	33.1	45.99	<.0001	0.05	0.1075	0.1175

Least Squares Means														
**Effect**	**T**	**Gen**	**Timing**	**Ta**	**S**	**MB0**	**Age**	**Estimate**	**Standard Error**	**DF**	**t Value**	**Pr > |t|**	**Alpha**	**Lower**	**Upper**
**Ta*S**				28	1	24.66	140.03	0.1142	0.002322	30.4	49.18	<.0001	0.05	0.1094	0.1189
**Ta*S**				31	0	24.66	140.03	0.1520	0.004301	45.8	35.33	<.0001	0.05	0.1433	0.1606
**Ta*S**				31	1	24.66	140.03	0.1571	0.004125	45.8	38.10	<.0001	0.05	0.1488	0.1654
**Ta*S**				34	0	24.66	140.03	0.2339	0.007240	50.9	32.31	<.0001	0.05	0.2194	0.2485
**Ta*S**				34	1	24.66	140.03	0.2415	0.006830	50.6	35.36	<.0001	0.05	0.2278	0.2552
**Gen**		Y				24.66	140.03	0.1499	0.002472	66.9	60.63	<.0001	0.05	0.1450	0.1548
**Gen**		Z				24.66	140.03	0.1489	0.002409	57.7	61.83	<.0001	0.05	0.1441	0.1537
**Timing**			Afternoon			24.66	140.03	0.1499	0.002248	51.5	66.70	<.0001	0.05	0.1454	0.1545
**Timing**			Morning			24.66	140.03	0.1489	0.002248	50.6	66.24	<.0001	0.05	0.1444	0.1534
**T**	A					25.00	140.00	0.1542	0.003107	42.9	49.64	<.0001	0.05	0.1479	0.1605
**T**	C					25.00	140.00	0.1464	0.002752	36.3	53.19	<.0001	0.05	0.1408	0.1520
**S**					0	25.00	140.00	0.1487	0.003020	41.6	49.26	<.0001	0.05	0.1426	0.1548
**S**					1	25.00	140.00	0.1518	0.002830	37.8	53.64	<.0001	0.05	0.1461	0.1576
**Ta**				25		25.00	140.00	0.09293	0.001194	14.2	77.86	<.0001	0.05	0.09037	0.09548
**Ta**				28		25.00	140.00	0.1142	0.001647	30.1	69.33	<.0001	0.05	0.1108	0.1176
**Ta**				31		25.00	140.00	0.1554	0.002968	45.1	52.36	<.0001	0.05	0.1494	0.1614
**Ta**				34		25.00	140.00	0.2386	0.005006	50.4	47.66	<.0001	0.05	0.2285	0.2486
**T*S**	A				0	25.00	140.00	0.1553	0.003986	43	38.97	<.0001	0.05	0.1473	0.1633
**T*S**	A				1	25.00	140.00	0.1531	0.003854	41.1	39.73	<.0001	0.05	0.1453	0.1609
**T*S**	C				0	25.00	140.00	0.1422	0.003766	38.1	37.76	<.0001	0.05	0.1346	0.1498
**T*S**	C				1	25.00	140.00	0.1506	0.003486	32.5	43.20	<.0001	0.05	0.1435	0.1577
**Ta*T**	A			25		25.00	140.00	0.09608	0.001775	16.4	54.13	<.0001	0.05	0.09232	0.09983
**Ta*T**	C			25		25.00	140.00	0.08978	0.001752	15.1	51.24	<.0001	0.05	0.08604	0.09351
**Ta*T**	A			28		25.00	140.00	0.1187	0.002439	33.5	48.67	<.0001	0.05	0.1138	0.1237
**Ta*T**	C			28		25.00	140.00	0.1097	0.002338	30.5	46.91	<.0001	0.05	0.1049	0.1145
**Ta*T**	A			31		25.00	140.00	0.1626	0.004513	48.2	36.02	<.0001	0.05	0.1535	0.1716
**Ta*T**	C			31		25.00	140.00	0.1483	0.003932	43.7	37.71	<.0001	0.05	0.1404	0.1562
**Ta*T**	A			34		25.00	140.00	0.2395	0.007674	51	31.20	<.0001	0.05	0.2240	0.2549
**Ta*T**	C			34		25.00	140.00	0.2377	0.006436	50.2	36.94	<.0001	0.05	0.2248	0.2507
**Ta*S**				25	0	25.00	140.00	0.09396	0.001841	17.8	51.03	<.0001	0.05	0.09009	0.09783
**Ta*S**				25	1	25.00	140.00	0.09190	0.001684	13.8	54.56	<.0001	0.05	0.08828	0.09551
**Ta*S**				28	0	25.00	140.00	0.1134	0.002473	34	45.83	<.0001	0.05	0.1083	0.1184
**Ta*S**				28	1	25.00	140.00	0.1150	0.002294	29.4	50.15	<.0001	0.05	0.1104	0.1197
**Ta*S**				31	0	25.00	140.00	0.1528	0.004317	46.4	35.40	<.0001	0.05	0.1441	0.1615
**Ta*S**				31	1	25.00	140.00	0.1580	0.004107	45.3	38.47	<.0001	0.05	0.1497	0.1663

Least Squares Means														
**Effect**	**T**	**Gen**	**Timing**	**Ta**	**S**	**MB0**	**Age**	**Estimate**	**Standard Error**	**DF**	**t Value**	**Pr > |t|**	**Alpha**	**Lower**	**Upper**
**Ta*S**				34	0	25.00	140.00	0.2348	0.007251	51.1	32.38	<.0001	0.05	0.2203	0.2494
**Ta*S**				34	1	25.00	140.00	0.2424	0.006822	50.4	35.53	<.0001	0.05	0.2287	0.2561
**Gen**		Y				25.00	140.00	0.1508	0.002490	67.6	60.56	<.0001	0.05	0.1458	0.1557
**Gen**		Z				25.00	140.00	0.1498	0.002395	57.3	62.55	<.0001	0.05	0.1450	0.1546
**Timing**			Afternoon			25.00	140.00	0.1508	0.002255	52	66.90	<.0001	0.05	0.1463	0.1553
**Timing**			Morning			25.00	140.00	0.1497	0.002245	50.4	66.69	<.0001	0.05	0.1452	0.1543
**Ta**				25		24.66	140.03	0.09205	0.001189	14.1	77.43	<.0001	0.05	0.08950	0.09460
**Ta**				28		24.66	140.03	0.1133	0.001646	30.1	68.85	<.0001	0.05	0.1100	0.1167
**Ta**				31		24.66	140.03	0.1545	0.002969	45.1	52.05	<.0001	0.05	0.1486	0.1605
**Ta**				34		24.66	140.03	0.2377	0.005003	50.3	47.52	<.0001	0.05	0.2277	0.2478

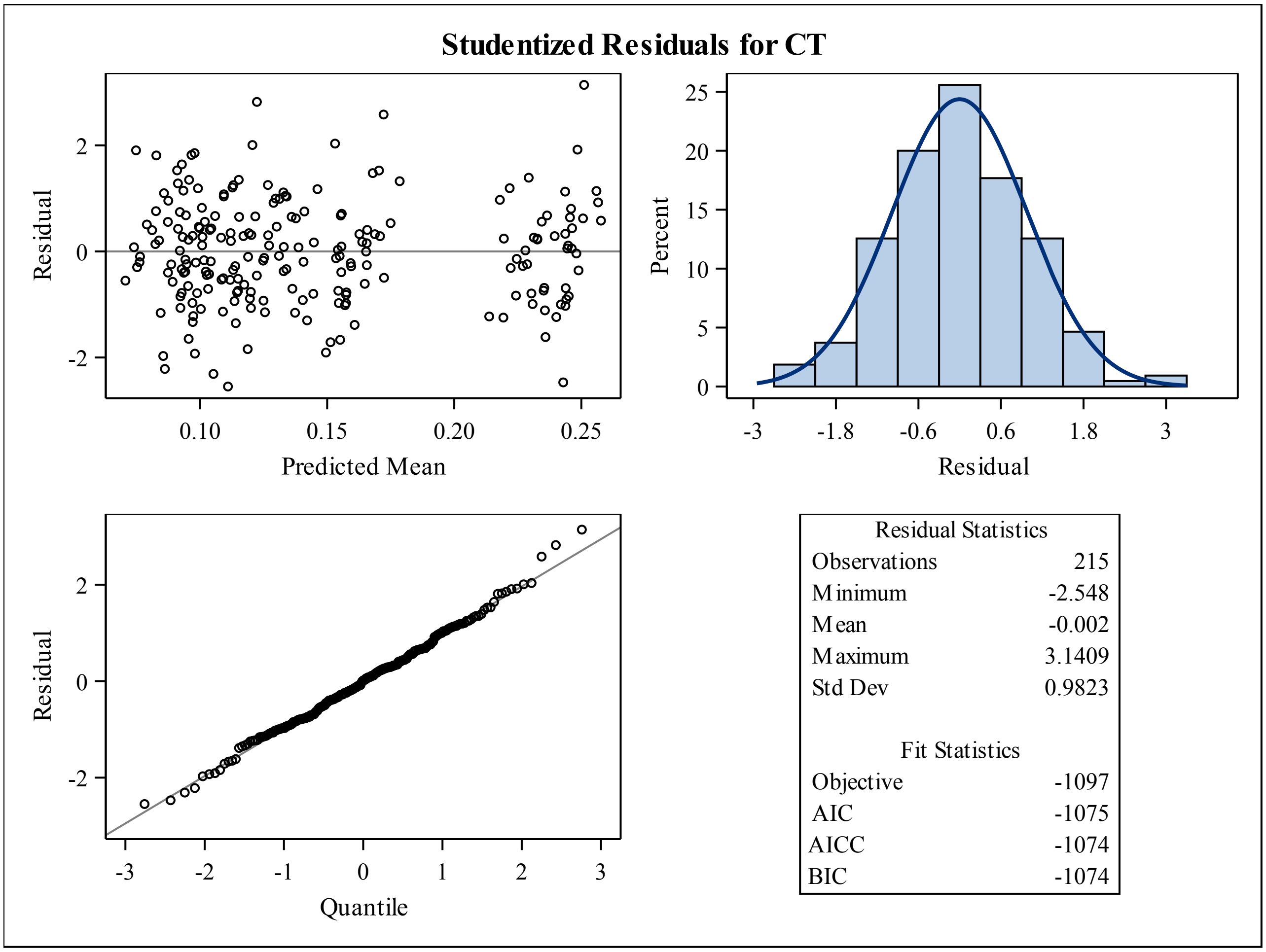

Part 4. Stage-regression, mixed nonlinear model for fitting parameters of the thermoregulatory curve

To analyze the main characteristics of the thermoregulatory curve we applied a stage-regression model, implemented in SAS mixed nonlinear procedure (NLMIXED). The data available could not allow a reliable estimate of the UCT and therefore the analyses were performed only for the temperature range of 10 to 31°C, and was focused on the question of whether selection affected LCT, the level of RMR above LCT (RMR_{TNZ}; conceptually equivalent to BMR), and the slope of the relationship between RMR and T_a below LCT (C_t; i.e. another measure of thermal conductance). The model includes also random effects of individuals (ID) and residual error e, each assumed to have a normal distribution. The logic model was as follows:

$$RMR = \begin{cases} \text{for } T_a >= LCT\text{:} & RMR_{TNZ} + ID + e \\ \text{for } T_a < LCT\text{:} & RMR_{TNZ} + CT \times (LCT - T_a) + ID + e \end{cases}$$

However, all of the three parameters of the model are known to depend on body mass. Therefore, they were introduced to the model as linear functions of body mass (M_b), each with an intercept and a mass-slope coefficient:

a) $LCT = t_0 + t_m \times M_b$

b) $RMR_{TNZ} = b_0 + b_m \times M_b$

c) $C_t = c_0 + c_m \times M_b$

Finally, each of the six parameters were introduced to the model as either a value common for both of the selection directions or specific for the A and C lines. Thus, the initial "full" model had a total of 12 fixed parameters (in addition to two random effects), i.e. allowed not only difference in intercepts between the selection directions, but also heterogeneous mass-slopes. The model was then stepwise reduced, first by removing the coefficients responsible for differences in mass-slope coefficients (which resulted in a model with homogeneous mass-slopes), and then by removing other components. We first compared the models using AIC criterion, and then to formally test significance of difference in a particular parameter between the A and C lines a likelihood ratio test (LRT) was applied.

4.1. Main characteristics of the thermoregulatory curve. Output from SAS (v. 9.3) Mixed Nonlinear Procedure to analyze the main characteristics of the thermoregulatory curve (by means of a stage-regression model). Best model (according to AIC) is presented.

Specifications	
**Data Set**	WORK.THERMCURVE
**Dependent Variable**	RMR
**Distribution for Dependent Variable**	Normal
**Random Effects**	IDef
**Distribution for Random Effects**	Normal
**Subject Variable**	ID
**Optimization Technique**	Dual Quasi-Newton
**Integration Method**	Adaptive Gaussian Quadrature

Dimensions	
**Observations Used**	428
**Observations Not Used**	272
**Total Observations**	700
**Subjects**	140
**Max Obs per Subject**	5
**Parameters**	9
**Quadrature Points**	1

Fit Statistics	
**-2 Log Likelihood**	-331.1
**AIC (smaller is better)**	-313.1
**AICC (smaller is better)**	-312.7
**BIC (smaller is better)**	-286.7

Parameter Estimates								
**Parameter**	**Estimate**	**Standard Error**	**DF**	**t Value**	**Pr > |t|**	**95% Confidence Limits**		**Gradient**
**b0**	0.3257	0.1070	139	3.04	0.0028	0.1142	0.5372	-0.00003
**b0 diff. (A-C)**	0.1095	0.02984	139	3.67	0.0003	0.05050	0.1685	0.000749
**bm**	0.03237	0.004252	139	7.61	<.0001	0.02396	0.04077	-0.00083

Parameter Estimates								
Parameter	Estimate	Standard Error	DF	t Value	Pr > |t|	95% Confidence Limits		Gradient
**t0**	26.0963	0.2609	139	100.0	<.0001	25.5803	26.6122	0.000036
**c0**	0.05820	0.009017	139	6.45	<.0001	0.04037	0.07602	0.000620
**c0 diff. (A-C)**	-0.00589	0.002623	139	-2.25	0.0262	-0.01108	-0.00071	0.003917
**cm**	0.000794	0.000358	139	2.22	0.0281	0.000087	0.001502	0.12979
**ID variance**	0.008112	0.002126	139	3.82	0.0002	0.003910	0.01231	-0.00794
**s2 (residual)**	0.02123	0.001794	139	11.83	<.0001	0.01768	0.02478	0.024973
